# Associations of autozygosity with a broad range of human phenotypes

**DOI:** 10.1038/s41467-019-12283-6

**Published:** 2019-10-31

**Authors:** David W Clark, Yukinori Okada, Kristjan H S Moore, Dan Mason, Nicola Pirastu, Ilaria Gandin, Hannele Mattsson, Catriona L K Barnes, Kuang Lin, Jing Hua Zhao, Patrick Deelen, Rebecca Rohde, Claudia Schurmann, Xiuqing Guo, Franco Giulianini, Weihua Zhang, Carolina Medina-Gomez, Robert Karlsson, Yanchun Bao, Traci M Bartz, Clemens Baumbach, Ginevra Biino, Matthew J Bixley, Marco Brumat, Jin-Fang Chai, Tanguy Corre, Diana L Cousminer, Annelot M Dekker, David A Eccles, Kristel R van Eijk, Christian Fuchsberger, He Gao, Marine Germain, Scott D Gordon, Hugoline G de Haan, Sarah E Harris, Edith Hofer, Alicia Huerta-Chagoya, Catherine Igartua, Iris E Jansen, Yucheng Jia, Tim Kacprowski, Torgny Karlsson, Marcus E Kleber, Shengchao Alfred Li, Ruifang Li-Gao, Anubha Mahajan, Koichi Matsuda, Karina Meidtner, Weihua Meng, May E Montasser, Peter J van der Most, Matthias Munz, Teresa Nutile, Teemu Palviainen, Gauri Prasad, Rashmi B Prasad, Tallapragada Divya Sri Priyanka, Federica Rizzi, Erika Salvi, Bishwa R Sapkota, Daniel Shriner, Line Skotte, Melissa C Smart, Albert Vernon Smith, Ashley van der Spek, Cassandra N Spracklen, Rona J Strawbridge, Salman M Tajuddin, Stella Trompet, Constance Turman, Niek Verweij, Clara Viberti, Lihua Wang, Helen R Warren, Robyn E Wootton, Lisa R Yanek, Jie Yao, Noha A Yousri, Wei Zhao, Adebowale A Adeyemo, Saima Afaq, Carlos Alberto Aguilar-Salinas, Masato Akiyama, Matthew L Albert, Matthew A Allison, Maris Alver, Tin Aung, Fereidoun Azizi, Amy R Bentley, Heiner Boeing, Eric Boerwinkle, Judith B Borja, Gert J de Borst, Erwin P Bottinger, Linda Broer, Harry Campbell, Stephen Chanock, Miao-Li Chee, Guanjie Chen, Yii-Der I Chen, Zhengming Chen, Yen-Feng Chiu, Massimiliano Cocca, Francis S Collins, Maria Pina Concas, Janie Corley, Giovanni Cugliari, Rob M van Dam, Anna Damulina, Maryam S Daneshpour, Felix R Day, Graciela E Delgado, Klodian Dhana, Alexander S F Doney, Marcus Dörr, Ayo P Doumatey, Nduna Dzimiri, S Sunna Ebenesersdóttir, Joshua Elliott, Paul Elliott, Ralf Ewert, Janine F Felix, Krista Fischer, Barry I Freedman, Giorgia Girotto, Anuj Goel, Martin Gögele, Mark O Goodarzi, Mariaelisa Graff, Einat Granot-Hershkovitz, Francine Grodstein, Simonetta Guarrera, Daniel F Gudbjartsson, Kamran Guity, Bjarni Gunnarsson, Yu Guo, Saskia P Hagenaars, Christopher A Haiman, Avner Halevy, Tamara B Harris, Mehdi Hedayati, David A van Heel, Makoto Hirata, Imo Höfer, Chao Agnes Hsiung, Jinyan Huang, Yi-Jen Hung, M Arfan Ikram, Anuradha Jagadeesan, Pekka Jousilahti, Yoichiro Kamatani, Masahiro Kanai, Nicola D Kerrison, Thorsten Kessler, Kay-Tee Khaw, Chiea Chuen Khor, Dominique P V de Kleijn, Woon-Puay Koh, Ivana Kolcic, Peter Kraft, Bernhard K Krämer, Zoltán Kutalik, Johanna Kuusisto, Claudia Langenberg, Lenore J Launer, Deborah A Lawlor, I-Te Lee, Wen-Jane Lee, Markus M Lerch, Liming Li, Jianjun Liu, Marie Loh, Stephanie J London, Stephanie Loomis, Yingchang Lu, Jian’an Luan, Reedik Mägi, Ani W Manichaikul, Paolo Manunta, Gísli Másson, Nana Matoba, Xue W Mei, Christa Meisinger, Thomas Meitinger, Massimo Mezzavilla, Lili Milani, Iona Y Millwood, Yukihide Momozawa, Amy Moore, Pierre-Emmanuel Morange, Hortensia Moreno-Macías, Trevor A Mori, Alanna C Morrison, Taulant Muka, Yoshinori Murakami, Alison D Murray, Renée de Mutsert, Josyf C Mychaleckyj, Mike A Nalls, Matthias Nauck, Matt J Neville, Ilja M Nolte, Ken K Ong, Lorena Orozco, Sandosh Padmanabhan, Gunnar Pálsson, James S Pankow, Cristian Pattaro, Alison Pattie, Ozren Polasek, Neil Poulter, Peter P Pramstaller, Lluis Quintana-Murci, Katri Räikkönen, Sarju Ralhan, Dabeeru C Rao, Wouter van Rheenen, Stephen S Rich, Paul M Ridker, Cornelius A Rietveld, Antonietta Robino, Frank J A van Rooij, Daniela Ruggiero, Yasaman Saba, Charumathi Sabanayagam, Maria Sabater-Lleal, Cinzia Felicita Sala, Veikko Salomaa, Kevin Sandow, Helena Schmidt, Laura J Scott, William R Scott, Bahareh Sedaghati-Khayat, Bengt Sennblad, Jessica van Setten, Peter J Sever, Wayne H-H Sheu, Yuan Shi, Smeeta Shrestha, Sharvari Rahul Shukla, Jon K Sigurdsson, Timo Tonis Sikka, Jai Rup Singh, Blair H Smith, Alena Stančáková, Alice Stanton, John M Starr, Lilja Stefansdottir, Leon Straker, Patrick Sulem, Gardar Sveinbjornsson, Morris A Swertz, Adele M Taylor, Kent D Taylor, Natalie Terzikhan, Yih-Chung Tham, Gudmar Thorleifsson, Unnur Thorsteinsdottir, Annika Tillander, Russell P Tracy, Teresa Tusié-Luna, Ioanna Tzoulaki, Simona Vaccargiu, Jagadish Vangipurapu, Jan H Veldink, Veronique Vitart, Uwe Völker, Eero Vuoksimaa, Salma M Wakil, Melanie Waldenberger, Gurpreet S Wander, Ya Xing Wang, Nicholas J Wareham, Sarah Wild, Chittaranjan S Yajnik, Jian-Min Yuan, Lingyao Zeng, Liang Zhang, Jie Zhou, Najaf Amin, Folkert W Asselbergs, Stephan J L Bakker, Diane M Becker, Benjamin Lehne, David A Bennett, Leonard H van den Berg, Sonja I Berndt, Dwaipayan Bharadwaj, Lawrence F Bielak, Murielle Bochud, Mike Boehnke, Claude Bouchard, Jonathan P Bradfield, Jennifer A Brody, Archie Campbell, Shai Carmi, Mark J Caulfield, David Cesarini, John C Chambers, Giriraj Ratan Chandak, Ching-Yu Cheng, Marina Ciullo, Marilyn Cornelis, Daniele Cusi, George Davey Smith, Ian J Deary, Rajkumar Dorajoo, Cornelia M van Duijn, David Ellinghaus, Jeanette Erdmann, Johan G Eriksson, Evangelos Evangelou, Michele K Evans, Jessica D Faul, Bjarke Feenstra, Mary Feitosa, Sylvain Foisy, Andre Franke, Yechiel Friedlander, Paolo Gasparini, Christian Gieger, Clicerio Gonzalez, Philippe Goyette, Struan F A Grant, Lyn R Griffiths, Leif Groop, Vilmundur Gudnason, Ulf Gyllensten, Hakon Hakonarson, Anders Hamsten, Pim van der Harst, Chew-Kiat Heng, Andrew A Hicks, Hagit Hochner, Heikki Huikuri, Steven C Hunt, Vincent W V Jaddoe, Philip L De Jager, Magnus Johannesson, Åsa Johansson, Jost B Jonas, J Wouter Jukema, Juhani Junttila, Jaakko Kaprio, Sharon L. R. Kardia, Fredrik Karpe, Meena Kumari, Markku Laakso, Sander W van der Laan, Jari Lahti, Matthias Laudes, Rodney A Lea, Wolfgang Lieb, Thomas Lumley, Nicholas G Martin, Winfried März, Giuseppe Matullo, Mark I McCarthy, Sarah E Medland, Tony R Merriman, Andres Metspalu, Brian F Meyer, Karen L Mohlke, Grant W Montgomery, Dennis Mook-Kanamori, Patricia B Munroe, Kari E North, Dale R Nyholt, Jeffery R O’connell, Carole Ober, Albertine J Oldehinkel, Walter Palmas, Colin Palmer, Gerard G Pasterkamp, Etienne Patin, Craig E Pennell, Louis Perusse, Patricia A Peyser, Mario Pirastu, Tinca J. C. Polderman, David J Porteous, Danielle Posthuma, Bruce M Psaty, John D Rioux, Fernando Rivadeneira, Charles Rotimi, Jerome I Rotter, Igor Rudan, Hester M Den Ruijter, Dharambir K Sanghera, Naveed Sattar, Reinhold Schmidt, Matthias B Schulze, Heribert Schunkert, Robert A Scott, Alan R Shuldiner, Xueling Sim, Neil Small, Jennifer A Smith, Nona Sotoodehnia, E-Shyong Tai, Alexander Teumer, Nicholas J Timpson, Daniela Toniolo, David-Alexandre Tregouet, Tiinamaija Tuomi, Peter Vollenweider, Carol A Wang, David R Weir, John B Whitfield, Cisca Wijmenga, Tien-Yin Wong, John Wright, Jingyun Yang, Lei Yu, Babette S Zemel, Alan B Zonderman, Markus Perola, Patrik K. E. Magnusson, André G Uitterlinden, Jaspal S Kooner, Daniel I Chasman, Ruth J. F. Loos, Nora Franceschini, Lude Franke, Chris S Haley, Caroline Hayward, Robin G Walters, John R. B. Perry, Tōnu Esko, Agnar Helgason, Kari Stefansson, Peter K Joshi, Michiaki Kubo, James F Wilson

**Affiliations:** 1 Centre for Global Health Research, Usher Institute, University of Edinburgh, Edinburgh, EH8 9AG Scotland; 2 Department of Statistical Genetics, Osaka University Graduate School of Medicine, Suita, Osaka, 565-0871 Japan; 3 Laboratory for Statistical Analysis, RIKEN Center for Integrative Medical Sciences, Yokohama, Kanagawa 230-0045 Japan; 4 Laboratory of Statistical Immunology, Immunology Frontier Research Center (WPI-IFReC), Osaka University, Suita, Osaka, 565-0871 Japan; 5 deCODE genetics/Amgen Inc., Reykjavik 101, Iceland; 6 Bradford Institute for Health Research, Bradford Teaching Hospitals NHS Trust, Bradford, BD96RJ UK; 7 Research Unit, Area Science Park, Trieste, 34149 Italy; 8 Department of Medicine, Surgery and Health Sciences, University of Trieste, Trieste, Italy; 9 Unit of Public Health Solutions, National Institute for Health and Welfare, Helsinki, Finland; 10 Institute for Molecular Medicine Finland, University of Helsinki, Helsinki, Finland; 11 Nuffield Department of Population Health, University of Oxford, Oxford, OX3 7LF UK; 12 MRC Epidemiology Unit, University of Cambridge School of Clinical Medicine, Cambridge, CB2 0QQ UK; 13 Cardiovascular Epidemiology Unit, Department of Public health and Primary Care, University of Cambridge, Cambridge, CB1 8RN UK; 14 Department of Genetics, University Medical Centre Groningen, University of Groningen, Groningen, the Netherlands, Groningen, Groningen, 9700 RB The Netherlands; 15 Department of Epidemiology, Gillings School of Global Public Health, University of North Carolina, Chapel Hill, NC 27514 USA; 16 The Charles Bronfman Institute for Personalized Medicine, Ichan School of Medicine at Mount Sinai, New York, NY 10029 USA; 17 Division of Genomic Outcomes, Department of Pediatrics, The Institute for Translational Genomics and Population Sciences, LABioMed at Harbor-UCLA Medical Center, Torrance, California, 90502 USA; 18 Division of Preventive Medicine, Brigham and Women’s Hospital, Boston, MA 02215 USA; 19 Department of Epidemiology and Biostatistics, Imperial College London, London, W2 1PG UK; 20 Department of Cardiology, Ealing Hospital, Middlesex, Middlesex, UB1 3HW UK; 21 Department of Internal Medicine, Erasmus University Medical Center, Rotterdam, 3015 CN Netherlands; 22 Department of Epidemiology, Erasmus University Medical Center, Rotterdam, 3015 CN Netherlands; 23 The Generation R Study Group, Erasmus University Medical Center, Rotterdam, 3015 CN The Netherlands; 24 Department of Medical Epidemiology and Biostatistics, Karolinska Institutet, Stockholm, 17177 Sweden; 25 Institute for Social and Economic Research, University of Essex, Colchester, CO4 3SQ UK; 26 Cardiovascular Health Research Unit, Departments of Biostatistics and Medicine, University of Washington, Seattle, WA 98101 USA; 27 Research Unit of Molecular Epidemiology, Institute of Epidemiology, Helmholtz Zentrum München - German Research Center for Environmental Health, Neuherberg, 85764 Germany; 28 Institute of Molecular Genetics, National Research Council of Italy, Pavia, 27100 Italy; 29 Department of Biochemistry, University of Otago, Dunedin, 9054 New Zealand; 30 Saw Swee Hock School of Public Health, National University of Singapore, Singapore, Singapore, 117549 Singapore; 31 Department of Computational Biology, University of Lausanne, Lausanne, 1011 Switzerland; 32 Center for Primary Care and Public Health (Unisanté), University of Lausanne, Lausanne, Switzerland; 33 Swiss Institute of Bioinformatics, Lausanne, 1015 Switzerland; 34 Division of Human Genetics, Children’s Hospital of Philadelphia, Philadelphia, PA 19104 USA; 35 Department of Genetics, Perelman School of Medicine, University of Pennsylvania, Philadelphia, PA 19104 USA; 36 Department of Neurology, Brain Centre Rudolf Magnus, University Medical Centre Utrecht, Utrecht University, Utrecht, 3584 CX The Netherlands; 37 Genomics Research Centre, School of Biomedical Sciences, Institute of Health and Biomedical Innovation, Queensland University of Technology, Brisbane, Queensland 4059 Australia; 38 Malaghan Institute of Medical Research, Wellington, 6242 New Zealand; 39 Institute for Biomedicine, Eurac Research, Affiliated Institute of the University of Lübeck, Bolzano, 39100 Italy; 40 MRC-PHE Centre for Environment and Health, Imperial College London, London, W2 1PG UK; 41 INSERM UMR_S 1166, Sorbonne Universités, Paris, 75013 France; 42 ICAN Institute for Cardiometabolism and Nutrition, Paris, 75013 France; 43 QIMR Berghofer Institute of Medical Research, Brisbane, Australia; 44 Department of Clinical Epidemiology, Leiden University Medical Center, Leiden, 2333 ZA The Netherlands; 45 Centre for Cognitive Ageing and Cognitive Epidemiology, University of Edinburgh, Edinburgh, EH8 9JZ UK; 46 Centre for Genomic & Experimental Medicine, Institute of Genetics & Molecular Medicine, University of Edinburgh, Edinburgh, EH4 2XU UK; 47 Clinical Division of Neurogeriatrics, Department of Neurology, Medical University of Graz, Graz, 8036 Austria; 48 Institute of Medical Informatics, Statistics and Documentation, Medical University of Graz, Graz, 8036 Austria; 49 CONACyT, Instituto Nacional de Ciencias Médicas y Nutrición Salvador Zubirán, Mexico, 03940 México; 50 Department of Human Genetics, University of Chicago, Chicago, IL 60637 USA; 51 Department of Complex Trait Genetics, Center for Neurogenomics and Cognitive Research, Vrije Universiteit Amsterdam, Amsterdam, 1081 HV The Netherlands; 52 Alzheimer Center Department of Neurology, VU University Medical Center, Amsterdam Neuroscience, Amsterdam, 1081HV The Netherlands; 53 Interfaculty Institute for Genetics and Functional Genomics, University Medicine Greifswald, Greifswald, 17475 Germany; 54 Chair of Experimental Bioinformatics, TUM School of Life Sciences Weihenstephan, Technical University of Munich, Freising-Weihenstephan, 85354 Germany; 55 Department of Immunology, Genetics and Pathology, Science for Life Laboratory, Uppsala University, 75108 Uppsala, Sweden; 56 Vth Department of Medicine (Nephrology, Hypertensiology, Rheumatology, Endocrinology, Diabetology), Medical Faculty Mannheim, Heidelberg University, Mannheim, 68167 Germany; 57 Cancer Genomics Research Laboratory, Leidos Biomedical Research, Inc., Frederick National Lab for Cancer Research, Frederick, MD USA; 58 Wellcome Centre for Human Genetics, University of Oxford, Oxford, OX3 7BN UK; 59 Department of Computational Biology and Medical Sciences, Graduate school of Frontier Sciences, The University of Tokyo, Tokyo, 108-8639 Japan; 60 Department of Molecular Epidemiology, German Institute of Human Nutrition Potsdam-Rehbruecke, Nuthetal, Germany; 61 German Center for Diabetes Research (DZD), München-Neuherberg, Germany; 62 Medical Research Institute, Ninewells Hospital and School of Medicine, University of Dundee, Dundee, UK; 63 Division of Endocrinology, Diabetes and Nutrition, Department of Medicine, University of Maryland, School of Medicine, Baltimore, MD 21201 USA; 64 Program for Personalized and Genomic Medicine, Department of Medicine, University of Maryland, School of Medicine, Baltimore, MD 21201 USA; 65 Department of Epidemiology, University of Groningen, University Medical Center Groningen, Groningen, 9700 RB The Netherlands; 66 Institute for Cardiogenetics, University of Lübeck, Lübeck, 23562 Germany; 67 DZHK (German Research Centre for Cardiovascular Research), partner site Hamburg/Lübeck/Kiel, Lübeck, 23562 Germany; 68 University Heart Center Luebeck, Lübeck, 23562 Germany; 69 Charité – University Medicine Berlin, corporate member of Freie Universität Berlin, Humboldt-Universität zu Berlin, and Berlin Institute of Health, Institute for Dental and Craniofacial Sciences, Department of Periodontology and Synoptic Dentistry, Berlin, Germany; 70 Institute of Genetics and Biophysics A. Buzzati-Traverso - CNR, Naples, 80131 Italy; 71 Finnish Institute for Molecular Medicine, University of Helsinki, Helsinki, Finland; 72 Genomics and Molecular Medicine Unit, CSIR-Institute of Genomics and Integrative Biology, New Delhi, 110020 India; 73 Department of Clinical Sciences, Diabetes and Endocrinology, Lund University Diabetes Centre, Lund University, Skåne University Hospital, Malmö, 20502 Sweden; 74 Genomic Research on Complex diseases (GRC Group), CSIR-Centre for Cellular and Molecular Biology, Hyderabad, Telangana 500007 India; 75 ePhood Scientific Unit, ePhood SRL, Bresso (Milano), 20091 Italy; 76 Department of Health Sciences, University of Milano, Milano, 20139 Italy; 77 Neuroalgology Unit, IRCCS Foundation Carlo Besta Neurological Institute, Milano, 20133 Italy; 78 Department of Pediatrics, College of Medicine, University of Oklahoma Health Sciences Center, Oklahoma City, OK 73104 USA; 79 Center for Research on Genomics and Global Health, National Human Genome Research Institute, National Institutes of Health, Bethesda, Maryland 20892-5635 USA; 80 Department of Epidemiology Research, Statens Serum Institut, Copenhagen, DK-2300 Denmark; 81 Icelandic Heart Association, Kopavogur, 201 Iceland; 82 Faculty of Medicine, School of Health Sciences, University of Iceland, Reykjavik, 101 Iceland; 83 Department of Genetics, University of North Carolina, Chapel Hill, NC 27599 USA; 84 Institute of Health and Wellbeing, University of Glasgow, Glasgow, G12 8RZ UK; 85 Cardiovascular Medicine Unit, Department of Medicine Solna, Centre for Molecular Medicine, Karolinska Institutet, Stockholm, 171 76 Sweden; 86 Laboratory of Epidemiology and Population Sciences, National Institute on Aging, National Institutes of Health, Baltimore City, Maryland 21224 USA; 87 Department of Cardiology, Leiden University Medical Center, Leiden, 2300 RC the Netherlands; 88 Section of Gerontology and Geriatrics, Department of Internal Medicine, Leiden University Medical Center, Leiden, 2300RC the Netherlands; 89 Department of Epidemiology, Harvard T.H. Chan School of Public Health, Boston, MA 02115 USA; 90 Program in Genetic Epidemiology and Statistical Genetics, Harvard T.H. Chan School of Public Health, Boston, MA 02115 USA; 91 University of Groningen, University Medical Center Groningen, Department of Cardiology, Ther Netherlands, Groningen, 9713 GZ the Netherlands; 92 Italian Institute for Genomic Medicine (IIGM) and Dept. Medical Sciences, University of Turin, Italy, Turin, 10126 Italy; 93 Division of Statistical Genomics, Department of Genetics, Washington University School of Medicine, Saint Louis, MO 63110-1093 USA; 94 NIHR Barts Cardiovascular Biomedical Research Centre, Barts and The London School of Medicine and Dentistry, Queen Mary University of London, London, EC1M 6BQ UK; 95 Department of Clinical Pharmacology, William Harvey Research Institute, Barts and The London School of Medicine and Dentistry, Queen Mary University of London, London, EC1M 6BQ UK; 96 MRC Integrative Epidemiology Unit at the University of Bristol, Bristol, BS8 2BN UK; 97 School of Psychological Science, University of Bristol, Bristol, BS8 1TU UK; 98 Department of Medicine, GeneSTAR Research Program, Johns Hopkins University School of Medicine, Baltimore, MD 21287 USA; 99 Department of Genetic Medicine, Weill Cornell Medicine Qatar, Doha, Qatar; 100 Computer and Systems Engineering, Alexandria University, Alexandria, Egypt; 101 Department of Epidemiology, School of Public Health, University of Michigan, Ann Arbor, MI 48109 USA; 102 Departamento de Endocrinología y Metabolismo, Instituto Nacional de Ciencias Médicas y Nutrición Salvador Zubirán, Mexico, 14080 México; 103 Unidad de Investigacion de Enfermades Metabolicas, Tecnologico de Monterrey, Escuela de Medicina y Ciencias de la Salud, Monterrey, N.L. 64710 México; 104 Department of Ophthalmology, Graduate School of Medical Sciences, Kyushu University, Fukuoka, Fukuoka, 812-8582 Japan; 105 Immunobiology of Dendritic Cells, Institut Pasteur, Paris, 75015 France; 106 Inserm U1223, Paris, 75015 France; 107 Centre for Translational Research, Institut Pasteur, Paris, 75015 France; 108 Department of Cancer Immunology, Genentech Inc, San Francisco, California 94080 USA; 109 Division of Preventive Medicine, Department of Family Medicine and Public Health, UC San Diego School of Medicine, La Jolla, California 92093 USA; 110 Estonian Genome Center, University of Tartu, University of Tartu, Tartu, 51010 Estonia; 111 Singapore Eye Research Institute, Singapore National Eye Centre, Singapore, Singapore, 169856 Singapore; 112 Ophthalmology & Visual Sciences Academic Clinical Program (Eye ACP), Duke-NUS Medical School, Singapore, Singapore, 169857 Singapore; 113 Department of Ophthalmology, Yong Loo Lin School of Medicine, National University of Singapore, Singapore, Singapore, 119228 SG Singapore; 114 Endocrine Research Center, Research Institute for Endocrine Sciences, Shahid Beheshti University of Medical Sciences, Tehran, 19839-63113 Iran; 115 Department of Epidemiology, German Institute of Human Nutrition Potsdam-Rehbruecke, Nuthetal, Germany; 116 Health Science Center at Houston, UTHealth School of Public Health, University of Texas, Houston, TX 77030 USA; 117 USC-Office of Population Studies Foundation, Inc., Department of Nutrition and Dietetics, Talamban, University of San Carlos, Cebu City, 6000 Cebu Philippines; 118 Department of Vascular Surgery, Division of Surgical Specialties, University Medical Center Utrecht, University of Utrecht, Utrecht, Utrecht, 3584 CX Netherlands; 119 Digital Health Center, Hasso Plattner Institute, Universität Potsdam, Potsdam, 14482 Germany; 120 Division of Cancer Epidemiology & Genetics, National Cancer Institute, National Institutes of Health, Bethesda, MD 20892 USA; 121 Institute of Population Health Sciences, National Health Research Institutes, Miaoli, Taiwan Taiwan; 122 Institute for Maternal and Child Health - IRCCS Burlo Garofolo, Trieste, 34137 Italy; 123 National Human Genome Research Institute, National Institutes of Health, Bethesda, Maryland 20892 USA; 124 Department of Psychology, University of Edinburgh, 7 George Square, Edinburgh, EH8 9JZ UK; 125 Department of Medicine, Yong Loo Lin School of Medicine, National University of Singapore, Singapore, 119228 SG Singapore; 126 Department of Nutrition, Harvard T.H. Chan School of Public Health, Boston, Massachusetts 02115 USA; 127 Cellular and Molecular Endocrine Research Center, Research Institute for Endocrine Sciences, Shahid Beheshti University of Medical Sciences, Tehran, 19839-63113 Iran; 128 Department of Internal Medicine, Rush University Medical Center, Chicago, Illinois USA; 129 MEMO Research, Molecular and Clinical Medicine, University of Dundee, Dundee, DD19SY UK; 130 Department of Internal Medicine B, University Medicine Greifswald, Greifswald, 17475 Germany; 131 DZHK (German Centre for Cardiovascular Research), partner site Greifswald, Greifswald, 17475 Germany; 132 Department of Genetics, King Faisal Specialist Hospital and Research Center, Riyadh, KSA 12713 Saudi Arabia; 133 Department of Anthropology, University of Iceland, Reykjavik, 101 Iceland; 134 National Institute for Health Research Imperial Biomedical Research Centre, Imperial College Healthcare NHS Trust and Imperial College London, London, UK; 135 UK Dementia Research Institute (UK DRI) at Imperial College London, London, UK; 136 Health Data Research UK - London, London, England; 137 Department of Pediatrics, Erasmus University Medical Center, Rotterdam, 3015CN The Netherlands; 138 Section on Nephrology, Department of Internal Medicine, Wake Forest School of Medicine, Winston-Salem, NC 27101 US; 139 Medical Genetics, Institute for Maternal and Child Health - IRCCS Burlo Garofolo, Trieste, Italy; 140 Division of Cardiovascular Medicine, Radcliffe Department of Medicine, University of Oxford, Oxford, OX3 9DU UK; 141 Division of Endocrinology, Diabetes, and Metabolism, Department of Medicine, Cedars-Sinai Medical Center, Los Angeles, California 90048 USA; 142 Braun School of Public Health, Hebrew University-Hadassah Medical Center, Jerusalem, Israel; 143 School of Engineering and Natural Sciences, University of Iceland, Reykjavik, 101 Iceland; 144 Chinese Academy of Medical Sciences, Beijing, 100730 China; 145 Social, Genetic and Developmental Psychiatry Centre, Institute of Psychiatry, Psychology & Neuroscience, King’s College London, London, SE5 8AF UK; 146 Department of Preventive Medicine, Keck School of Medicine, University of Southern California, Los Angeles, California 90089 USA; 147 Blizard Institute, Queen Mary University of London, London, E1 2AT UK; 148 Laboratory of Genome Technology, Institute of Medical Science, The University of Tokyo, Tokyo, 108-8639 Japan; 149 Laboratory of Clinical Chemistry and Hematology, Division Laboratories and Pharmacy, University Medical Center Utrecht, University of Utrecht, Utrecht, Utrecht, 3584 CX Netherlands; 150 Shanghai Institute of Hematology, State Key Laboratory Of Medical Genomics, Rui-jin Hospital, Shanghai Jiao Tong University School of Medicine, Shanghai, China 200025 China; 151 Division of Endocrine and Metabolism, Tri-Service General Hospital Songshan branch, Taipei, Taiwan Taiwan; 152 School of Medicine, National Defense Medical Center, Taipei, Taiwan Taiwan; 153 Unit of Public Health Promotion, National Institute for Health and Welfare, Helsinki, Finland; 154 Center for Genomic Medicine, Kyoto University Graduate School of Medicine, Kyoto, 606-8507 Japan; 155 Department of Biomedical Informatics, Harvard Medical School, Boston, MA 02115 USA; 156 Deutsches Herzzentrum München, Klinik für Herz- und Kreislauferkrankungen, Technische Universität München, Munich, 80636 Germany; 157 Department of Public Health and Primary Care, University of Cambridge, Cambridge, CB2 0SR UK; 158 Human Genetics, Genome Institute of Singapore, Agency for Science, Technology and Research, Singapore, Singapore, 138672 Singapore; 159 Health Services and Systems Research, Duke-NUS Medical School, Singapore, Singapore 169857; 160 Centre for Global Health, Faculty of Medicine, University of Split, Split, Croatia; 161 Institute of Clinical Medicine, Internal Medicine, University of Eastern Finland, Kuopio, Finland; 162 Kuopio University Hospital, Kuopio, Finland; 163 Bristol NIHR Biomedical Research Centre, Bristol, BS8 2BN UK; 164 Population Health Science, Bristol Medical School, Bristol, BS8 2BY UK; 165 Division of Endocrinology and Metabolism, Department of Internal Medicine, Taichung Veterans General Hospital, Taichung, Taiwan Taiwan; 166 School of Medicine, National Yang-Ming University, Taipei, Taiwan, Taipei, 112 Taiwan; 167 School of Medicine, Chung Shan Medical University, Taichung, Taiwan, Taichung City, 402 Taiwan; 168 Department of Medical Research, Taichung Veterans General Hospital, Taichung, Taiwan Taiwan; 169 Department of Internal Medicine A, University Medicine Greifswald, Greifswald, 17475 Germany; 170 Department of Epidemiology and Biostatistics, Peking University Health Science Centre, Peking University, Beijing, 100191 China; 171 Translational Laboratory in Genetic Medicine, Agency for Science, Technology and Research, Singapore (A*STAR), Singapore, 138648 Singapore; 172 Department of Biochemistry, Yong Loo Lin School of Medicine, National University of Singapore, Singapore, Singapore, 117596 Singapore; 173 National Institute of Environmental Health Sciences, National Institutes of Health, Department of Health and Human Services, Research Triangle Park, Durham, NC 27709 USA; 174 Department of Epidemiology, Johns Hopkins Bloomberg School of Public Health, Baltimore, MD 21205 USA; 175 Center for Public Health Genomics, University of Virginia School of Medicine, Charlottesville, VA 22908 USA; 176 Genomics of Renal Diseases and Hypertension Unit, IRCCS San Raffaele Scientific Institute, Università Vita Salute San Raffaele, Milano, 20132 Italy; 177 Helmholtz Zentrum München, Independent Research Group Clinical Epidemiology, Neuherberg, 85764 Germany; 178 Institute of Human Genetics, Helmholtz Zentrum Muenchen, Neuherberg, 85764 Germany; 179 Institute of Human Genetics, Technical University of Munich, Munich, 81675 Germany; 180 DZHK (German Center for Cardiovascular Research), partner site Munich Heart Alliance, Munich, 80802 Germany; 181 Estonian Genome Center, Institute of Genomics, University of Tartu, Tartu, 51010 Estonia; 182 Laboratory for Genotyping Development, RIKEN Center for Integrative Medical Sciences, Yokohama, Kanagawa 230-0045 Japan; 183 Laboratory of Haematology, La Timone Hospital, Marseille, France; 184 INSERM UMR_S 1263, Center for CardioVascular and Nutrition research (C2VN), Aix-Marseille University, Marseille, France; 185 Departamento de Economía, Universidad Autónoma Metropolitana, Mexico, 09340 México; 186 Medical School, The University of Western Australia, Perth, Western Australia/Australia 6009 Australia; 187 The University of Texas Health Science Center at Houston, School of Public Health, Department of Epidemiology, Human Genetics and Environmental Sciences, Houston, Texas 77030 USA; 188 Institute of Social and Preventive Medicine (ISPM), University of Bern, Bern, Switzerland; 189 Division of Molecular Pathology, Institute of Medical Science, The University of Tokyo, Tokyo, 108-8639 Japan; 190 The Institute of Medical Sciences, Aberdeen Biomedical Imaging Centre, University of Aberdeen, Aberdeen, AB25 2ZD UK; 191 Laboratory of Neurogenetics, Bethesda, MD 20892 USA; 192 Data Tecnica International LLC, Glen Echo, MD 20812 USA; 193 Institute of Clinical Chemistry and Laboratory Medicine, University Medicine Greifswald, Greifswald, 17475 Germany; 194 Oxford Centre for Diabetes, Endocrinology and Metabolism, University of Oxford, Headington, Oxford, OX3 7LJ UK; 195 Oxford NIHR Biomedical Research Centre, Oxford University Hospitals Trust, Oxford, UK; 196 Department of Paediatrics, University of Cambridge School of Clinical Medicine, Cambridge, CB2 0QQ UK; 197 Instituto Nacional de Medicina Genómica, Mexico, 14610 México; 198 Institute of Cardiovascular and Medical Sciences, University of Glasgow, Glasgow, G12 8TA UK; 199 Division of Epidemiology & Community Health, School of Public Health, University of Minnesota, Minneapolis, MN 55454 USA; 200 Gen-info Ltd, Zagreb, Croatia, Zagreb, Select a Province 10000 Croatia; 201 International Centre for Circulatory Health, Imperial College London, London, W2 1PG UK; 202 Imperial Clinical Trials Unit, Imperial College London, London, London, W12 7TA UK; 203 Human Evolutionary Genetics Unit, Institut Pasteur, Paris, 75015 France; 204 Centre National de la Recherche Scientifique (CNRS) UMR2000, Paris, 75015 France; 205 Center of Bioinformatics, Biostatistics and Integrative Biology, Institut Pasteur, Paris, 75015 France; 206 Department of Psychology and Logopedics, Faculty of Medicine, University of Helsinki, University of Helsinki, Helsinki, 00014 Finland; 207 Hero Heart Institute and Dyanand Medical College and Hospital, Ludhiana, Punjab India; 208 Division of Biostatistics, Washington University School of Medicine, St. Louis, Missouri USA; 209 Harvard Medical School, Boston, MA 02115 USA; 210 Department of Applied Economics, Erasmus School of Economics, Erasmus University Rotterdam, Rotterdam, 3062 PA The Netherlands; 211 Erasmus University Rotterdam Institute for Behavior and Biology, Erasmus University Rotterdam, Rotterdam, 3062 PA The Netherlands; 212 IRCCS Neuromed, Pozzilli (IS), 86077 Italy; 213 Gottfried Schatz Research Center (for Cell Signaling, Metabolism and Aging), Division of Molecular Biology and Biochemistry, Medical University of Graz, 8010 Graz, Austria; 214 Unit of Genomics of Complex Diseases, Institut de Recerca Hospital de la Santa Creu i Sant Pau, IIB-Sant Pau, Barcelona, Spain; 215 San Raffaele Research Institute, Milano, Italy; 216 Department of Public Health Solutions, National Institute for Health and Welfare, Helsinki, FI-00271 Finland; 217 Department of Biostatistics, and Center for Statistical Genetics, University of Michigan, Ann Arbor, Michigan 48109 USA; 218 Dept of Cell and Molecular Biology, National Bioinformatics Infrastructure Sweden, Science for Life Laboratory, Uppsala University, Uppsala, SE-752 37 Uppsala, Sweden; 219 Department of Cardiology, Division Heart & Lungs, University Medical Center Utrecht, University of Utrecht, Utrecht, Utrecht, 3485 CX Netherlands; 220 School of Medicine, National Yang-Ming University, Taipei, Taiwan Taiwan; 221 Institute of Medical Technology, National Chung-Hsing University, Taichung, Taiwan Taiwan; 222 School of Basic and Applied Sciences, Dayananda Sagar University, Bangalore, Karnataka 560078 India; 223 Diabetes Unit, KEM Hospital and Research Centre, Pune, Maharashtra 411101 India; 224 Symbiosis Statistical Institute, Symbiosis International University, Pune, Maharashtra 411007 India; 225 Panjab University, Chandigarh, India; 226 Division of Population Health Sciences, Ninewells Hospital and Medical School, University of Dundee, Dundee, DD1 9SY UK; 227 RCSI Molecular & Cellular Therapeutics (MCT), Royal College of Surgeons in Ireland, RCSI Education & Research Centre, Beaumont Hospital, Dublin 9, Ireland; 228 Alzheimer Scotland Dementia Research Centre, University of Edinburgh, Edinburgh, EH8 9JZ Scotland; 229 School of Physiotherapy and Exercise Science, Faculty of Health Sciences, Curtin University, Perth, Western Australia/Australia 6102 Australia; 230 Department of Respiratory Medicine, Ghent University Hospital, Ghent, 9000 Belgium; 231 Department of Pathology, University of Vermont, Colchester, VT 05446 USA; 232 Departamento de Medicina Genómica y Toxicología Ambiental, Instituto de Investigaciones Biomédicas, UNAM, Mexico, 04510 México; 233 Unidad De Biología Molecular y Medicina Genómica, Instituto Nacional de Ciencias Médicas y Nutrición Salvador Zubirán, Mexico, 14080 México; 234 Department of Hygiene and Epidemiology, University of Ioannina Medical School, Ioannina, 45110 Greece; 235 Institute of Genetic and Biomedical Research - Support Unity, National Research Council of Italy, Rome, Italy; 236 MRC Human Genetics Unit, Institute of Genetics and Molecular Medicine, University of Edinburgh, Edinburgh, EH4 2XU Scotland; 237 Institute for Molecular Medicine Finland (FIMM), University of Helsinki, Helsinki, FI-00014 Finland; 238 Department of Cardiology, Hero DMC Heart Institute, Dayanand Medical College & Hospital, Ludhiana, Punjab 141001 India; 239 Beijing Institute of Ophthalmology, Beijing Tongren Eye Center, Beijing Tongren Hospital, Capital Medical University, Beijing Ophthalmology and Visual Science Key Lab, Beijing, China 100005 China; 240 Centre for Population Health Sciences, Usher Institute of Population Health and Informatics, University of Edinburgh, Edinburgh, EH8 9AG Scotland; 241 Diabetes Unit, K.E.M. Hospital Research Centre, Pune, MAH 411011 India; 242 Department of Epidemiology, Graduate School of Public Health, University of Pittsburgh, Pittsburgh, Pennsylvania USA; 243 Department of Cardiology, Division Heart & Lungs, University Medical Center Utrecht, University of Utrecht, Utrecht, Utrecht, 3584 CX Netherlands; 244 Institute of Cardiovascular Science, Faculty of Population Health Sciences, University College London, London, WC1E 6DD UK; 245 Durrer Center for Cardiovascular Research, Netherlands Heart Institute, Utrecht, Netherlands; 246 Farr Institute of Health Informatics Research and Institute of Health Informatics, University College London, London, UK; 247 Department of Internal Medicine, University Medical Center Groningen, University of Groningen, Groningen, 9713GZ The Netherlands; 248 Rush Alzheimer’s Disease Center, Rush University Medical Center, Chicago, IL 60612 USA; 249 Department of Neurological Sciences, Rush University Medical Center, Chicago, IL 60612 USA; 250 Systems Genomics Laboratory, School of Biotechnology, Jawaharlal Nehru University, New Delhi, 110067 India; 251 Pennington Biomedical Research Center, Baton Rouge, Louisiane 70808 USA; 252 Center for Applied Genomics, Division of Human Genetics, Children’s Hospital of Philadelphia, Philadelphia, PA 19104 USA; 253 Quantinuum Research LLC, San Diego, CA 92101 USA; 254 Cardiovascular Health Research Unit, Department of Medicine, University of Washington, Seattle, WA 98101 USA; 255 Center for Experimental Social Science, Department of Economics, New York University, New York, New York, 10012 USA; 256 Research Institute for Industrial Economics (IFN), Stockholm, 102 15 Sweden; 257 Lee Kong Chian School of Medicine, Nanyang Technological University, Singapore, 308232 Singapore; 258 Imperial College Healthcare NHS Trust, London, London, W12 0HS UK; 259 Department of Preventive Medicine, Northwestern University Feinberg School of Medicine, Chicago, IL 60611 USA; 260 Institute of Biomedical Technologies Milano, National Research Council of Italy (CNR), Segrate (Milano), 20090 Italy; 261 Bio4Dreams Scientific Unit, Bio4Dreams SRL, Bio4Dreams - business nursery for life sciences, Milano, 20121 Italy; 262 Institute of Clinical Molecular Biology, Christian-Albrechts-University of Kiel, 24105 Kiel, Germany; 263 Department of General Practice and Primary health Care, University of Helsinki, Tukholmankatu 8 B, Helsinki, 00014 Finland; 264 National Institute for Health and Welfare, Helsinki, Finland; 265 Unit of General Practice, Helsinki University Central Hospital, Helsinki, Finland; 266 Folkhälsan Research Centre, Helsinki, Finland; 267 Vasa Central Hospital, Vaasa, Finland; 268 Survey Research Center, Institute for Social Research, University of Michigan, Ann Arbor, MI 48014 USA; 269 Montreal Heart Institute, Montreal, QC Canada; 270 Centro de Estudios en Diabetes, Unidad de Investigacion en Diabetes y Riesgo Cardiovascular, Centro de Investigacion en Salud Poblacional, Instituto Nacional de Salud Publica, Cuernavaca, 01120 México; 271 Department of Pediatrics, Perelman School of Medicine, University of Pennsylvania, Philadelphia, PA 19104 USA; 272 Cardiovascular Medicine Unit, Department of Medicine Solna, Centre for Molecular Medicine, Stockholm, 171 76 Sweden; 273 Department of Paediatrics, Yong Loo Lin School of Medicine, National University of Singapore, Singapore, Singapore; 274 Khoo Teck Puat - National University Children’s Medical Institute, National University Health System, Singapore, Singapore; 275 Research Unit of Internal Medicine, Medical Research Center Oulu, University of Oulu and Oulu University Hospital, Oulu, 90014 Finland; 276 Division of Epidemiology, Department of Internal Medicine, University of Utah School of Medicine, Salt Lake City, Utah 84108 USA; 277 Center for Translational & Computational Neuroimmunology, Department of Neurology, Columbia University Medical Center, 650 West 168th street, PH19-311, Newyork, NY 10032 USA; 278 Cell Circuits Program, Broad Institute, Cambridge, MA 02142 USA; 279 Department of Economics, Stockholm School of Economics, Stockholm, SE-113 83 Sweden; 280 Department of Ophthalmology, Medical Faculty Mannheim of the Ruprecht-Karls-University of Heidelberg, Mannheim, 698167 Germany; 281 Department of Public Health, University of Helsinki, Helsinki, FI-00014 Finland; 282 Oxford NIHR Biomedical Research Centre, Oxford University Hospitals NHS Foundation Trust, John Radcliffe Hospital, Oxford, OX3 9DU UK; 283 Laboratory of Clinical Chemistry and Hematology, Division Laboratories and Pharmacy, University Medical Center Utrecht, University of Utrecht, Utrecht, 3584 CX Netherlands; 284 Helsinki Collegium for Advanced Studies, University of Helsinki, University of Helsinki, Helsinki, 00014 Finland; 285 University Hospital Schleswig-Holstein (UKSH), Campus Kiel, Kiel, 24105 Germany; 286 Institute of Epidemiology and PopGen Biobank, University of Kiel, Kiel, Schleswig Holstein 24105 Germany; 287 Department of Statistics, University of Auckland, Auckland, New Zealand; 288 Clinical Institute of Medical and Chemical Laboratory Diagnostics, Medical University of Graz, Graz, Austria; 289 Synlab Academy, Synlab Holding Deutschland GmbH, Mannheim, Germany; 290 Department of Genetics, King Faisal Specialist Hospital and Research Centre, Riyadh, 11211 Saudi Arabia; 291 Institute for Molecular Bioscience, The University of Queensland, Brisbane, Queensland 4072 Australia; 292 Department of Public Health and Primary Care, Leiden University Medical Center, Leiden, 2333 ZA The Netherlands; 293 School of Biomedical Sciences, Institute of Health and Biomedical Innovation, Queensland University of Technology, Kelvin Grove, QLD 4059 Australia; 294 Department of Psychiatry, Interdisciplinary Center Psychopathology and Emotion Regulation, University of Groningen, University Medical Center Groningen, Groningen, 9700 RB The Netherlands; 295 Department of Medicine, Columbia University Medical Center, New York, New York USA; 296 Pat Macpherson Centre for Pharmacogenetics and Pharmacogenomics, The School of Medicine, University of Dundee, Dundee, DD1 9SY UK; 297 School of Medicine and Public Health, Faculty of Medicine and Health, The University of Newcastle, Newcastle, New South Wales Australia; 298 Division of Obstetrics and Gynaecology, The University of Western Australia, Perth, Western Australia/Australia 6009 Australia; 299 Department of kinesiology, Laval University, Quebec, QC G1V 0A6 Canada; 300 Institute of Nutrition and Functional Foods, Laval University, Quebec, QC G1V 0A6 Canada; 301 Institute of Genetic and Biomedical Research - Support Unity, National Research Council of Italy, Sassari, 07100 Italy; 302 Department of Clinical Genetics, Amsterdam Neuroscience, VU Medical Centre, Amsterdam, 1081HV The Netherlands; 303 Cardiovascular Health Research Unit, Departments of Epidemiology, Medicine and Health Services, University of Washington, Seattle, WA 98101 USA; 304 Kaiser Permanente Washington Health Research Institute, Seattle, WA 98101 USA; 305 Department of Medicine, Faculty of Medicine, Université de Montréal, Montreal, Quebec H3T 1J4 Canada; 306 Laboratory of Experimental Cardiology, Division Heart & Lungs, University Medical Center Utrecht, University of Utrecht, Utrecht, Utrecht, 3584 CX Netherlands; 307 Oklahoma Center for Neuroscience, Oklahoma City, OK 73104 USA; 308 Institute of Nutritional Sciences, University of Potsdam, Nuthetal, Germany; 309 Deutsches Zentrum für Herz- und Kreislauferkrankungen (DZHK), Munich Heart Alliance, Munich, 80636 Germany; 310 Regeneron Genetics Center, Regeneron Pharmaceuticals, Inc, Tarrytown, NY 10591-6607 USA; 311 Faculty of Health Studies, University of Bradford, Bradford, West Yorkshire BD7 1DP UK; 312 Cardiovascular Health Research Unit, Division of Cardiology, University of Washington, Seattle, WA 98101 USA; 313 Duke-NUS Medical School, National University of Singapore, Singapore, Singapore, 169857 SG Singapore; 314 Institute for Community Medicine, University Medicine Greifswald, Greifswald, 17475 Germany; 315 Department of Population Health Sciences, Bristol Medical School, University of Bristol, Bristol, BS8 2PR UK; 316 Avon Longitudinal Study of Parents and Children (ALSPAC), University of Bristol, Bristol, BS8 2PR UK; 317 Endocrinology, Abdominal Centre, University of Helsinki, Helsinki University Hospital, Helsinki, Finland; 318 Folkhalsan Research Center, Helsinki, Finland; 319 Research Program of Diabetes and Endocrinology, University of Helsinki, Helsinki, Finland; 320 Department of Medicine, Internal Medicine, Lausanne University Hospital, Lausanne, 1011 Switzerland; 321 Division of Gastroenterology, Hepatology and Nutrition, Children’s Hospital of Philadelphia, Philadelphia, PA 19146 USA; 322 Unit of Genomics and Biomarkers, National Institute for Health and Welfare, Helsinki, 00271 Finland; 323 National Heart and Lung Institute, Imperial College London, London, W12 0NN UK; 324 The Mindich Child Health and Development Institute, The Icahn School of Medicine at Mount Sinai, New York, NY 10029 USA; 325 Roslin Institute and Royal (Dick) School of Veterinary Studies, University of Edinburgh, Easter Bush, Midlothian, EH25 9RG Scotland; 326 Program in Medical and Population Genetics, Broad Institute, Broad Institute, Cambridge, MASSACHUSETTS 02142 USA

**Keywords:** Consanguinity, Genetic association study, Genetic markers, Inbreeding

## Abstract

In many species, the offspring of related parents suffer reduced reproductive success, a phenomenon known as inbreeding depression. In humans, the importance of this effect has remained unclear, partly because reproduction between close relatives is both rare and frequently associated with confounding social factors. Here, using genomic inbreeding coefficients (*F*_ROH_) for >1.4 million individuals, we show that *F*_ROH_ is significantly associated (*p* < 0.0005) with apparently deleterious changes in 32 out of 100 traits analysed. These changes are associated with runs of homozygosity (ROH), but not with common variant homozygosity, suggesting that genetic variants associated with inbreeding depression are predominantly rare. The effect on fertility is striking: *F*_ROH_ equivalent to the offspring of first cousins is associated with a 55% decrease [95% CI 44–66%] in the odds of having children. Finally, the effects of *F*_ROH_ are confirmed within full-sibling pairs, where the variation in *F*_ROH_ is independent of all environmental confounding.

## Abstract (web summary)

Inbreeding depression has been observed in many different species, but in humans a systematic analysis has been difficult so far. Here, analysing more than 1.3 million individuals, the authors show that a genomic inbreeding coefficient (FROH) is associated with disadvantageous outcomes in 32 out of 100 traits tested.

## Introduction

Given the pervasive impact of purifying selection on all populations, it is expected that genetic variants with large deleterious effects on evolutionary fitness will be both rare and recessive^1^. However, precisely because they are rare, most of these variants have yet to be identified and their recessive impact on the global burden of disease is poorly understood. This is of particular importance for the nearly one billion people living in populations where consanguineous marriages are common^2^, and the burden of genetic disease is thought to be disproportionately due to increased homozygosity of rare, recessive variants^3–5^. Although individual recessive variants are difficult to identify, the net directional effect of all recessive variants on phenotypes can be quantified by studying the effect of inbreeding^6^, which gives rise to autozygosity (homozygosity due to inheritance of an allele identical-by-descent).

Levels of autozygosity are low in most of the cohorts with genome-wide data^7,8^ and consequently very large samples are required to study the phenotypic impact of inbreeding^9^. Here, we meta-analyse results from 119 independent cohorts to quantify the effect of inbreeding on 45 commonly measured complex traits of biomedical or evolutionary importance, and supplement these with analysis of 55 more rarely measured traits included in UK Biobank^10^.

**Fig. 1:**
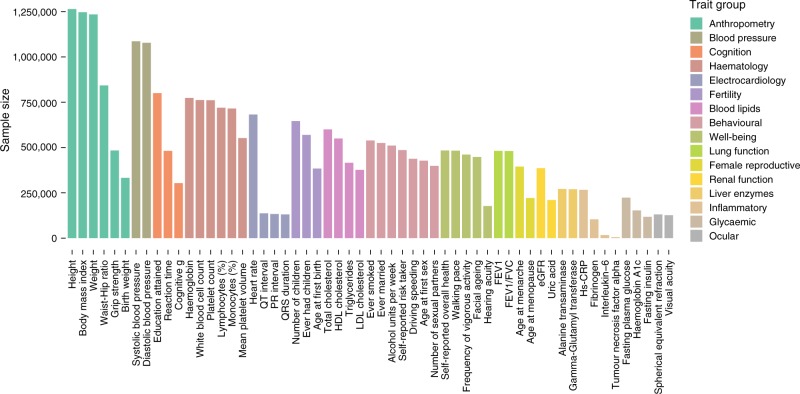
Census of complex traits. Sample sizes are given for analyses of 57 representative phenotypes, arranged into 16 groups covering major organ systems and disease risk factors. HDL high-density lipoprotein, LDL low-density lipoprotein, hs-CRP high-sensitivity C-reactive protein, TNF-alpha tumour necrosis factor alpha, FEV1 forced expiratory volume in one second, FVC forced vital capacity, eGFR estimated glomerular filtration rate

Continuous segments of homozygous alleles, or runs of homozygosity (ROH), arise when identical-by-descent haplotypes are inherited down both sides of a family. The fraction of each autosomal genome in ROH > 1.5 Mb (*F*_ROH_) correlates well with pedigree-based estimates of inbreeding^11^.We estimate *F*_ROH_ using standard methods and software^6,12^ for a total of 1,401,776 individuals in 234 uniform sub-cohorts. The traits measured in each cohort vary according to original study purpose, but together cover a comprehensive range of human phenotypes (Fig. 1, Supplementary Data 7). The five most frequently contributed traits (height, weight, body mass index, systolic and diastolic blood pressure) are measured in >1,000,000 individuals; a further 16 traits are measured >500,000 times.

We find that *F*_ROH_ is significantly associated with apparently deleterious changes in 32 out of 100 traits analysed. Increased *F*_ROH_ is associated with reduced reproductive success (decreased number and likelihood of having children, older age at first sex and first birth, decreased number of sexual partners), as well as reduced risk-taking behaviour (alcohol intake, ever-smoked, self-reported risk taking) and increased disease risk (self-reported overall health and risk factors including grip strength and heart rate). We show that the observed effects are predominantly associated with rare (not common) variants and, for a subset of traits, differ between men and women. Finally, we introduce a within-siblings method, which confirms that social confounding of *F*_ROH_ is modest for most traits. We therefore conclude that inbreeding depression influences a broad range of human phenotypes through the action of rare, recessive variants.

## Results

### Cohort characteristics

**Fig. 2:**
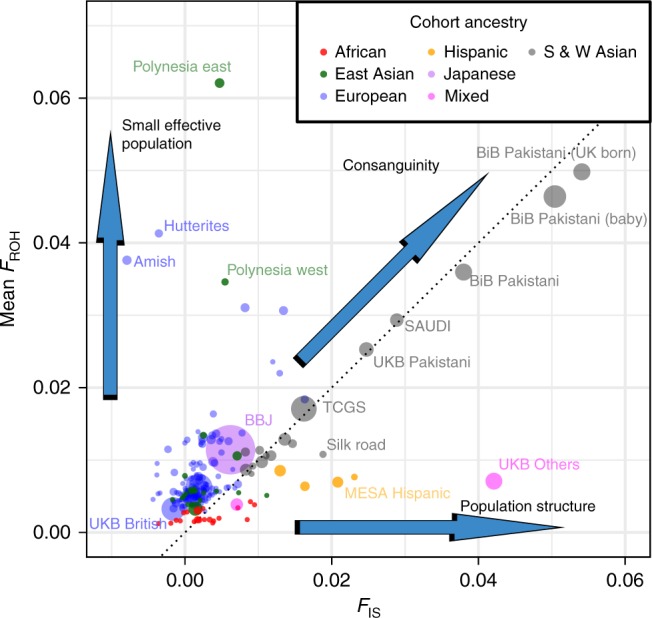
Mean *F*_ROH_ and *F*_IS_ for 234 ROHgen sub-cohorts. Each cohort is represented by a circle whose area is proportional to the approximate statistical power ($N\sigma _{F_{{\mathrm{ROH}}}}^2$) contributed to estimates of $\beta _{F_{{\mathrm{ROH}}}}$. Mean *F*_ROH_ can be considered as an estimate of total inbreeding relative to an unknown base generation, approximately tens of generations past. *F*_IS_ measures inbreeding in the current generation, with *F*_IS_ = 0 indicating random mating, *F*_IS_ > 0 indicating consanguinity, and *F*_IS_ < 0 inbreeding avoidance^46^. In cohorts along the *y*-axis, such as the Polynesians and the Anabaptist isolates, autozygosity is primarily caused by small effective population size rather than preferential consanguineous unions. In contrast, in cohorts along the dotted unity line, all excess SNP homozygosity is accounted for by ROH, as expected of consanguinity within a large effective population. A small number of cohorts along the *x*-axis, such as Hispanic and mixed-race groups, show excess SNP homozygosity without elevated mean *F*_ROH_, indicating population genetic structuring, caused for instance by admixture and known as the Wahlund effect. A few notable cohorts are labelled. BBJ Biobank Japan, BiB Born in Bradford, UKB UK Biobank, MESA Multiethnic Study of Atherosclerosis, TCGS Tehran Cardiometabolic Genetic Study

As expected, cohorts with different demographic histories varied widely in mean *F*_ROH_. The within-cohort standard deviation of *F*_ROH_ is strongly correlated with the mean (Pearson’s *r* = 0.82; Supplementary Fig. 3), and the most homozygous cohorts provide up to 100 times greater per-sample statistical power than cosmopolitan European-ancestry cohorts (Supplementary Data 5). To categorise cohorts, we plotted mean *F*_ROH_ against *F*_IS_ (Fig. 2). *F*_IS_ measures inbreeding as reflected by non-random mating in the most recent generation, and is calculated as the mean individual departure from Hardy–Weinberg equilibrium (*F*_SNP_; see Methods). Cohorts with high rates of consanguinity lie near the *F*_ROH_ = *F*_IS_ line, since most excess SNP homozygosity is caused by ROH. In contrast, cohorts with small effective population sizes, such as the Amish and Hutterite isolates of North America, have high average *F*_ROH_, often despite avoidance of mating with known relatives, since identical-by-descent haplotypes are carried by many couples, due to a restricted number of possible ancestors.

### Traits affected by *F*_ROH_

**Fig. 3:**
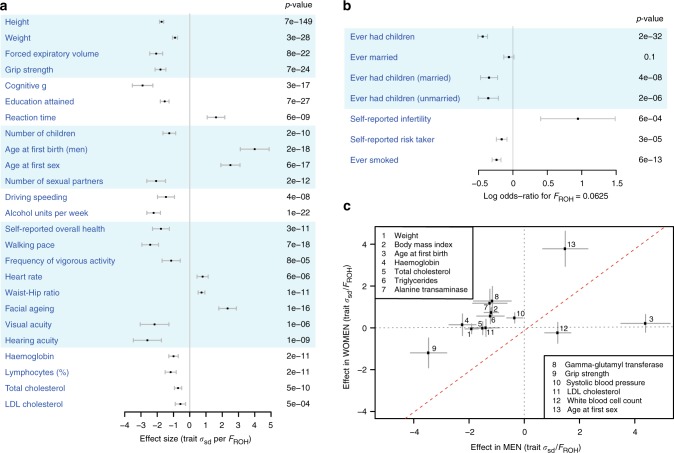
Scope of inbreeding depression. **a** Effect of *F*_ROH_ on 25 quantitative traits. To facilitate comparison between traits, effect estimates are presented in units of within-sex standard deviations. Traits shown here reached Bonferroni-corrected significance of *p* = 0.0005 (=0.05/100 traits). Sample sizes, within-sex standard deviations, and effect estimates in measurement units are shown in Supplementary Data 9. FEV1 forced expiratory volume in one second. Traits are grouped by type. **b** Effect of *F*_ROH_ on eight binary traits with associated *p* values. Effect estimates are reported as ln(Odds-Ratio) for the offspring of first cousins, for which *E*(*F*_ROH_) = 0.0625. Self-declared infertility is shown for information, although this trait does not reach Bonferroni corrected significant (${\mathrm{{OR}}}_{0.0625}$ = 2.6 ± 1.1, *p* *=* 0.0006). Numbers of cases and controls and effect estimates for all binary traits are shown in Supplementary Data 10. **c** Sex-specificity of ROH effects. The effect of *F*_ROH_ in men versus that in women is shown for 13 traits for which there was evidence of significant differences in the effects between sexes. For 11 of these 13 traits the magnitude of effect is greater in men than in women. Traits such as liver enzymes levels (alanine transaminase, gamma-glutamyl transferase) show sex-specific effects of opposite sign (positive in women, negative in men), which cancel out in the overall analysis. BMI body mass index, LDL low-density lipoprotein. All errors bars represent 95% confidence intervals

To estimate the effect of inbreeding on each of the 100 phenotypes studied, trait values were regressed on *F*_ROH_ within each cohort, taking account of covariates including age, sex, principal components of ancestry and, in family studies, a genomic relationship matrix (GRM) (Supplementary Data 3). Cross-cohort effect size estimates were then obtained by fixed-effect, inverse variance-weighted meta-analysis of the within-cohort estimates (Supplementary Data 10). Twenty-seven out of 79 quantitative traits and 5 out of 21 binary traits reach experiment-wise significance (0.05/100 or *p* < 0.0005; Fig. 3a, b). Among these are replications of the previously reported effects on reduction in height^13^, forced expiratory lung volume in one second, cognition and education attained^6^. We find that the 32 phenotypes affected by inbreeding can be grouped into five broader categories: reproductive success, risky behaviours, cognitive ability, body size, and health.

Despite the greater individual control over reproduction in the modern era, due to contraception and fertility treatments, we find that increased *F*_ROH_ has significant negative effects on five traits closely related to fertility. For example, an increase of 0.0625 in *F*_ROH_ (equivalent to the difference between the offspring of first cousins and those of unrelated parents) is associated with having 0.10 fewer children [*β*_0.0625_ = −0.10 ± 0.03 95% confidence interval (CI), *p* = 1.8 × 10^−10^]. This effect is due to increased *F*_ROH_ being associated with reduced odds of having any children (OR_0.0625_ = 0.65 ± 0.04, *p* = 1.7 × 10^−32^) as opposed to fewer children among parents (*β*_0.0625_ = 0.007 ± 0.03, *p* = 0.66). Since autozygosity also decreases the likelihood of having children in the subset of individuals who are, or have been, married, (OR_0.0625_ = 0.71 ± 0.09, *p* = 3.8 × 10^−8^) it appears that the cause is a reduced ability or desire to have children, rather than reduced opportunity. Consistent with this interpretation, we observe no significant effect on the likelihood of marriage (OR_0.0625_ = 0.94 ± 0.07, *p* = 0.12) (Fig. 3b). All effect size, odds ratios and 95% CI are stated as the difference between *F*_ROH_ = 0 and *F*_ROH_ = 0.0625.

The effects on fertility may be partly explained by the effect of *F*_ROH_ on a second group of traits, which capture risky or addictive behaviour. Increased *F*_ROH_ is associated with later age at first sex (*β*_0.0625_ = 0.83 ± 0.19 years, *p* *=* 5.8 × 10^−17^) and fewer sexual partners (*β*_0.0625_ = −1.38 ± 0.38, *p* *=* 2.0 × 10^−12^) but also reduced alcohol consumption (*β*_0.0625_ = −0.66 ± 0.12 units per week, *p* *=* 1.3 × 10^−22^), decreased likelihood of smoking (OR_0.0625_ = 0.79 ± 0.05, *p* *=* 5.9 × 10^−13^), and a lower probability of being a self-declared risk-taker (OR_0.0625_ = 0.84 ± 0.06, *p* *=* 3.4×10^−5^) or exceeding the speed limit on a motorway (*p* *=* 4.0 × 10^−8^). Conservative beliefs are likely to affect these traits, and are known to be confounded with *F*_ROH_ in some populations^14^, however, fitting religious participation as a covariate in UKB reduces, but does not eliminate the reported effects (Supplementary Fig. 10b, Supplementary Data 20). Similarly, fitting educational attainment as an additional covariate reduces 16 of 25 significant effect estimates, but actually increases 9, including age at first sex and number of children (Supplementary Fig. 10a, Supplementary Data 20). This is because reduced educational attainment is associated with earlier age at first sex and increased number of children, which makes it an unlikely confounder for the effects of *F*_ROH_, which are in the opposite directions.

A third group of traits relates to cognitive ability. As previously reported, increased autozygosity is associated with decreased general cognitive ability, *g*^6,15^ and reduced educational attainment^6^. Here, we also observe an increase in reaction time (*β*_0.0625_ = 11.6 ± 3.9 ms, *p* *=* 6.5 × 10^−9^), a correlate of general cognitive ability (Fig. 3a, Supplementary Data 10).

A fourth group relates to body size. We replicate previously reported decreases in height and forced expiratory volume^6^ (Supplementary Data 21) and we find that increased *F*_ROH_ is correlated with a reduction in weight (*β*_0.0625_ = 0.86 ± 0.12 kg, *p* *=* 3.4 × 10^−28^) and an increase in the waist to hip ratio (*β*_0.0625_ = 0.004 ± 0.001, *p* *=* 1.4 × 10^−11^).

The remaining effects are loosely related to health and frailty; higher *F*_ROH_ individuals report significantly lower overall health and slower walking pace, have reduced grip strength (*β*_0.0625_ = −1.24 ± 0.19 kg, *p* *=* 6.9 × 10^−24^), accelerated self-reported facial ageing, and poorer eyesight and hearing. Increased *F*_ROH_ is also associated with faster heart rate (*β*_0.0625_ = 0.56 ± 0.24 bpm, *p* *=* 5.9 × 10^−6^), lower haemoglobin (*β*_0.0625_ = 0.81 ± 0.24 gL^−1^, *p* *=* 1.6 × 10^−11^), lymphocyte percentage, and total cholesterol (*β*_0.0625_ = −0.05 ± 0.015 mmol L^−1^, *p* *=* 5.2 × 10^−10^).

### Sex-specific effects of *F*_ROH_

Intriguingly, for a minority of traits (13/100), the effect of *F*_ROH_ differs between men and women (Fig. 3c, Supplementary Data 12). For example, men who are the offspring of first cousins have 0.10 mmol L^−1^ [95% CI 0.08–0.12] lower total cholesterol on average, while there is no significant effect in women; LDL shows a similar pattern. More generally, for these traits, the effect in men is often of greater magnitude than the effect in women, perhaps reflecting differing relationships between phenotype and fitness.

### Associations most likely caused by rare, recessive variants

**Fig. 4:**
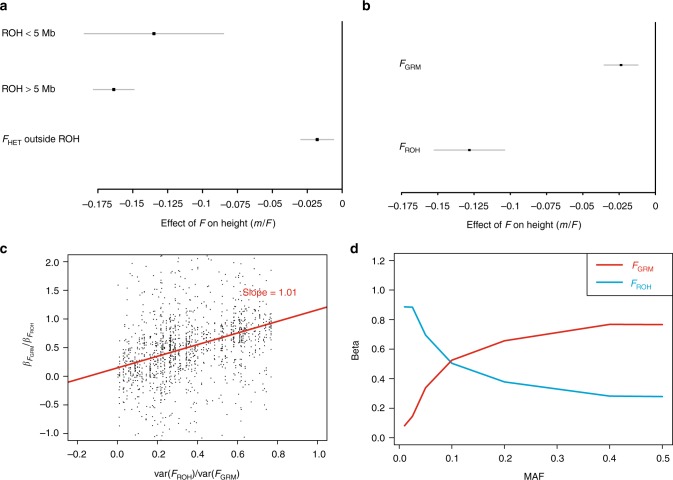
Inbreeding depression caused by ROH. **a** Effect of different ROH lengths on height, compared with the effect of SNP homozygosity outside of ROH. The effects of shorter (<5 Mb) and longer (>5 Mb) ROH per unit length are similar and strongly negative, whereas the effect of homozygosity outside ROH is much weaker. The pattern is similar for other traits (Supplementary Fig. 11a; Supplementary Data 14). **b** ${\boldsymbol{F}}_{{\mathbf{ROH}}}$ is more strongly associated than ${\boldsymbol{F}}_{{\mathbf{GRM}}}$ in a bivariate model of height. Meta-analysed effect estimates, and 95% confidence intervals, are shown for a bivariate model of height (${\mathrm{{Height}}}\sim F_{{\mathrm{ROH}}} + F_{{\mathrm{GRM}}}$). The reduction in height is more strongly associated with $F_{{\mathrm{ROH}}}$ than $F_{{\mathrm{GRM}}}$, as predicted if the causal variants are in weak LD with the common SNPs used to calculate $F_{{\mathrm{GRM}}}$ (Supplementary Note 5). The pattern is similar for other traits (Supplementary Fig. 15a, b; Supplementary Data 22). **c** *F*_ROH_ is a lower variance estimator of the inbreeding coefficient than *F*_GRM_. The ratio of $\beta _{F_{{\mathrm{GRM}}}}:\beta _{F_{{\mathrm{ROH}}}}$ is plotted against $\frac{{{\mathrm{{var}}}(F_{{\mathrm{ROH}}})}}{{{\mathrm{{var}}}(F_{{\mathrm{GRM}}})}}$ for all traits in all cohorts. When the variation of $F_{{\mathrm{GRM}}}$ which is independent of $F_{{\mathrm{ROH}}}$ has no effect on traits, $\hat \beta _{F_{{\mathrm{GRM}}}}$ is downwardly biased by a factor of $\frac{{{\mathrm{{var}}}(F_{{\mathrm{ROH}}})}}{{{\mathrm{{var}}}(F_{{\mathrm{GRM}}})}}$ (Supplementary Note 4). A linear maximum likelihood fit, shown in red, has a gradient consistent with unity [1.01; 95% CI 0.84–1.18], as expected when the difference between $F_{{\mathrm{GRM}}}$ and $F_{{\mathrm{ROH}}}$ is not informative about the excess homozygosity at causal variants (Supplementary Note 5). **d** *F*_ROH_ is a better predictor of rare variant homozygosity than *F*_GRM_. The excess homozygosities of SNPs, extracted from UK Biobank imputed genotypes, were calculated at seven discrete minor allele frequencies ($F_{{\mathrm{MAF}}}$), and regressed on two estimators of inbreeding in a bivariate statistical model (see Supplementary Note 5). The homozygosity of common SNPs is better predicted by $F_{{\mathrm{GRM}}}$, but rare variant homozygosity is better predicted by $F_{{\mathrm{ROH}}}$. The results from real data (Fig. 4b, Supplementary Figs 15a, b and Supplementary Data 22) are consistent with those simulated here, if the causal variants are predominantly rare. All errors bars represent 95% confidence intervals

The use of ROH to estimate inbreeding coefficients is relatively new in inbreeding research^11,16–19^. Earlier frequency-based estimators such as *F*_SNP_ and *F*_GRM_^20^, made use of excess marker homozygosity^21–23^ and did not require physical maps. We performed both univariate and multivariate regressions to evaluate the effectiveness of *F*_ROH_ against these measures. The correlations between them range from 0.13 to 0.99 and are strongest in cohorts with high average inbreeding (Supplementary Data 6, Supplementary Fig. 6). Significantly, univariate regressions of traits on both *F*_SNP_ and *F*_GRM_ show attenuated effect estimates relative to *F*_ROH_ (Supplementary Data 13). This attenuation is greatest in low autozygosity cohorts, suggesting that *F*_ROH_ is a better estimator of excess homozygosity at the causal loci (Fig. 4c).

To explore this further, we fit bivariate models with *F*_ROH_ and *F*_GRM_ as explanatory variables. For all 32 traits that were significant in the univariate analysis, we find that $\widehat \beta _{F_{\mathrm{ROH}}|F_{\mathrm{GRM}}}$ is of greater magnitude than $\widehat \beta _{F{\mathrm{GRM}}|F{\mathrm{ROH}}}$in the conditional analysis (Fig. 4b, Supplementary Data 22). This suggests that inbreeding depression is predominantly caused by rare, recessive variants made homozygous in ROH, and not by the chance homozygosity of variants in strong LD with common SNPs (Fig. 4d, Supplementary Note 5). We also find that ROH of different lengths have similar effects per unit length (Fig. 4a, Supplementary Fig. 11a), consistent with their having a causal effect on traits and not with confounding by socioeconomic or other factors, as shorter ROH arise from deep in the pedigree are thus less correlated with recent consanguinity.

### Quantifying the scope of social confounding

Previous studies have highlighted the potential for *F*_ROH_ to be confounded by non-genetic factors^6,24^. We therefore estimated the effect of *F*_ROH_ within various groups, between which confounding might be expected either to differ, or not be present at all.

**Fig. 5:**
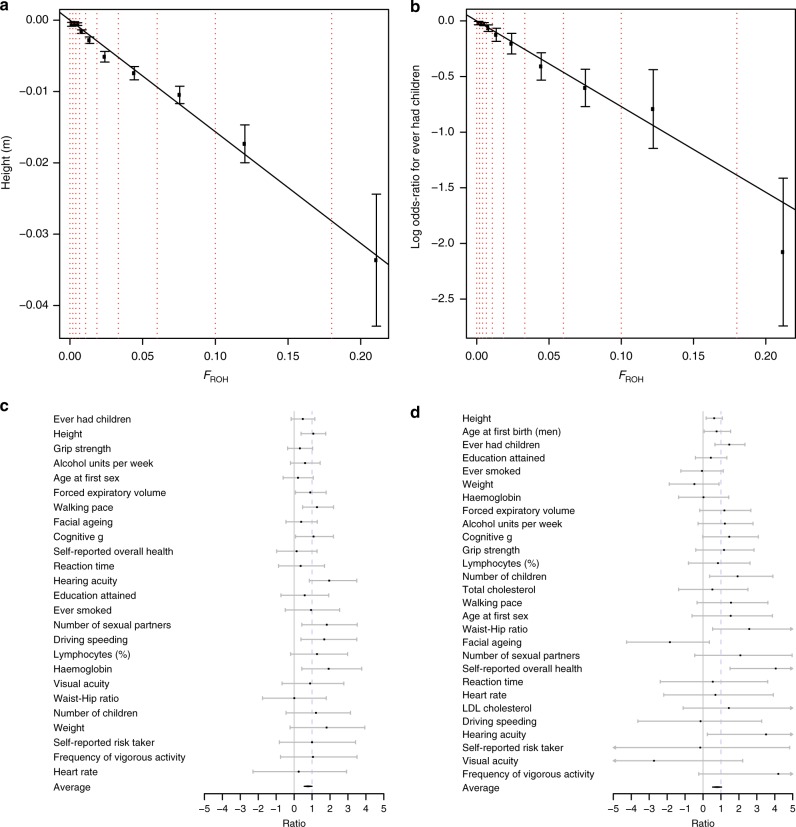
Evidence ROH effects are un-confounded. **a** Linear decrease in height with increasing *F*_ROH_. Average heights (in metres) is plotted in bins of increasing *F*_ROH_. The limits of each bin are shown by red dotted lines, and correspond to the offspring of increasing degree unions left-to-right. The overall estimate of $\beta _{F_{{\mathrm{ROH}}}}$ is shown as a solid black line. Subjects with kinship equal to offspring of full-sibling or parent–child unions are significantly shorter than those of avuncular or half-sibling unions who in turn are significantly shorter than those of first-cousin unions. **b** Linear decrease in odds of ever having children with increasing *F*_ROH_. Linear model approximations of ln(Odds-Ratio) for ever having children (1 = parous, 0 = childless) are plotted in bins of increasing *F*_ROH_. A strong relationship is evident, extending beyond the offspring of first cousins. **c** ROH effects are consistent in adoptees. The ratios of effect estimates, $\beta _{F_{{\mathrm{ROH}}}}$, between adoptees and all individuals are presented by trait. All traits are directionally consistent and overall show a strongly significant difference from zero (average = 0.78, 95% CI 0.56–1.00, *p* = 2 × 10^−12^). FEV1 forced expiratory volume in one second. **d** ROH effects are consistent in full siblings. The ratios of effect estimates within full siblings to effects in all individuals ($\beta _{F_{{\mathrm{ROH}}\_{\mathrm{wSibs}}}}:\beta _{F_{{\mathrm{ROH}}}}$) are presented by trait. Twenty-three of 29 estimates are directionally consistent and overall show a significant difference from zero (average = 0.78, 95% CI 0.53–1.04, *p* *=* 7 × 10^−10^). BMI body mass index. All errors bars represent 95% confidence intervals

For example, the effect of *F*_ROH_ on height is consistent across seven major continental ancestry groups (Supplementary Fig. 1, Supplementary Data 18), despite differing attitudes towards consanguinity, and consequently different burdens and origins of ROH. Similarly, grouping cohorts into consanguineous, more cosmopolitan, admixed and those with homozygosity due to ancient founder effects also shows consistent effects (Supplementary Fig. 2, Supplementary Data 19). Equally, categorising samples into bins of increasing *F*_ROH_ shows a dose-dependent response of the study traits with increased *F*_ROH_ (Supplementary Data 17 and Fig. 5a, b show the response for height and ever having children; Supplementary Figs 9a–f for all significant traits). The proportionality of these effects is consistent with a genetic cause, while it is difficult to envisage a confounder proportionally associated across the *entire* range of observed *F*_ROH_. In particular, the highest *F*_ROH_ group (*F*_ROH_ > 0.18), equivalent to the offspring of first-degree relatives, are found to be, on average, 3.4 [95% CI 2.5–4.3] cm shorter and 3.1 [95% CI 2.5–3.7] times more likely to be childless than an *F*_ROH_ = 0 individual.

Next, we estimated $\beta_{{F}_{\rm{ROH}}}$ for 7153 self-declared adopted individuals in UK Biobank, whose genotype is less likely to be confounded by cultural factors associated with the relatedness of their biological parents. For all 26 significant traits measured in this cohort, effect estimates are directionally consistent with the meta-analysis and 3 (height, walking pace and hearing acuity) reach replication significance (*p* < 0.004). In addition, a meta-analysis of the ratio $\widehat \beta _{F_{{\mathrm{ROH}}\_{\mathrm{ADOPTEE}}}}:\widehat \beta _{F_{\mathrm{ROH}}}$ across all traits differs significantly from zero (Fig. 5c; average = 0.78, 95% CI 0.56–1.00, *p* = 2 × 10^−12^).

Finally, the effect of *F*_ROH_ was estimated in up to 118,773 individuals in sibships (full-sibling pairs, trios, etc.: $\widehat \beta _{F_{\mathrm{ROH}\_{\mathrm{w{Sibs}}}}}$). *F*_ROH_ differences between siblings are caused entirely by Mendelian segregation, and are thus independent of any reasonable model of confounding. The variation of *F*_ROH_ among siblings is a small fraction of the population-wide variation^11^ (Supplementary Data 5); nevertheless, 23 out of 29 estimates of $\widehat \beta _{F_{{\mathrm{ROH}}\_{\mathrm{wSibs}}}}$ are directionally consistent with $\widehat \beta _{F_{\mathrm{ROH}}}$, and two (self-reported overall health and ever having children) reach replication significance. A meta-analysis of the ratio $\widehat \beta _{F_{{\mathrm{ROH}}\_{\mathrm{wSibs}}}}:\widehat \beta _{F_{\mathrm{ROH}}}$ for all traits is significantly greater than zero (Fig. 5d; average = 0.78, 95% CI 0.53–1.04, *p* *=* 7 × 10^−10^), indicating a substantial fraction of these effects is genetic in origin. However, for both adoptees and siblings, the point estimates are less than one, suggesting that non-genetic factors probably contribute a small, but significant, fraction of the observed effects.

## Discussion

Our results reveal inbreeding depression to be broad in scope, influencing both complex traits related to evolutionary fitness and others where the pattern of selection is less clear. While studies of couples show optimal fertility for those with distant kinship^25,26^, fewer have examined reproductive success as a function of individual inbreeding. Those that did are orders of magnitude smaller in size than the present study, suffer the attendant drawbacks of pedigree analysis, and have found mixed results^27–29^. Our genomic approach also reveals that in addition to socio-demographic factors and individual choice, recessive genetic effects have a significant influence on whether individuals reproduce. The discordant effects on fertility and education demonstrate that this is not just a result of genetic correlations between the two domains^30^.

The effects we see on fertility might be partially mediated through a hitherto unknown effect of autozygosity on decreasing the prevalence of risk-taking behaviours. Significant effects of autozygosity are observed for self-reported risk taking, speeding on motorways, alcohol and smoking behaviour, age at first sexual intercourse and number of sexual partners. Independent evidence for a shared genetic architecture between risk-taking and fertility traits comes from analysis of genetic correlations using LD-score regression in UKB (Supplementary Table 1). The core fertility traits, ever had children and number of children, are strongly genetically correlated (*r*_G_ = 0.93; *p* *<* 10^−100^). Genetic correlations with ever-smoking and self-reported risk-taking are lower, but also significant: 0.23–0.27, *p* *<* 10^−10^. Age at first sex is strongly genetically correlated both with the fertility traits, (*r*_G_ = 0.53–0.57), and number of sexual partners, ever-smoking and risk-taking^30^ (*r*_G_ = 0.42–0.60).

Reproductive traits are understandable targets of natural selection, as might be walking speed, grip strength, overall health, and visual and auditory acuity. While we cannot completely exclude reverse causality, whereby a less risk-taking, more conservative, personality is associated with greater likelihood of consanguineous marriage, we note that the effects are consistent for ROH < 5 Mb, which are less confounded with mate choice, due to their more distant pedigree origins (Supplementary Fig. 11a). This group of traits also shows similar evidence for un-confounded effects in the analysis of adoptees and full siblings (Fig. 5c, d; Supplementary Data 16) and the signals remained after correcting for religious activity or education.

On the other hand, for some traits that we expected to be influenced by ROH, we observed no effect. For example, birth weight is considered a key component of evolutionary fitness in mammals, and is influenced by genomic homozygosity in deer^31^; however, no material effect is apparent here (Supplementary Data 10). Furthermore, in one case, ROH appear to provide a beneficial effect: increasing *F*_ROH_ significantly decreases total and LDL-cholesterol in men, and may thus be cardio-protective in this regard.

Our multivariate models show that homozygosity at common SNPs outside of ROH has little influence on traits, and that the effect rather comes from ROH over 1.5 Mb in length. This suggests that genetic variants causing inbreeding depression are almost entirely rare, consistent with the dominance hypothesis^1^. The alternative hypothesis of overdominance, whereby positive selection on heterozygotes has brought alleles to intermediate frequencies, would predict that more common homozygous SNPs outside long ROH would also confer an effect. The differential provides evidence in humans that rare recessive mutations underlie the quantitative effects of inbreeding depression.

Previous studies have shown that associations observed between *F*_ROH_ and traits do not prove a causal relationship^14,24^. Traditional Genome-wide Association Studies (GWAS) can infer causality because, in the absence of population structure, genetic variants (SNPs) are randomly distributed between, and within, different social groups. However, this assumption does not hold in studies of inbreeding depression, where, even within a genetically homogeneous population, social groups may have differing attitudes towards consanguinity, and therefore different average *F*_ROH_ and, potentially, different average trait values. We therefore present a number of analyses that discount social confounding as a major factor in our results. Firstly, we show that the effects are consistent across diverse populations, including those where ROH burden is driven by founder effects rather than cultural practices regarding marriage. Effects are also consistent across a 20-fold range of *F*_ROH_: from low levels, likely unknown to the subject, to extremely high levels only seen in the offspring of first-degree relatives. Secondly, we show that the effects of ROH are consistent in direction and magnitude among adopted individuals, and also for short ROH which are not informative about parental relatedness. Finally, we introduce a within-siblings method, independent of all confounders, that confirms a genetic explanation for most of the observed effects. Variation in *F*_ROH_ between siblings is caused entirely by random Mendelian segregation; we show that higher *F*_ROH_ siblings experience poorer overall health and lower reproductive success, as well as other changes consistent with population-wide estimates. Nevertheless, average effect sizes from both adoptees and siblings are 20% smaller than population-wide estimates, confirming the importance of accounting for social confounding in future studies of human inbreeding depression.

Our results reveal five large groups of phenotypes sensitive to inbreeding depression, including some known to be closely linked to evolutionary fitness, but also others where the connection is, with current knowledge, more surprising. The effects are mediated by ROH rather than homozygosity of common SNPs, causally implicating rare recessive variants rather than overdominance as the most important underlying mechanism. Identification of these recessive variants will be challenging, but analysis of regional ROH and in particular using whole-genome sequences in large cohorts with sufficient variance in autozygosity will be the first step. Founder populations or those which prefer consanguineous marriage will provide the most power to understand this fundamental phenomenon.

see Supplementary Data.

## Methods

### Overview

Our initial aim was to estimate the effect of *F*_ROH_ on 45 quantitative traits and to assess whether any of these effects differed significantly from zero. Previous work^7,11^ has shown that inbreeding coefficients are low in most human populations, and that very large samples are required to reliably estimate the genetic effects of inbreeding^13^. To maximise sample size, a collaborative consortium (ROHgen^6^) was established, and research groups administering cohorts with SNP chip genotyping were invited to participate. To ensure that all participants performed uniform and repeatable analyses, a semi-automated software pipeline was developed and executed locally by each research group. This software pipeline required cohorts to provide only quality-controlled genotypes (in plink binary format) and standardised phenotypes (in plain-text) and used standard software (R, PLINK^12,32^, KING^33^) to perform the analyses described below. Results from each cohort were returned to the central ROHgen analysts for meta-analysis.

During the initial meta-analysis, genotypes were released for >500,000 samples from the richly phenotyped UK Biobank (UKB)^10^. It was therefore decided to add a further 34 quantitative phenotypes and 21 binary traits to the ROHgen analysis. Many of these additional traits were unique to UKB, although 7 were also available in a subset of ROHgen cohorts willing to run additional analyses. In total, the effect of *F*_ROH_ was tested on 100 traits and therefore experiment-wise significance was defined as 5 × 10^−4^ (=0.05/100).

### Cohort recruitment

In total, 119 independent, genetic epidemiological study cohorts were contributed to ROHgen. Of these, 118 were studies of adults and contributed multiple phenotypes, while 1 was a study of children and contributed only birth weight. To minimise any potential confounding or bias caused by within-study heterogeneity, studies were split into single-ethnicity sub-cohorts wherever applicable. Each sub-cohort was required to use only one genotyping array and be of uniform ancestry and case-status. For example, if a study contained multiple distinct ethnicities, sub-cohorts of each ancestry were created and analysed separately. At minimum, ancestry was defined on a sub-continental scale (i.e. European, African, East Asian, South Asian, West Asian, Japanese, and Hispanic were always analysed separately) but more precise separation was used when deemed necessary, for example, in cohorts with large representation of Ashkenazi Jews. In case-control studies of disease, separate sub-cohorts were created for cases and controls and phenotypes associated with disease status were not analysed in the case cohort: for example, fasting plasma glucose was not analysed in Type 2 diabetes case cohorts. Occasionally, cohorts had been genotyped on different SNP genotyping microarrays and these were also separated into sub-cohorts. There was one exception (deCODE) to the single microarray rule, where the intersection between all arrays used exceeded 150,000 SNPs. In this cohort the genotype data from all arrays was merged since the correspondence between *F*_ROH_ for the individual arrays and *F*_ROH_ the intersection dataset was found to be very strong ($\beta _{{\mathrm{merged}},{\mathrm{hap}}}$ = 0.98, *r*^2^ = 0.98; $\beta _{{\mathrm{merged}},{\mathrm{omni}}}$ = 0.97, *r*^2^=0.97). Dividing studies using these criteria yielded 234 sub-cohorts. Details of phenotypes contributed by each cohort are available in Supplementary Data 4.

### Ethical approval

Data from 119 independent genetic epidemiology studies were included. All subjects gave written informed consent for broad-ranging health and genetic research and all studies were approved by the relevant research ethics committees or boards. PubMed references are given for each study in Supplementary Data 2.

### Genotyping

All samples were genotyped on high-density (minimum 250,000 markers), genome-wide SNP microarrays supplied by Illumina or Affymetrix. Genotyping arrays with highly variable genomic coverage (such as Exome chip, Metabochip, or Immunochip) were judged unsuitable for the ROH calling algorithm and were not permitted. Imputed genotypes were also not permitted; only called genotypes in PLINK binary format were accepted. Each study applied their own GWAS quality controls before additional checks were made in the common analysis pipeline: SNPs with >3% missingness or MAF <5% were removed, as were individuals with >3% missing data. Only autosomal genotypes were used for the analyses reported here. Additional, cohort-specific, genotyping information is available in Supplementary Data 2.

### Phenotyping

In total, results are reported for 79 quantitative traits and 21 binary traits. These traits were chosen to represent different domains of health and reproductive success, with consideration given to presumed data availability. Many of these traits have been the subject of existing genome-wide association meta-analyses (GWAMA), and phenotype modelling, such as inclusion of relevant covariates, was copied from the relevant consortia (GIANT for anthropometry, EGG for birth weight, ICBP for blood pressures, MAGIC for glycaemic traits, CHARGE-Cognitive, -Inflammation & -Haemostasis working groups for cognitive function, CRP, fibrinogen, CHARGE-CKDgen for eGFR, CHARGE-ReproGen for ages at menarche and menopause, Blood Cell & HaemGen for haematology, GUGC for urate, RRgen, PRIMA, QRS & QT-IGC for electrocardiography, GLGC for classical lipids, CREAM for spherical equivalent refraction, Spirometa for lung function traits, and SSGAC for educational attainment and number of children ever born). Further information about individual phenotype modelling is available in Supplementary Note 1 and Supplementary Data 8.

### ROH calling

Runs of homozygosity (ROH) of >1.5 Mb in length were identified using published methods^6,11^. In summary, SNPs with minor allele frequencies below 5% were removed, before continuous ROH SNPs were identified using PLINK with the following parameters: homozyg-window-snp 50; homozyg-snp 50; homozyg-kb 1500; homozyg-gap 1000; homozyg-density 50; homozyg-window-missing 5; homozyg-window-het 1. No linkage disequilibrium pruning was performed. These parameters have been previously shown to call ROH that correspond to autozygous segments in which all SNPs (including those not present on the chip) are homozygous-by-descent, not chance arrangements of independent homozygous SNPs, and inbreeding coefficient estimates calculated by this method (*F*_ROH_) correlate well with pedigree-based estimates (*F*_PED_)^11^. Moreover, they have also been shown to be robust to array choice^6^.

### Calculating estimators of *F*

For each sample, two estimates of the inbreeding coefficient (*F*) were calculated, *F*_ROH_ and *F*_SNP_. We also calculated three additional measures of homozygosity: *F*_ROH<5Mb_, *F*_ROH>5Mb_ and *F*_SNP_outsideROH_.

*F*_ROH_ is the fraction of each genome in ROH >1.5 Mb. For example, in a sample for which PLINK had identified *n* ROH of length *l*_*i*_ (in Mb), *i* ϵ {1..*n*}, then *F*_ROH_ was then calculated as

1 $$\begin{array}{*{20}{c}} {F_{{\mathrm{ROH}}} = \frac{{\mathop {\sum }\nolimits_{i = 1}^n l_i}}{{3Gb}}} \end{array},$$

where *F*_ROH<5Mb_ and *F*_ROH>5Mb_ are the genomic fractions in ROH of length >5 Mb, and in ROH of length <5 Mb (but >1.5 Mb), respectively, and the length of the autosomal genome is estimated at 3 gigabases (Gb). It follows from this definition that

2 $$\begin{array}{*{20}{c}} {F_{{\mathrm{ROH}}} = F_{{\mathrm{ROH}} > 5{\mathrm{Mb}}} + F_{{\mathrm{ROH}} < 5{\mathrm{Mb}}}} \end{array}.$$

Single-point inbreeding coefficients can also be estimated from individual SNP homozygosity without any reference to a genetic map. For comparison with *F*_ROH_, a method of moments estimate of inbreeding coefficient was calculated^34^, referred to here as *F*_SNP_, and implemented in PLINK by the command–het.

3 $$\begin{array}{*{20}{c}} {F_{{\mathrm{SNP}}} = \frac{{O\left( {{\mathrm{{HOM}}}} \right) -E\left( {{\mathrm{{HOM}}}} \right)}}{{N - E\left( {{\mathrm{{HOM}}}} \right)}}} \end{array},$$

where *O*(HOM) is the observed number of homozygous SNPs, *E*(HOM) is the expected number of homozygous SNPs, i.e. $\mathop {\sum}\nolimits_{i = 1}^N {\left( {1 - 2p_iq_i} \right)}$, and *N* is the total number of non-missing genotyped SNPs.

*F*_ROH_ and *F*_SNP_ are strongly correlated, especially in cohorts with significant inbreeding, since both are estimates of *F*. To clarify the conditional effects of *F*_ROH_ and *F*_SNP_, an additional measure of homozygosity,*F*_SNPoutsideROH_, was calculated to describe the SNP homozygosity observed outside ROH.

4 $$\begin{array}{*{20}{c}} {F_{{\mathrm{SNP}}_{{\mathrm{outsideROH}}}} = \frac{{O\prime \left( {\mathrm{{HOM}}} \right) - E\prime \left( {\mathrm{{HOM}}} \right)}}{{N\prime - E\prime \left( {\mathrm{{HOM}}} \right)}}} \end{array},$$

where

5 $$\begin{array}{*{20}{c}} {O\prime \left( {{\mathrm{{HOM}}}} \right) = O\left( {{\mathrm{{HOM}}}} \right) - N_{{\mathrm{SNP}}\_{\mathrm{ROH}}}} \end{array},$$

6 $$\begin{array}{*{20}{c}} {E\prime \left( {\mathrm{{HOM}}} \right) = \left( {\frac{{N - N_{{\mathrm{ROH}}}}}{N}} \right) \ast E\left( {\mathrm{{HOM}}} \right)} \end{array},$$

7 $$N\prime = N - N_{{\mathrm{ROH}}}$$

And *N*_SNP_ROH_ is the number of homozygous SNPs found in ROH. Note that:

8 $${{F_{\mathrm{{SNP}}}}}_{{\mathrm{outsideROH}}} \approx F_{{\mathrm{SNP}}} - F_{{\mathrm{ROH}}}$$

A further single point estimator of the inbreeding coefficient, described by Yang et al.^20^ as $\widehat F^{{\mathrm{{III}}}}$, is implemented in PLINK by the parameter –ibc (Fhat3) and was also calculated for all samples.

9 $$F_{{\mathrm{GRM}}} = \widehat F^{\mathrm{{III}}} = \frac{1}{N}\mathop {\sum}\nolimits_{i = 1}^N {\frac{{\left( {x_i^2 - \left( {1 + 2p_i} \right)x_i + 2p_i^2} \right)}}{{2p_i\left( {1 - p_i} \right)}}},$$

where *N* is the number of SNPs, _*pi*_ is the reference allele frequency of the *i*th SNP in the sample population and *x*_*i*_ is the number of copies of the reference allele.

### Effect size estimates for quantitative traits

In each cohort of *n* samples, for each of the quantitative traits measured in that cohort, trait values were modelled by

10 $$\begin{array}{*{20}{c}} {y = \beta _{F_{{\mathrm{ROH}}}} \ast {\mathbf{F}}_{{\mathbf{ROH}}} + {\mathbf{{Xb}}} +{\boldsymbol{\varepsilon}}} \end{array},$$

where **y** is a vector (*n* × 1) of measured trait values, $\beta_{{F}_{\rm{ROH}}}$ is the unknown scalar effect of *F*_ROH_ on the trait, **F**_**ROH**_ is a known vector (*n* × 1) of individual *F*_ROH_, **b** is a vector (*m* × 1) of unknown fixed covariate effects (including a mean, *μ*), **X** in a known design matrix (*n* × *m*) for the fixed effects, and **ε** is an unknown vector (*n* × 1) of residuals.

The *m* fixed covariates included in each model were chosen with reference to the leading GWAMA consortium for that trait and are detailed in Supplementary Data 8. For all traits, these covariates included: age (and/or year of birth), sex, and at least the first 10 principal components of the genomic relatedness matrix (GRM). Where necessary, additional adjustments were made for study site, medications, and other relevant covariates (Supplementary Data 3).

For reasons of computational efficiency, it was decided to solve Eq. (10) in two steps. In the first step, the trait (**y**) was regressed on all fixed covariates to obtain the maximum likelihood solution of the model:

11 $$\begin{array}{*{20}{c}} {\mathbf{y}} = {\mathbf{{Xb}}} + \boldsymbol{\varepsilon}\prime \end{array}.$$

All subsequent analyses were performed using the vector of trait residuals **ε′**, which may be considered as the trait values corrected for all known covariates.

In cohorts with a high degree of relatedness, mixed-modelling was used to correct for family structure, although, because ROH are not narrow-sense heritable, this was considered less essential than in Genome-Wide Association Studies. Equation (11) becomes

12 $$\mathbf{y} = {\mathbf{{Xb}}} + {\mathbf{u}} + \boldsymbol{\varepsilon}\prime,$$

where **u** is an unknown vector (*n* × 1) of polygenic effects with multivariate normal distribution of mean 0 and covariance matrix *σ*_*g*_^2^**A**, where **A** is the genomic relationship matrix (GRM). In these related cohorts, a GRM was calculated using PLINK v1.9 and Grammar+ residuals of Eq. (12) were estimated using GenABEL^35^. These Grammar+ residuals (**ε′**) were used in subsequent analyses.

To estimate $\beta_{{F}_{\rm{ROH}}}$ for each trait, trait residuals were regressed on *F*_ROH_ to obtain the maximum likelihood (ML) solution of the model

13a $${\boldsymbol{\varepsilon }}\prime = \mu + \beta _{F_{\mathrm{ROH}}} \ast {\mathbf{F}}_{{\mathbf{ROH}}} + {\boldsymbol{\varepsilon}}.$$

The sex-specific estimates of $\beta_{{F}_{\rm{ROH}}}$ (Supplementary Data 12) were obtained from Eq. (13) applied to the relevant sex.

For all traits, a corresponding estimates of $\beta_{{F}_{\rm{SNP}}}$ and $\beta_{\rm{F}_{\rm{GRM}}}$ were obtained from the models

13b $${\boldsymbol{\varepsilon }}\prime = \mu + \beta _{F_{\mathrm{SNP}}} \ast {\mathbf{F}}_{{\mathbf{SNP}}} + {\boldsymbol{\varepsilon}},$$

14 $${\boldsymbol{\varepsilon }}\prime = \mu + \beta _{F_{\mathrm{GRM}}} \ast {\mathbf{F}}_{{\mathbf{GRM}}} + {\boldsymbol{\varepsilon}}$$

and the effects of different ROH lengths and of SNP homozygosity (Fig. 4b) were obtained from the model

15 $$\begin{array}{l}{\boldsymbol{\varepsilon }}\prime = \mu + \left( {\beta _1 \ast {\mathbf{F}}_{{\mathbf{SNP}}_{\mathrm{outsideROH}}}} \right) + \left( {\beta _2 \ast {\mathbf{F}}_{{\mathbf{ROH}} < 5{\mathbf{Mb}}}} \right)\\ {\hskip -62pt}+ \left( {\beta _3 \ast {\mathbf{F}}_{{\mathbf{ROH}} > 5{\mathbf{Mb}}}} \right) + {\boldsymbol{\varepsilon }} \end{array}.$$

### Effect size estimates for binary traits

Binary traits were analysed by two methods. The primary estimates of $\beta_{{F}_{\rm{ROH}}}$ (Fig. 3b and Supplementary Data 10) were obtained from full logistic models:

16 $$\begin{array}{*{20}{c}} {g\left( {E\left[ {\mathbf{y}} \right]} \right) ={\mathbf{{Xb}}}} \end{array},$$

where *g*() is the link function (logit), and where *F*_ROH_ and all applicable covariates (Supplementary Datas 3, 8) were fitted simultaneously. Mixed modelling for family structure was not attempted in the logistic models since an accepted method was not apparent.

For all subsequent results, **y** was scaled by $1/\sigma _y^2$ and analysed by linear models, as for quantitative traits, including mixed-modelling where appropriate for family studies. This method of estimating binary traits with simple linear models gives asymptotically unbiased estimates of $\beta_{{F}_{\rm{ROH}}}$ and se($\beta_{{F}_{\rm{ROH}}}$) on the ln(Odds-Ratio) scale^36^. For all significant binary traits, a comparison of $\widehat \beta _{F_{\mathrm{ROH}}}$ from the full model with $\widehat \beta _{F_{\mathrm{ROH}}}$ from the linear model approximation is presented in Supplementary Fig. 8.

To give $\widehat \beta _{F_{\mathrm{ROH}}}$ a more tangible interpretation, effect estimates are frequently quoted in the text as *β*_0.0625_, i.e. the estimated effect in the offspring of first cousins, where 6.25% of the genome is expected to be autozygous.

### Religiosity and educational attainment as additional covariates

To assess the importance of potential social confounders, proxy measures of socio-economic status and religiosity were separately included in Eq. (13) as additional covariates. The modified effect estimates ($\widehat {\beta \prime }_{F_{\mathrm{ROH}}}$) were tested for significance (Supplementary Data 20) and compared to the uncorrected estimates ($\beta_{{F}_{\rm{ROH}}}$) (Supplementary Fig. 10a, b).

Since Educational Attainment (EA) was measured in many cohorts, this was chosen as the most suitable proxy for socio-economic status. However, since *F*_ROH_ is known to affect EA directly^6^ any change in $\beta_{{F}_{\rm{ROH}}}$ when conditioning on EA cannot be assumed to be entirely due to environmental confounding.

The analysis of religiosity was only carried out in UKB, where a rough proxy was available. Although no direct questions about religious beliefs were included, participants were asked about their leisure activities. In response to the question *Which of the following do you attend once a week or more often? (You can select more than one)*, 15.6% of UKB participants selected *Religious Group* from one of the seven options offered. In the models described, religiosity was coded as 1 for those who selected *Religious Group* and 0 for those who did not. Although this is likely to be an imperfect measure of actual religious belief it is currently the best available in a large dataset.

### Assortative mating

Humans are known to mate assortatively for a number of traits including height and cognition^37^, and so we sought to investigate if this could influence our results, for example, by the trait extremes being more genetically similar and thus the offspring more homozygous. We see no evidence for an effect of assortative mating on autozygosity, however. Firstly, a polygenic risk score for height (see Supplementary Note 1), which explains 18.7% of the phenotypic variance in height, was not associated with *F*_ROH_ (*p* = 0.77; Supplementary Fig. 5). Secondly, linear relationships between traits and autozygosity extend out to very high *F*_ROH_ individuals (Supplementary Figs. 9a–f). Samples in the highest *F*_ROH_ group are offspring of genetically similar parents, very likely first or second degree relatives and, for example, the height of these samples is on average 3.4 cm [95% CI 2.5–4.3] shorter than the population mean. Assortative mating would suggest this height deficit has been inherited from genetically shorter parents, but this would require an implausibly strong relationship between short stature and a propensity to marry a very close relative. Thirdly, the sex-specific effects we observe could only be explained by assortative mating if the additive heritability of these traits also differed by gender.

### Average trait values in groups of similar *F*_ROH_

In each cohort individuals were allocated to one of ten groups of similar *F*_ROH_. The bounds of these groups were the same for all cohorts, specifically {0, 0.002, 0.0041, 0.0067, 0.0108, 0.0186, 0.0333, 0.06, 0.10, 0.18 and 1.0}. Within each group the mean trait residual (**ε′**) and mean *F*_ROH_ were calculated, along with their associated standard errors. Within each cohort the expectation of **ε′** is zero at the mean *F*_ROH_, however as mean *F*_ROH_ varies between cohorts (Fig. 2, Supplementary Data 5) it was necessary to express **ε′** relative to a common *F*_ROH_ before meta-analysis. Hence, for this analysis only, the trait residuals (**ε′**) were expressed relative to the *F*_ROH_ = 0 intercept, i.e. by subtracting *μ* from Eq. (13).

### Effect of *F*_ROH_ within adoptees

We compared $\beta_{{{F}_{\rm{ROH}\_{\rm{ADOPTEE}}}}}$ to cross-cohort $\beta_{{F}_{\rm{ROH}}}$, not that from UKB alone, as we consider the latter to be a noisy estimate of the former; estimates in UKB are consistent with those from meta-analysis.

### Effect of *F*_ROH_ within full-sibling families

In a subset of cohorts, with substantial numbers of related individuals, further analyses were performed to investigate the effect of *F*_ROH_ within full-sibling families. In each of these cohorts, all second-degree, or closer, relatives were identified using KING (parameters:–related–degree 2). Full-siblings were then selected as relative pairs with genomic kinship >0.175 and IBS0 >0.001. This definition includes monozygotic twins, who were intentionally considered as part of full-sibling families. Although monozygotic twins are expected to have identical *F*_ROH_, they may not have identical trait values, and including additional trait measurements decreases the sampling error of the within-family variance estimate, hence increasing statistical power. Dizygotic twins were also included.

For each individual (*j)* with identified siblings, the values of *F*_ROH_ and trait residual (**ε′**) were calculated relative to their family mean (and called *F*_*j*_^ROH_wSibs^ and *ε*_*j*_^wSibs^, respectively), i.e. for individual *j* with *n* full-siblings *S*_*k*_ where *k* ϵ {1..*n*}

17 $$\begin{array}{*{20}{c}} {F_j^{{\mathrm{ROHwSibs}}} = F_j^{{\mathrm{ROH}}} - \frac{1}{{\left( {n + 1} \right)}}\mathop {\sum}\nolimits_{i{\it{\epsilon }}\left\{ {j,S_k} \right\}} {F_i^{{\mathrm{ROH}}}} } \end{array},$$

18 $$\begin{array}{*{20}{c}} {\varepsilon _j^{{\mathrm{wSibs}}} = \varepsilon _j^\prime - \frac{1}{{\left( {n + 1} \right)}}\mathop {\sum}\nolimits_{i{\it{\epsilon }}\left\{ {j,S_k} \right\}} {\varepsilon \prime _i} } \end{array}.$$

The effect of *F*_ROH_ within-full-siblings ($\beta_{{F}_{\rm{ROH}}\_{\rm{wSibes}}}$) was estimated by linear regression of **ε**^**wSibs**^ on **F**^**ROH_wSibs**^.

Importantly, the variation of *F*_ROH_ within full-siblings is entirely caused by differences in Mendelian segregation, and is therefore completely independent of all possible confounders. Hence, the effect estimates obtained by this method are estimates of the genetic effects of *F*_ROH_, unbiased by any possible confounder. Since confounding by social factors is a major concern in this field, methods that can definitively exclude this possibility are of critical importance.

### Between-cohort meta-analysis

As is typical in genome-wide association meta-analyses (GWAMA), genetic effects were estimated within single-ethnicity sub-cohorts, and meta-analysis of the within-cohort effect sizes was used to combine results^38^. This established method eliminates any potential confounding caused by between-cohort associations between *F*_ROH_ and traits.

Each cohort returned estimates and standard errors of: $\beta_{{F}_{\rm{ROH}}}$, $\beta_{{F}_{\rm{SNP}}},\beta_{{F}_{\rm{ROH} > {\rm{Mb}}}},\beta_{{F}_{\rm{ROH} < {\rm{Mb}}}},\beta_{{F}\_{\rm{outsideROH}}}, \beta_{{F}_{\rm{ROH}\_{\rm{wSibs}}}}$, as well as trait means ($\overline {\varepsilon \prime }$) and standard errors within each of 10 *F*_ROH_ bins. The between-cohort mean of each of these 16 estimates was then determined by fixed-effect, inverse-variance meta-analysis using the R package metafor^39^. Results shown in Figs. 3–5 are meta-analysed averages of the within-cohort effects.

The meta-analysis was also run for various subsets of cohorts, stratified by ancestry as defined in Supplementary Data 18. Meta-analysis estimates from these groupings are shown in Supplementary Fig. 1.

### Median and 95% CI of a ratio

In the analyses of adoptees (Fig. 5c), siblings (Fig. 5d) and potential confounders (Supplementary Figs. 10a, b) we wished to compare the effect estimates ($\beta_{{F}_{\rm{ROH}}}$) from two different methods across a wide range of traits. The units of $\beta_{{F}_{\rm{ROH}}}$ differ by trait so, to allow comparison across all traits, the unitless ratio of effect size estimates was calculated (for example $\beta_{{F}_{\rm{ROH}\_{\rm{wSibs}}}}$: $\beta_{{F}_{\rm{ROH}}}$). Figure 5c, d and Supplementary Figs. 10a, b show the medians and 95% CI of these ratios. These were determined empirically by bootstrap since, although formulae exist for the mean and standard error of a ratio^40^, the assumption of normality is violated when $\beta_{{F}_{\rm{ROH}}}$/se($\beta_{{F}_{\rm{ROH}}}$) is not large.

### Genetic correlations in UK Biobank

Genetic correlations were calculated using LD-Score Regression^41^, implemented in LDSC v1.0.0 (https://github.com/bulik/ldsc). Summary statistics were parsed using default parameters in the LDSC ‘munge_sumstats.py’ script, extracting only variants present in the HapMap 3 reference panel.

### Accuracy of *F*_ROH_ measures of inbreeding effects

A recent paper suggested that ROH may overestimate inbreeding effects by as much as 162%^42^; however, this could only be the case if *F*_ROH_ underestimates excess homozygosity at the causal loci by at least 162%. We do not believe this to be the case since the maximum *F*_ROH_ measured in many cohorts is around 0.25 (the expectation in the offspring off first-degree relatives), and the effect size estimates from these samples are consistent with the overall estimates (Fig. 5c, d and Supplementary Fig. 9a–f). We note that Yengo et al. applied the ROH calling parameters used here to imputed data. These parameters have been validated for called genotype data^6^ but not, to our knowledge, for the higher SNP density and error rate of imputed data (see also Supplementary Note 4). The simple method for detecting ROH used here was well suited to our study, since it could be easily implemented on over one million samples, and most of the variation in *F*_ROH_ is caused by easily-identified long ROH.^43–45^

### Reporting summary

Further information on research design is available in the Nature Research Reporting Summary linked to this article.

## Supplementary information

Supplementary Information

Data 1

Data 2

Data 3

Data 4

Data 5

Data 6

Data 7

Data 8

Data 9

Data 10

Data 11

Data 12

Data 13

Data 14

Data 15

Data 16

Data 17

Data 18

Data 19

Data 20

Data 21

Data 22

Description of Additional Supplementary Files

Reporting Summary

Peer Review File

## Data Availability

The meta-analysed data which support these findings are available as Supplementary Data files. Cohort-level summary statistics underlying all figures and tables are available in a publicly accessible dataset (10.6084/m9.figshare.9731087). In the majority of cases we do not have consent to share individual-level data, although for UK Biobank this is available on request from https://www.ukbiobank.ac.uk/.
