# Supplementary material for: Associations of autozygosity with a broad range of human phenotypes
Source: Nat Commun. 2019 Oct 31;10:4957. doi: 10.1038/s41467-019-12283-6 (PMC6823371; doi:10.1038/s41467-019-12283-6)
Supplement: Supplementary file 1 — Supplementary Information [file 41467_2019_12283_MOESM1_ESM.pdf]

SUPPLEMENTARY FIGURES

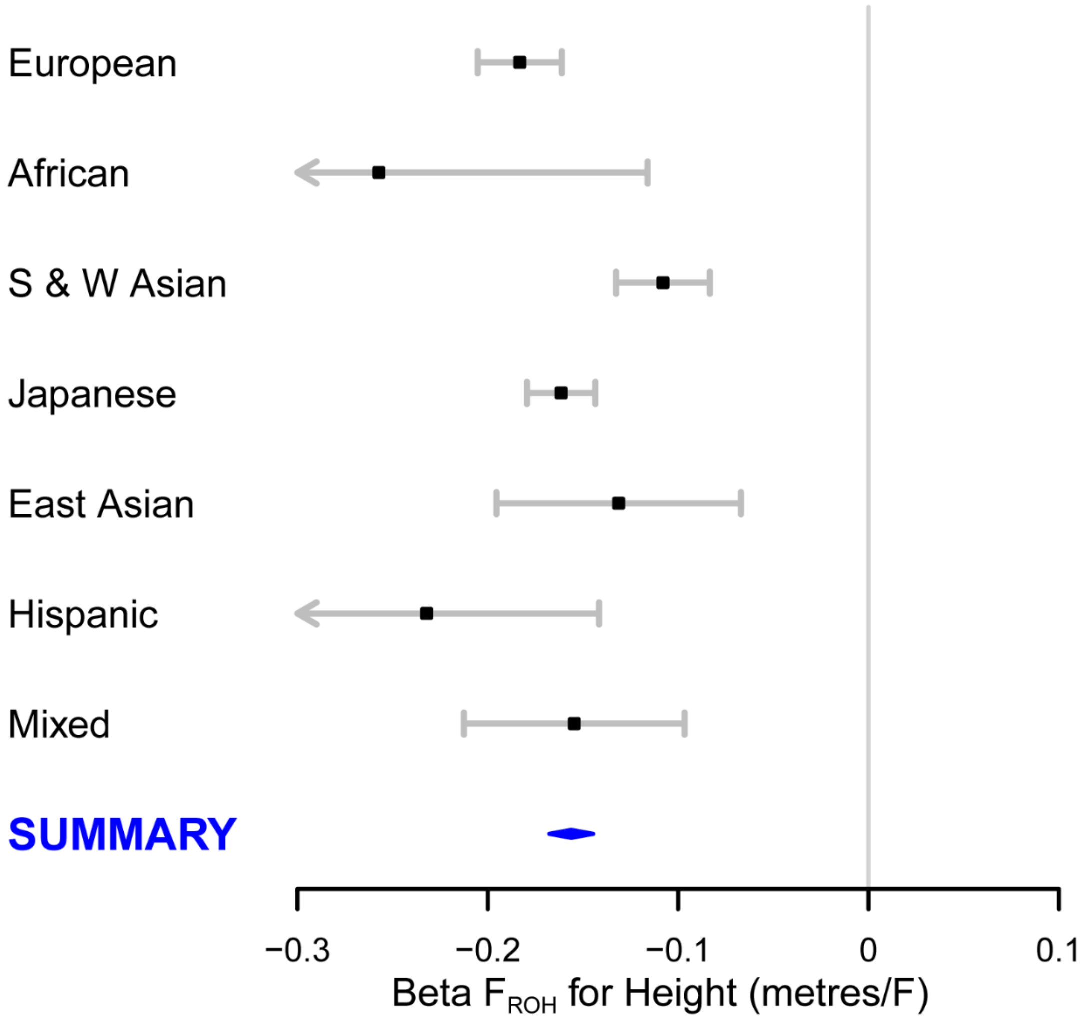

Supplementary Figure 1: Effect of F_{ROH} on height is robust to stratification by ancestral group. Cohorts were divided into eight broad ancestral groups (Supplementary Data Table 1) and meta-analysed separately. Although some heterogeneity is observed (heterogeneity *p*-value = 3 x 10^{-4}), $\beta_{F_{\mathrm{ROH}}}$ is directionally consistent and differs significantly from 0 in all ancestral groups. All errors bars represent 95% confidence intervals.

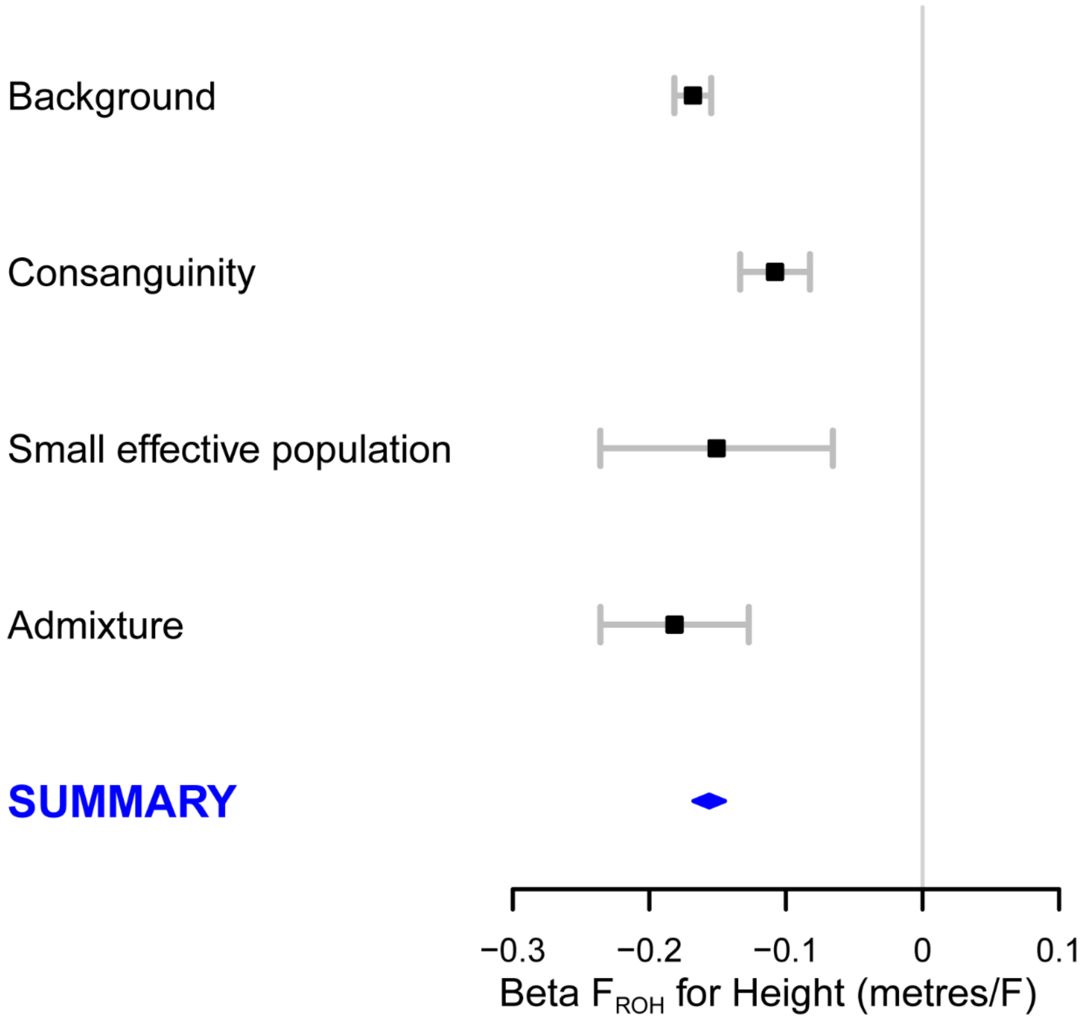

Supplementary Figure 2: Effect of F_{ROH} on height is robust to stratification by inferred demographic history. Cohorts were divided by inferred demographic history (Supplementary Fig. 4, Supplementary Data Table 1) and meta-analysed separately. A small amount of heterogeneity is observed (heterogeneity *p*-value = 0.008), but $\beta_{F_{\mathrm{ROH}}}$ is directionally consistent and differs significantly from 0 in all groups. In particular, in the small effective population size cohorts, where the variation of F_{ROH} is believed to be caused variations in cryptic relatedness between parents, $\beta_{F_{\mathrm{ROH}}}$ [-0.15, 95% CI -0.07 -0.23, *p*-value 3 x 10^{-4}] is consistent with the global estimate. All errors bars represent 95% confidence intervals.

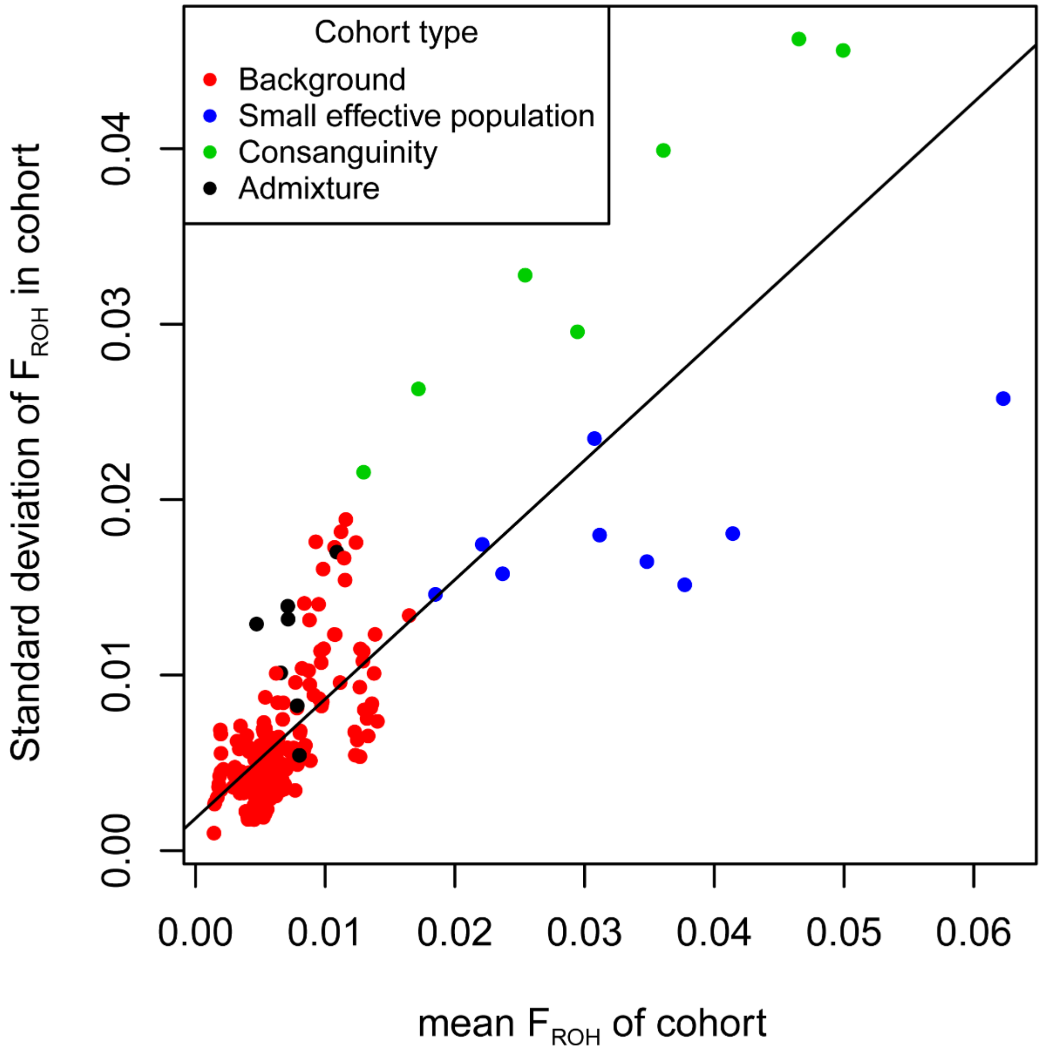

Supplementary Figure 3: A strong correlation (*r*=0.82, *p*-value = 9 x 10^{-103}) is observed between $\sigma_{F_{\mathrm{ROH}}}$ and mean F_{ROH}. The standard deviation of F_{ROH} ($\sigma_{F_{\mathrm{ROH}}}$) is plotted against mean F_{ROH} for all cohorts. In regressions on F_{ROH} the statistical power is approximately proportional to $\sigma^2_{F_{\mathrm{ROH}}}$ and cohorts with high mean F_{ROH} generally provide greater per-sample statistical power. Also, for a given mean F_{ROH}, cohorts where ROH are primarily attributable to consanguinity rather than small effective population size provide greater statistical power.

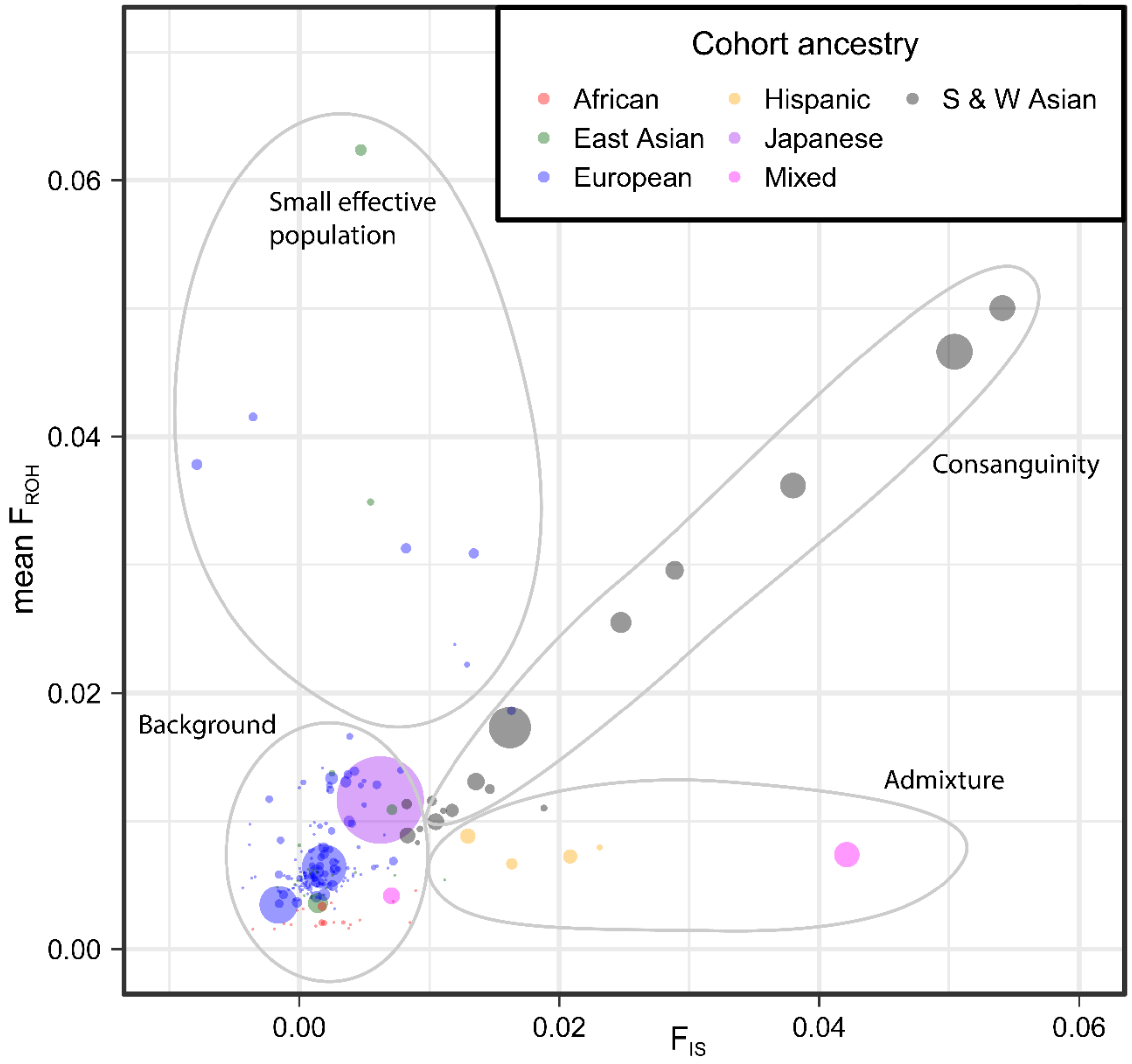

Supplementary Figure 4: Assignment of cohorts to one of four inferred demographic histories. Fig. 2 is replicated (see also Fig. 2 legend) and used to empirically assign cohorts to one of four inferred demographic histories. In cohorts where $F_{IS} > 0.1$, but the Cartesian distance to the 1:1 line was < 0.005, consanguinity was inferred to be the main origin of ROH. Cohorts which had not been defined as consanguineous but had mean $F_{ROH} > 0.02$ were consider to have a small effective population. Cohorts with $F_{IS} > 0.1$, but not consanguineous nor small effective population, were defined as admixed and the remaining cohorts were described as *background*.

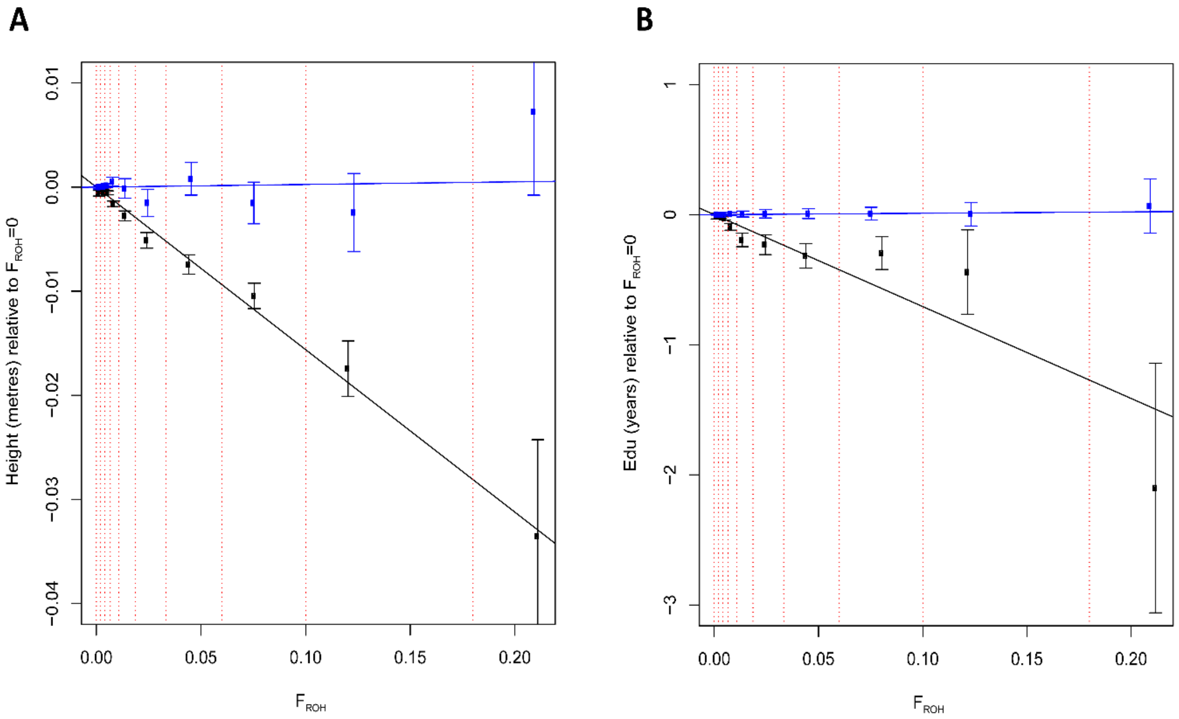

Supplementary Figure 5: Effect of assortative mating on height and educational attainment (a): Linear decrease in height with increasing F_{ROH} but no decrease in a polygenic score for height. In black, average height (in metres) is plotted in bins of increasing F_{ROH}. In blue, averages of a polygenic risk score for height are plotted in the same bins. Increased F_{ROH} is not associated with decreased polygenic score for height, providing evidence against a hypothesis of assortative mating generating the relationship with height. **(a): Decrease in education attained (EA) with increasing F_{ROH} but no decrease in a polygenic score for EA.** In black, average EA (in years) is plotted in bins of increasing F_{ROH}. In blue, average polygenic risk score for EA are plotted in the same bins. Increased F_{ROH} is not associated with decreased polygenic score for EA, providing evidence against a hypothesis of assortative mating generating the relationship with EA. All errors bars represent 95% confidence intervals.

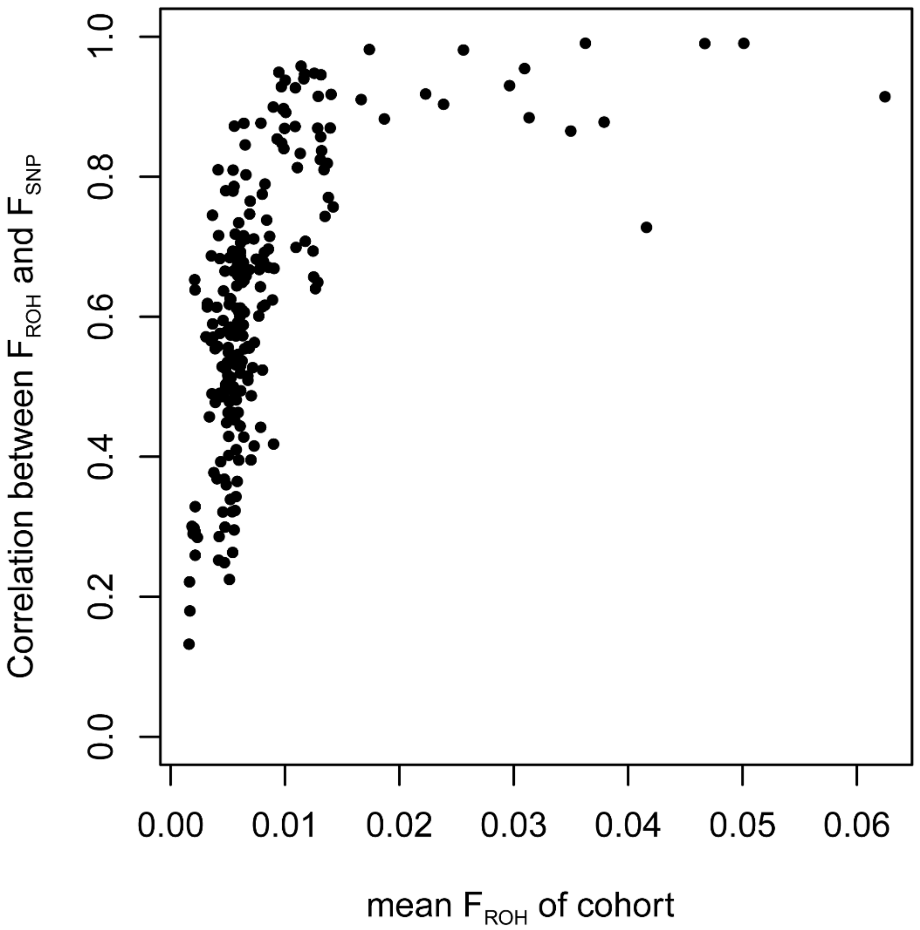

Supplementary Figure 6: Strong correlations between F_{ROH} and F_{SNP} are observed in cohorts with high average F_{ROH}. The correlation between F_{ROH} and F_{SNP} is plotted against mean F_{ROH} for all cohorts. In low autozygosity cohorts the correlation between F_{ROH} and F_{SNP} is weak to moderate, as only a small fraction of homozygous SNPs is found in ROH. In contrast, in higher autozygosity cohorts, ROH represent a larger fraction of homozygous SNPs and the correlation between F_{ROH} and F_{SNP} is stronger.

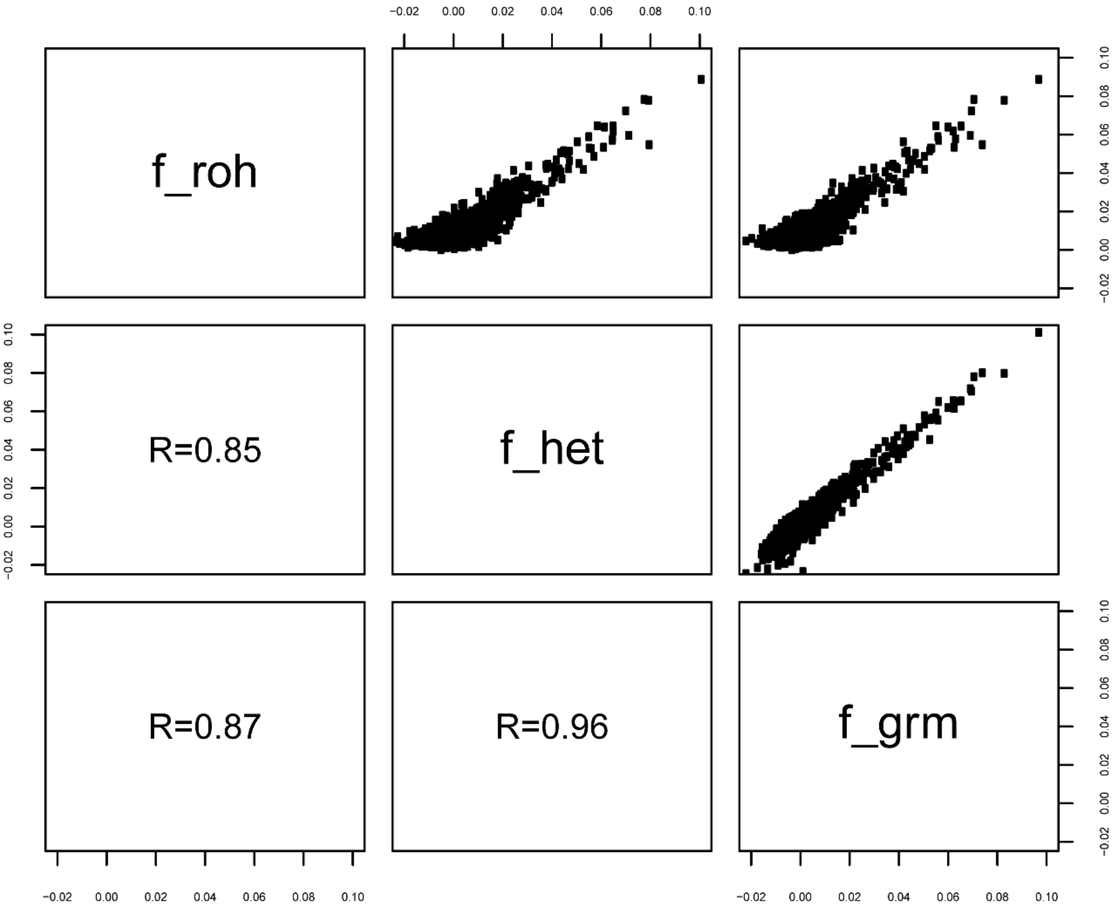

Supplementary Figure 7: Scatter plots of F_{ROH} plotted against F_{SNP} and F_{GRM} in a single cohort (VIKING). Scatter plots of three estimates of inbreeding coefficient (F_{ROH}, F_{SNP} and F_{GRM}) are shown in the upper right panels. The correlation between these estimates is shown in the lower left panels.

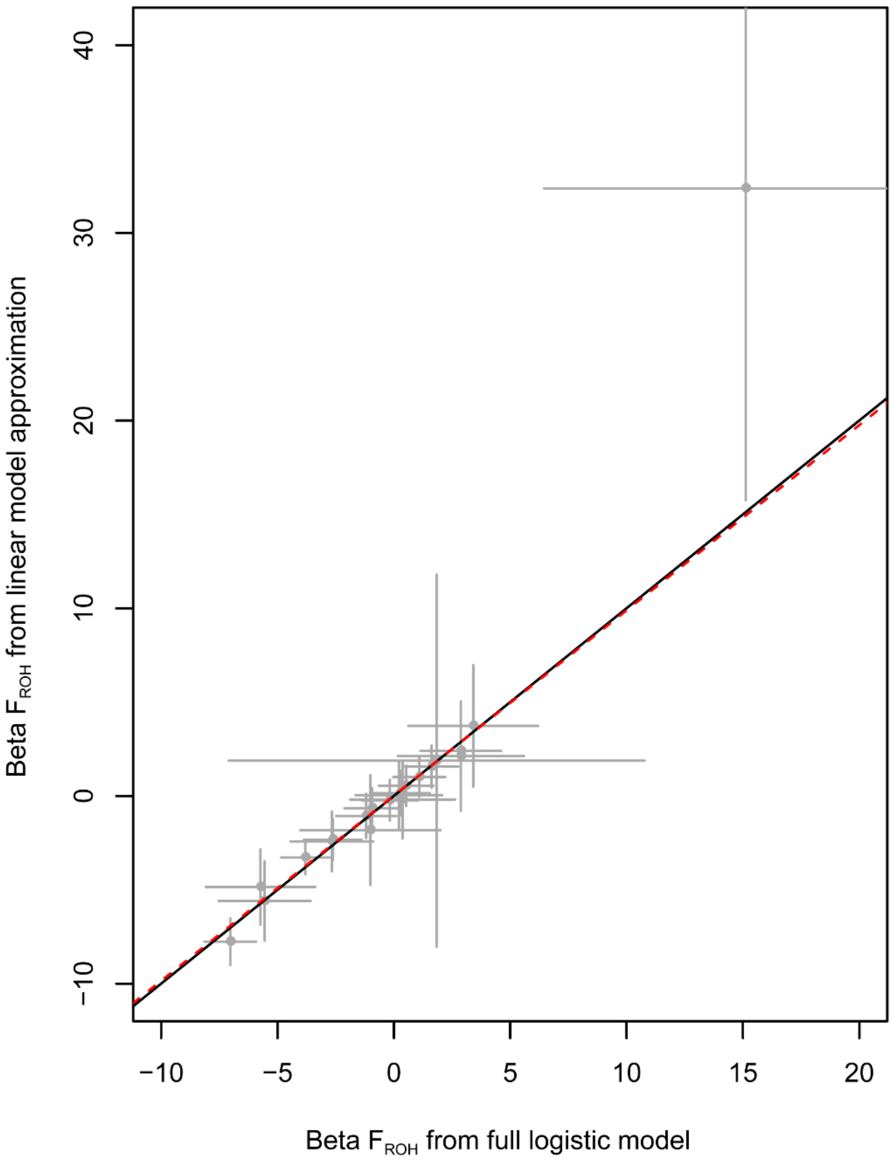

Supplementary Figure 8: A linear model approximation of the full logistic model gives relatively unbiased estimates of $\beta_{F_{\mathrm{ROH}}}$. For all 22 binary traits analysed, estimates obtained from a two-step linear model approximation are plotted against estimates obtained from the full logistic model (See Methods). Estimates of $\beta_{F_{\mathrm{ROH}}}$ are shown in grey. The 1:1 unity line is shown in red, and a linear least-squares fit is shown in black. The gradient of the best fit line (1.02 95% CI 0.99-1.02) does not differ significantly from the unbiased expectation of 1 (*p*-value 0.87). For all but one trait, the linear model approximation is consistent with the full logistic model estimate of $\beta_{F_{\mathrm{ROH}}}$. Self-declared infertility has the most extreme case:control ratio (632:472544) of any of the binary traits analysed and for this trait only the linear model significantly overestimates $\beta_{F_{\mathrm{ROH}}}$. The linear model estimates are therefore marked with an asterisk where they appear in Supplementary Data Tables 12-14, 16-21. All errors bars represent 95% confidence intervals.

9a

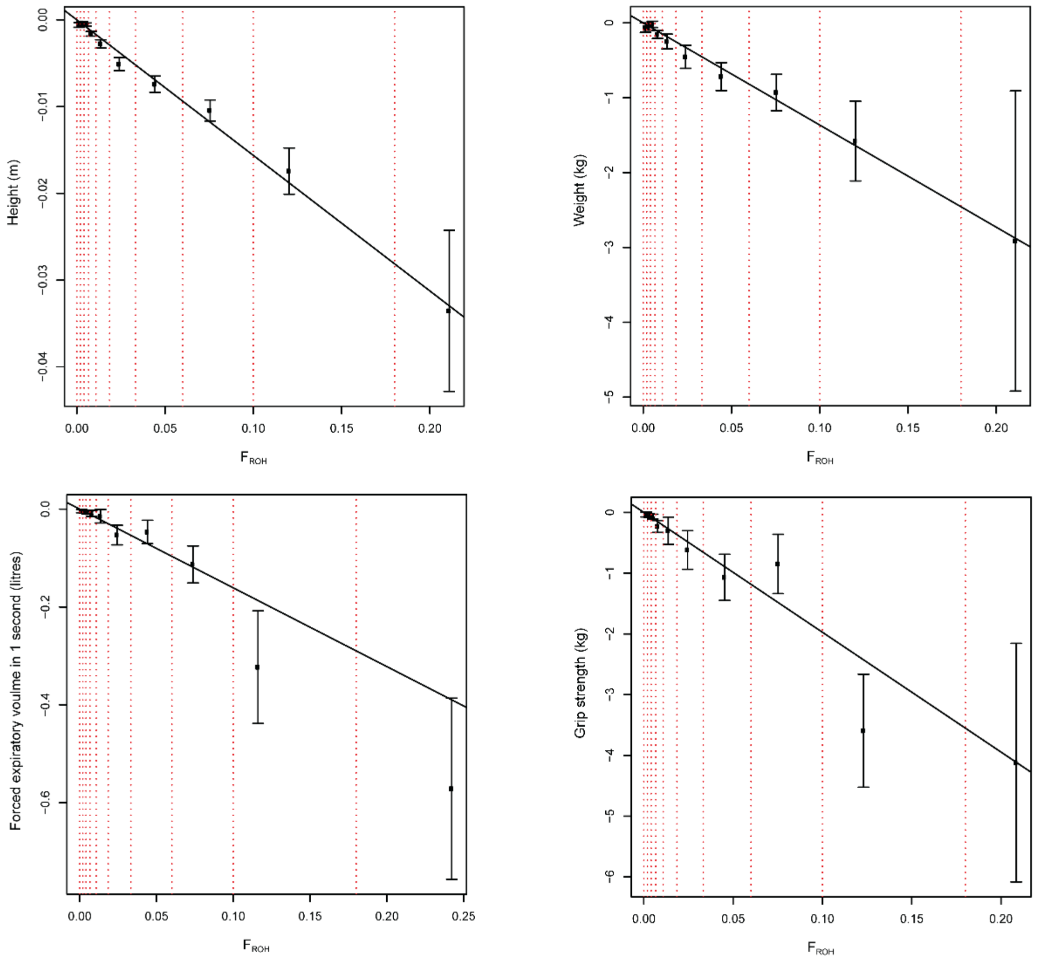
Height (m)
0.00
−0.01
−0.02
−0.03
−0.04
0.00 0.05 0.10 0.15 0.20
F ROH
Weight (kg)
0
−1
−2
−3
−4
−5
0.00 0.05 0.10 0.15 0.20
F ROH
Forced expiratory voulme in 1 second (litres)
0.0
−0.2
−0.4
−0.6
0.00 0.05 0.10 0.15 0.20 0.25
F ROH
Grip strength (kg)
0
−1
−2
−3
−4
−5
−6
0.00 0.05 0.10 0.15 0.20
F ROH

9b

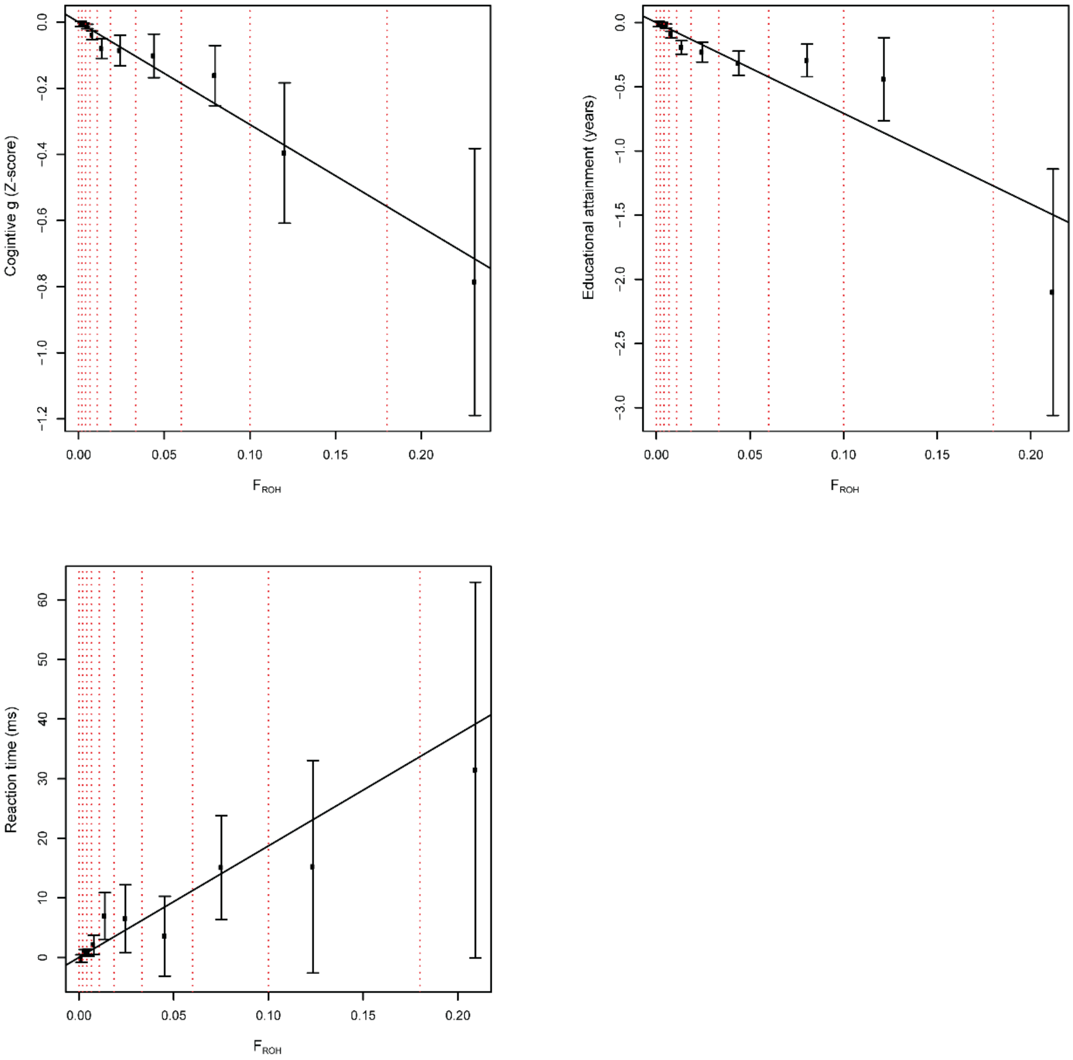
Cogintive g (Z-score)
0.0
−0.2
−0.4
−0.6
−0.8
−1.0
−1.2
0.00
0.05
0.10
0.15
0.20
F ROH
Educational attainment (years)
0.0
−0.5
−1.0
−1.5
−2.0
−2.5
−3.0
0.00
0.05
0.10
0.15
0.20
F ROH
Reaction time (ms)
60
50
40
30
20
10
0
0.00
0.05
0.10
0.15
0.20
F ROH

9c

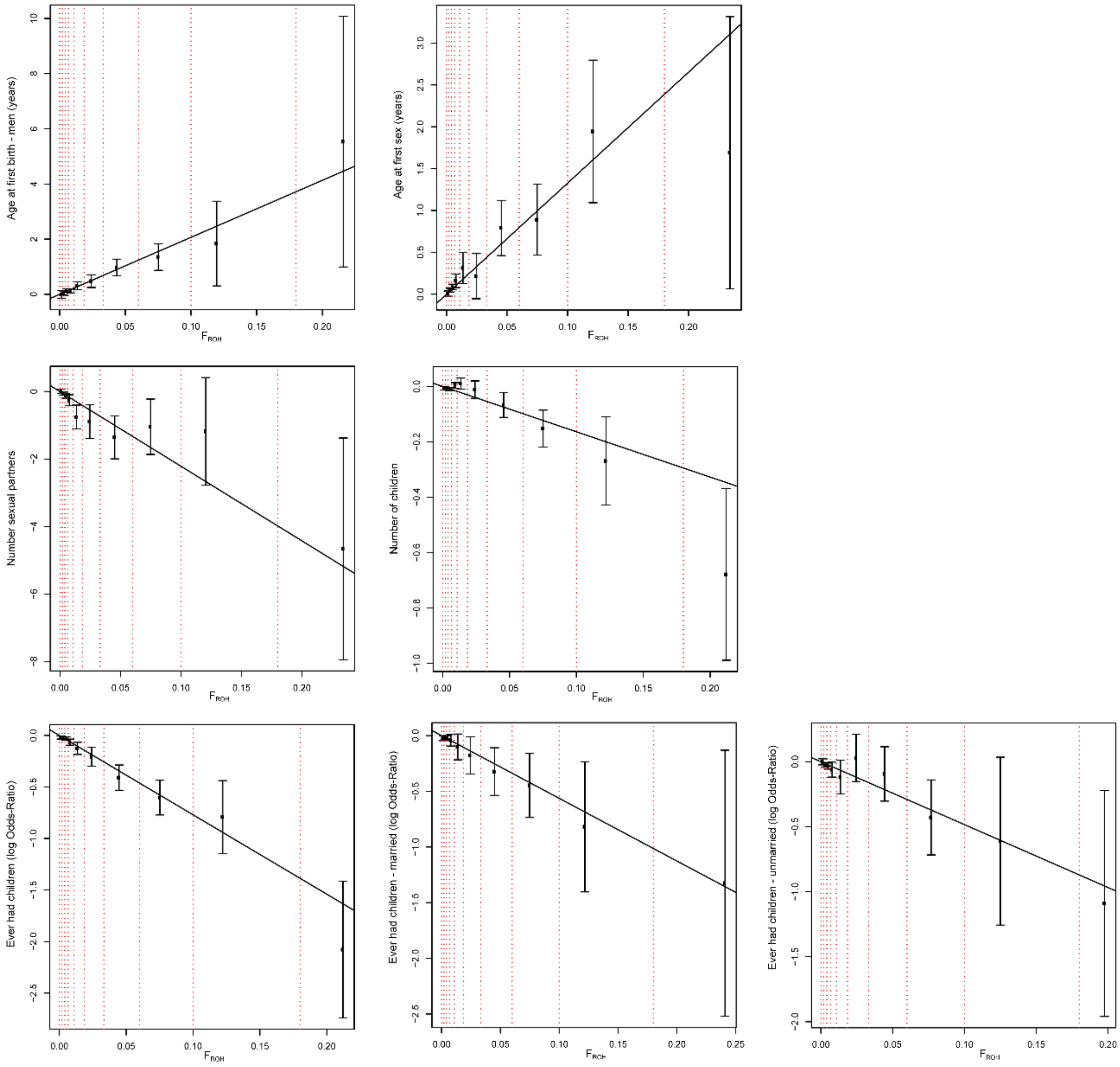
Age at first birth - men (years)
Age at first sex (years)
Number sexual partners
Number of children
Ever had children (log Odds-Ratio)
Ever had children - married (log Odds-Ratio)
Ever had children - unmarried (log Odds-Ratio)
F_ROH

9d

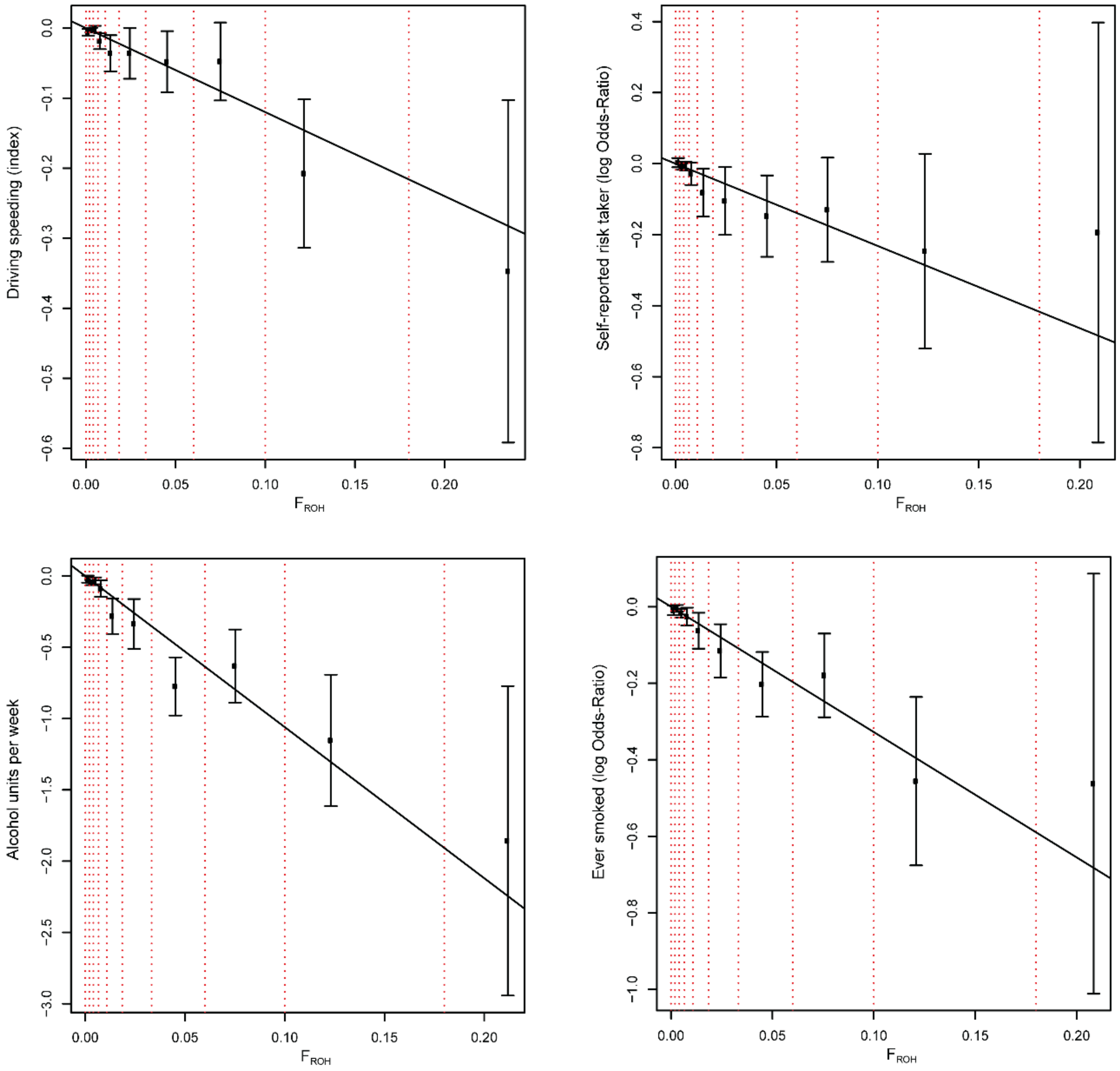

Driving speeding (index)
F_{ROH}
Self-reported risk taker (log Odds-Ratio)
F_{ROH}
Alcohol units per week
F_{ROH}
Ever smoked (log Odds-Ratio)
F_{ROH}

9e

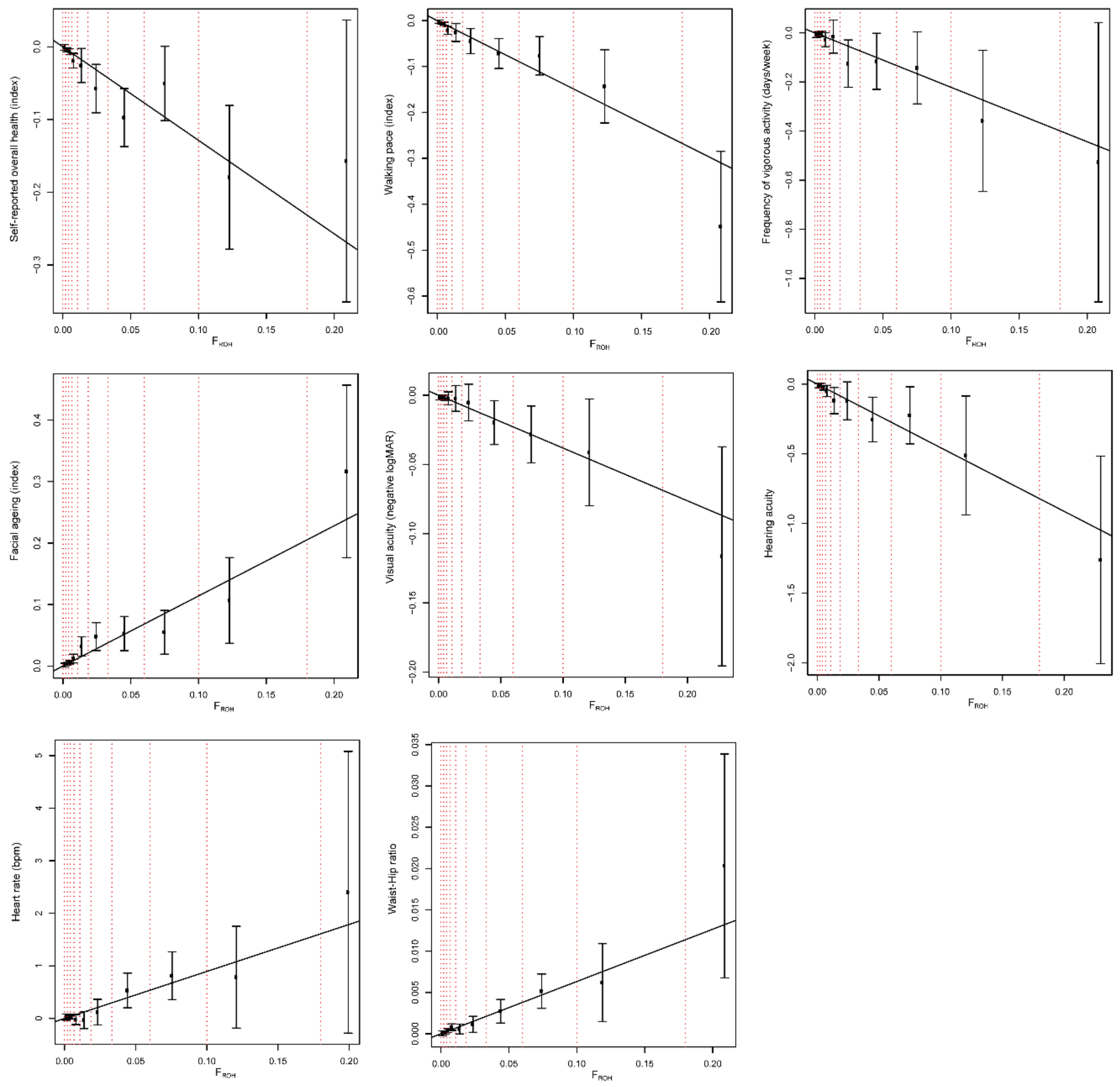
Self-reported overall health (index)
Walking pace (index)
Frequency of vigorous activity (days/week)
Facial ageing (index)
Visual acuity (negative logMAR)
Hearing acuity
Heart rate (bpm)
Waist-Hip ratio
F_{ROH}

9f

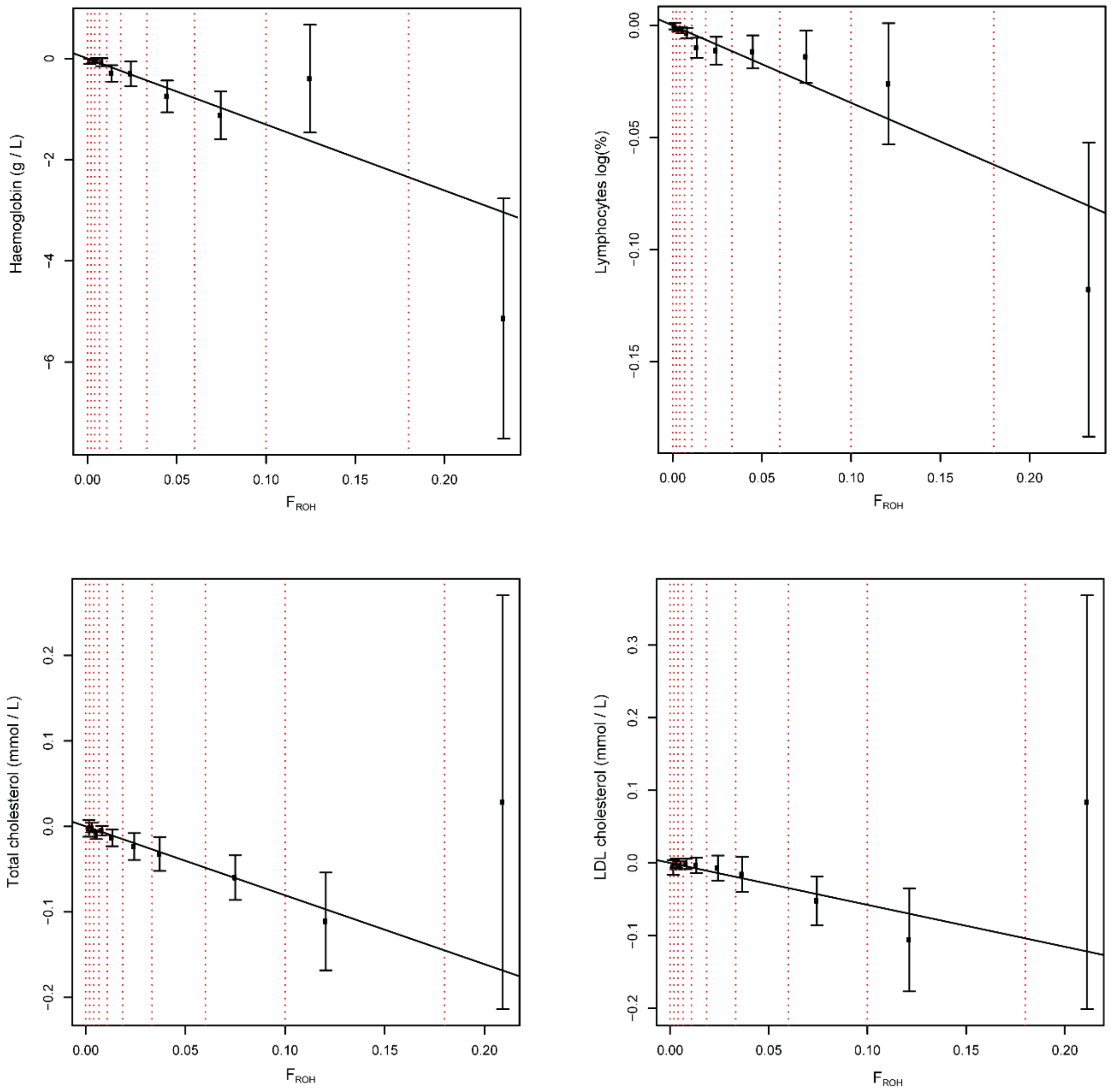

Supplementary Figure 9: Significant traits show a dosed response to increasing F_{ROH}. For all traits that reach experiment-wise significance in the meta-analysis, mean trait residuals are plotted in bins of increasing F_{ROH} (Methods) as also shown for height and Ever had children in Fig. 5a, b. Traits have been grouped into six categories (**a) Anthropometry, (b) Cognition, (c) Reproduction, (d) Risk-taking behaviour, (e) Well-being/Frailty, (f) Blood.** Although significant heterogeneity is observed for three traits (Height heterogeneity *p*-value = 7 x 10^{-8}, Educational Attainment heterogeneity *p*-value = 2 x 10^{-8}, Number ever born heterogeneity *p*-value = 7 x 10^{-5}) there is otherwise a dosed repose to increasing F_{ROH} for all traits. A dosed response across a wide range of F_{ROH} would be expected of a causal genetic effect, but not necessarily of environmental confounding. Although the effect of a confounder on a trait may be proportional, there is no a priori reason to expect a linear association between any confounder and F_{ROH}, especially extending to the large effects seen in very high F_{ROH} samples (F_{ROH}>0.18). All errors bars represent 95% confidence intervals.

10a

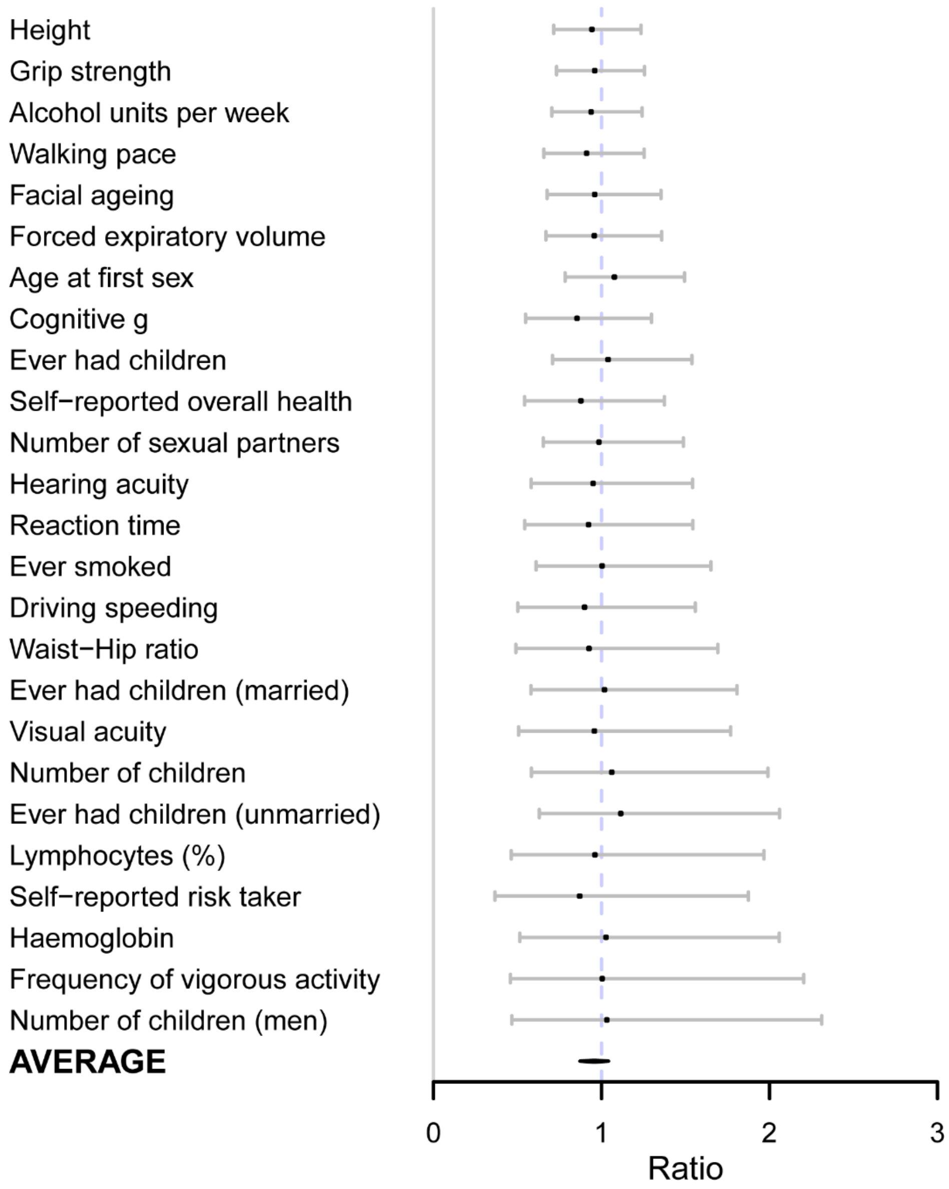
Height
Grip strength
Alcohol units per week
Walking pace
Facial ageing
Forced expiratory volume
Age at first sex
Cognitive g
Ever had children
Self−reported overall health
Number of sexual partners
Hearing acuity
Reaction time
Ever smoked
Driving speeding
Waist−Hip ratio
Ever had children (married)
Visual acuity
Number of children
Ever had children (unmarried)
Lymphocytes (%)
Self−reported risk taker
Haemoglobin
Frequency of vigorous activity
Number of children (men)
AVERAGE
0
1
2
3
Ratio

10b

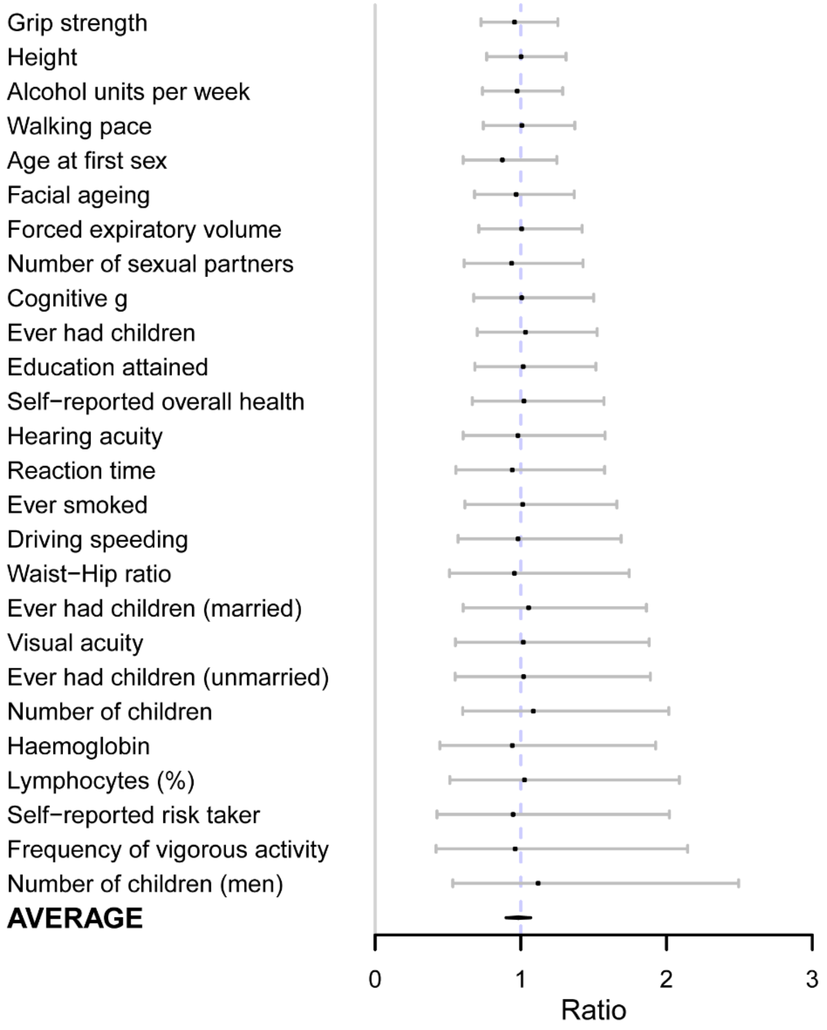

Supplementary Figure 10: Effect of fitting potential confounders as covariates. (a) Educational Attainment. For all traits that reach significance in UK Biobank, the ratio of effect size estimates with Educational Attainment (EA) fitted as an additional covariate ($\beta_{F_{\mathrm{ROH}}}^{+\mathrm{EA}}$) to the corresponding effect size estimates without EA ($\beta_{F_{\mathrm{ROH}}}$) are shown. The largest change is seen in Cognition (g) where fitting EA reduces $\beta_{F_{\mathrm{ROH}}}$ by 14.6%. However, since F_{ROH} is known to directly influence both g and EA, this change is not necessarily evidence of non-genetic effects. Overall fitting EA reduces the magnitude of $\beta_{F_{\mathrm{ROH}}}$ for 16 traits, but increases it for 9 traits, including number and likelihood of having children. **(b) Religious participation.** For the same traits (plus EA), the ratio of effect size estimates with a measure of religious participation (see Methods) fitted as an additional covariate ($\beta_{F_{\mathrm{ROH}}}^{+\mathrm{R}}$) to the corresponding effect size estimates without religious participation ($\beta_{F_{\mathrm{ROH}}}$) are shown. The largest reductions in $\beta_{F_{\mathrm{ROH}}}$ are seen for age at first sex (-12.7%) and number of sexual partners (-6.2%), suggesting that these traits may be partially confounded by social associations between F_{ROH} and religious beliefs. However, overall, fitting religious participation as a covariate increases $\beta_{F_{\mathrm{ROH}}}$ for 14 of 26 traits, again including number and likelihood of having children. All errors bars represent 95% confidence intervals.

11a

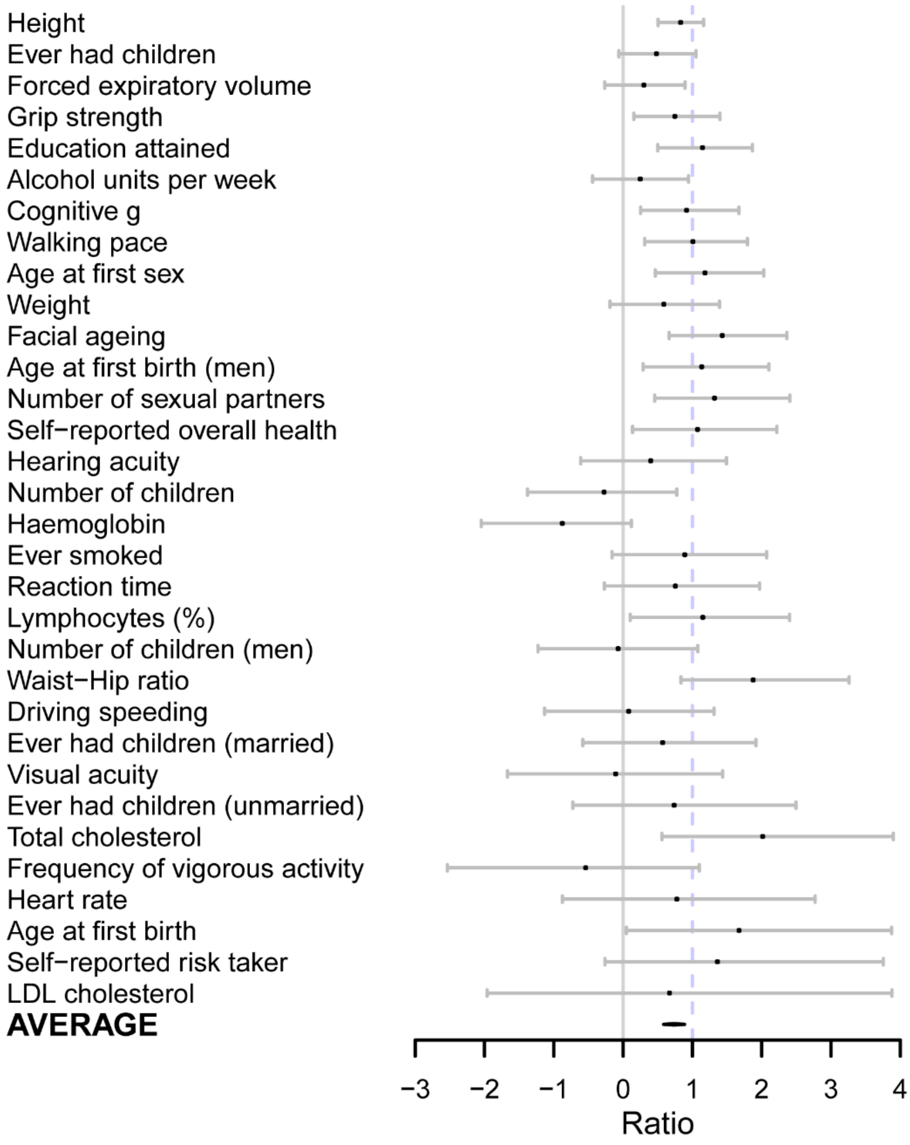
Height
Ever had children
Forced expiratory volume
Grip strength
Education attained
Alcohol units per week
Cognitive g
Walking pace
Age at first sex
Weight
Facial ageing
Age at first birth (men)
Number of sexual partners
Self−reported overall health
Hearing acuity
Number of children
Haemoglobin
Ever smoked
Reaction time
Lymphocytes (%)
Number of children (men)
Waist−Hip ratio
Driving speeding
Ever had children (married)
Visual acuity
Ever had children (unmarried)
Total cholesterol
Frequency of vigorous activity
Heart rate
Age at first birth
Self−reported risk taker
LDL cholesterol
AVERAGE
−3
−2
−1
0
1
2
3
4
Ratio

11b

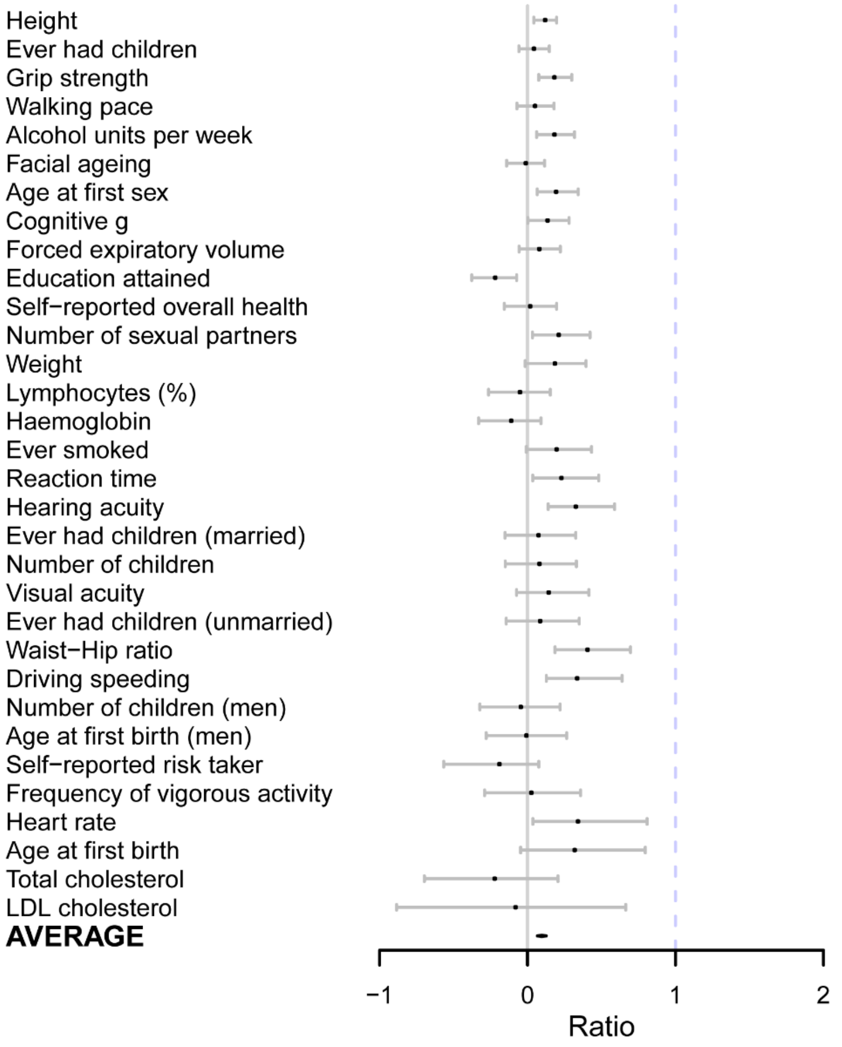

Supplementary Figure 11: Conditional effects of ROH < 5Mb and SNP homozygosity outside ROH. Multivariate models were run for all traits including 3 different measures for homozygosity: $F_{\text{SNP\_OusideROH}}$, $F_{\text{ROH<5Mb}}$ and $F_{\text{ROH>5Mb}}$ (See Methods). **(a) Relative effect of ROH < 5Mb**. The conditional effect of $F_{\text{ROH<5Mb}}$ divided by $\beta_{F_{\text{ROH}}}$ is shown for all significant traits. Across all traits, the meta-analysed average of this ratio is 0.74 [95% CI 0.59-0.89, *p*-value 5 x 10^{-22}, heterogeneity *p*-value 0.132]. ROH of length less than 5 Mb are believed to be largely unconfounded by recent consanguinity, supporting the hypothesis that environmental confounding is responsible for only a small fraction (approximately 25%) of the reported effects. **(b) Relative effect of SNP homozygosity outside ROH.** The conditional effect of $F_{\text{SNP\_OusideROH}}$ divided by $\beta_{F_{\text{ROH}}}$ is shown for all significant traits. Although there is some heterogeneity, as might be expected from different trait architectures, for all traits the effect of $F_{\text{SNP\_OusideROH}}$ is significantly less than the effect of F_{ROH}. Averaging across all traits, the meta-analysed average of $\beta_{F_{\text{SNP\_OutsideROH}}}:\beta_{F_{\text{ROH}}}$ is 0.12 [95% CI 0.04-0.20, *p*-value 2 x 10^{-10}, heterogeneity *p*-value 0.001], showing that ROH have a larger effect on inbreeding depression on complex traits than does common SNP homozygosity outside ROH. All errors bars represent 95% confidence intervals.

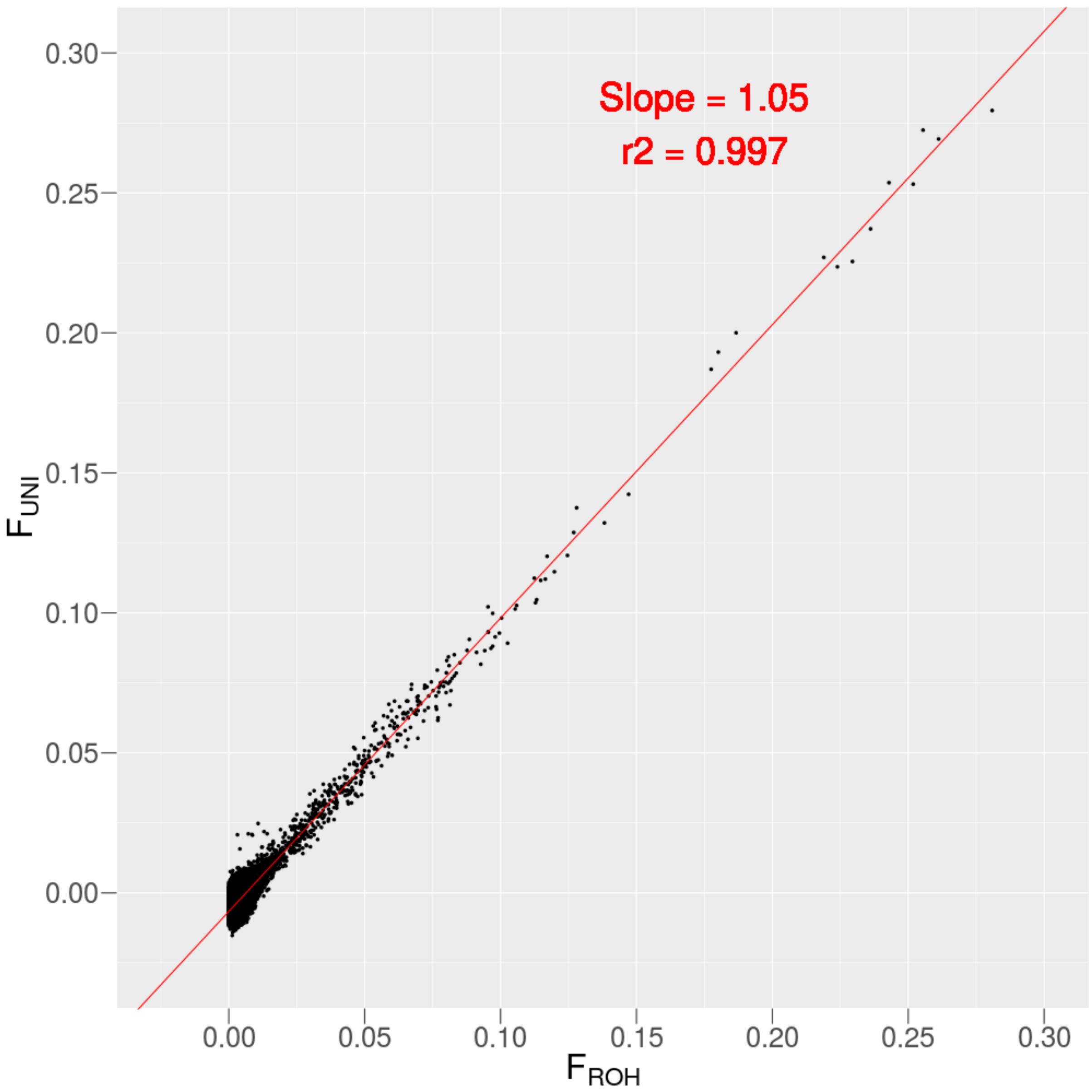

Supplementary Figure 12: Good correspondence between $\boldsymbol{F}_{\mathbf{UNI}}$ and $\boldsymbol{F}_{\mathbf{ROH}}$. For 141,774 British samples in UK Biobank F_{UNI}, calculated from excess homozygosity of imputed genotypes, is plotted against F_{ROH}, calculated from SNP array genotypes. A weighted linear regression line is shown in red. Because average inbreeding coefficients are low ($\bar{F}_{\mathrm{ROH}} = 0.003$ in this population), it is high F individuals who contribute most of the statistical power to estimates of β_F. Weighting the regression by an estimate of power contribution $(F_i^{\mathrm{ROH}} - \bar{F}_{\mathrm{ROH}})^2$, we estimate $\beta_{F_{\mathrm{UNI}},F_{\mathrm{ROH}}} = 1.05$ and $r^2 = 0.997$. The good correspondence between F_{UNI} and F_{ROH} suggests both have minimal bias in estimating F.

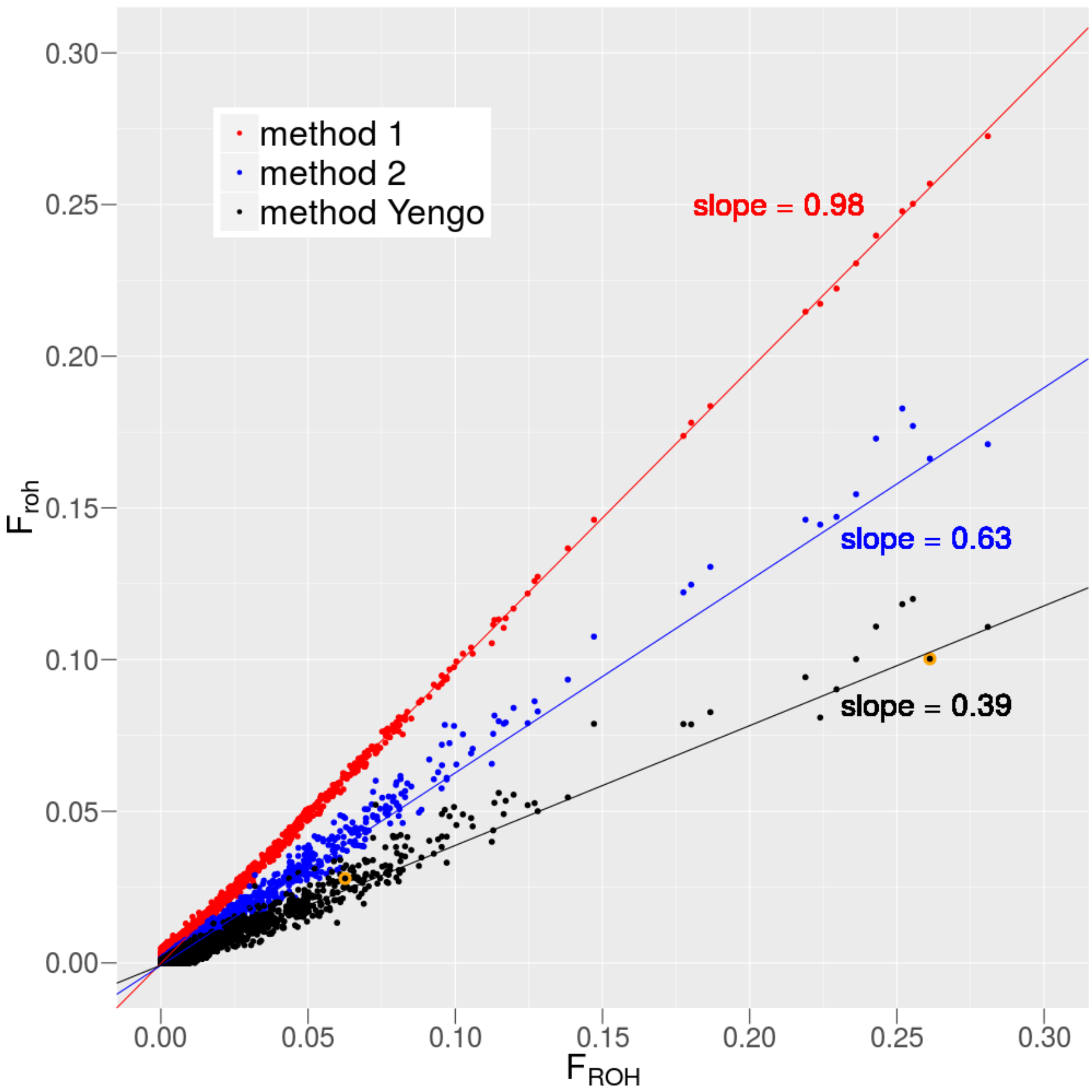

Supplementary Figure 13: Comparison of F_{roh} from imputed data and F_{ROH} from SNP array genotypes. For 141,774 British samples in UK Biobank, F_{roh} calculated from imputed genotypes is plotted against F_{ROH} calculated from SNP array genotypes for three methods of imputed genotype preparation. In method 1, in red, uncertain genotypes are removed. In method 2, in blue, uncertain genotypes are set to missing and in method Yengo, in black, no genotype filtering is performed. Increasingly permissive treatments of uncertain genotypes introduce increasing downward bias in F_{roh}. Two high F_{ROH} are highlighted in orange and further explored in Supplementary Figures 14a,b.

14a

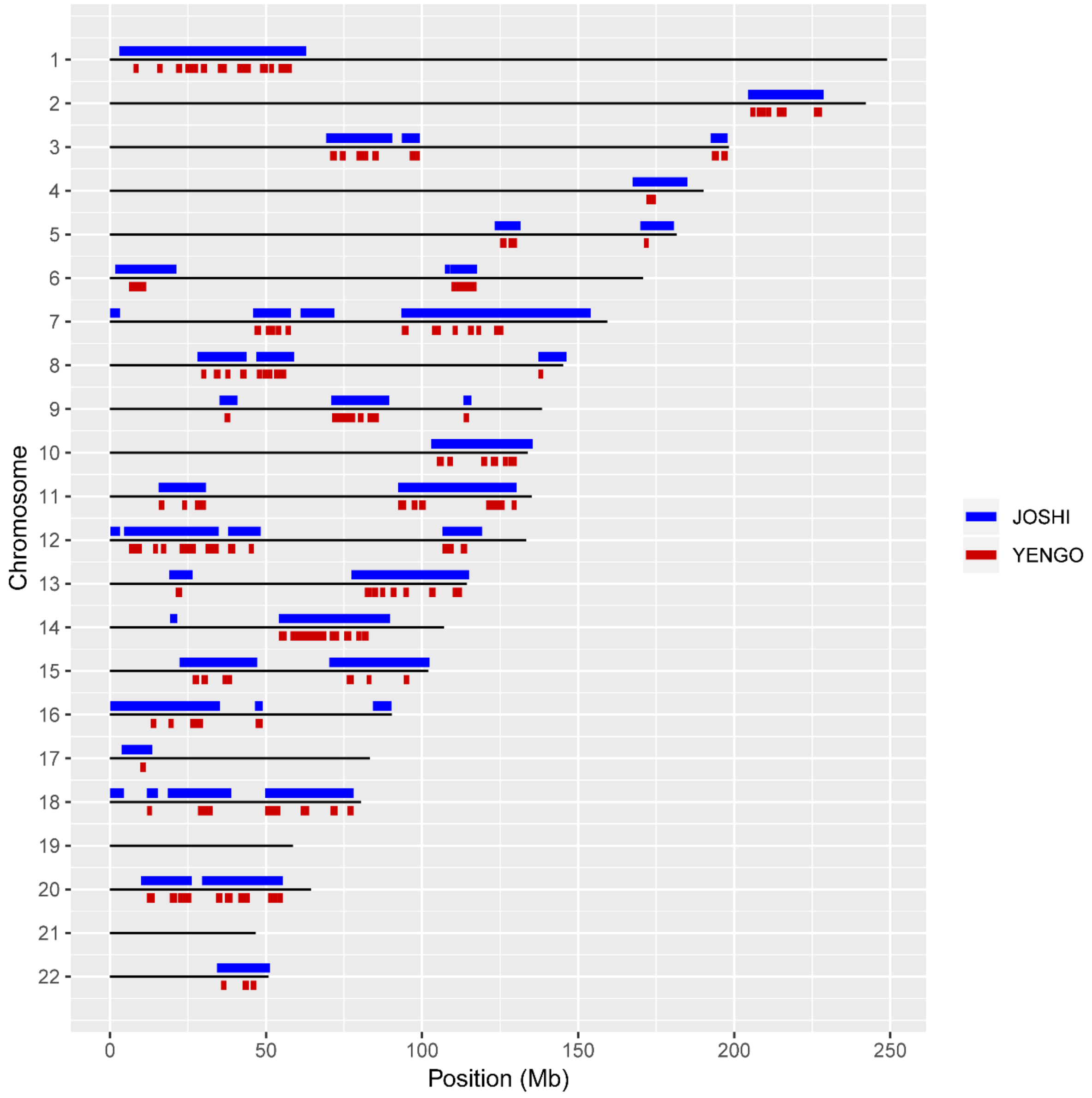

Chromosome
1
2
3
4
5
6
7
8
9
10
11
12
13
14
15
16
17
18
19
20
21
22
0
50
100
150
200
250
Position (Mb)
JOSHI
YENGO

14b

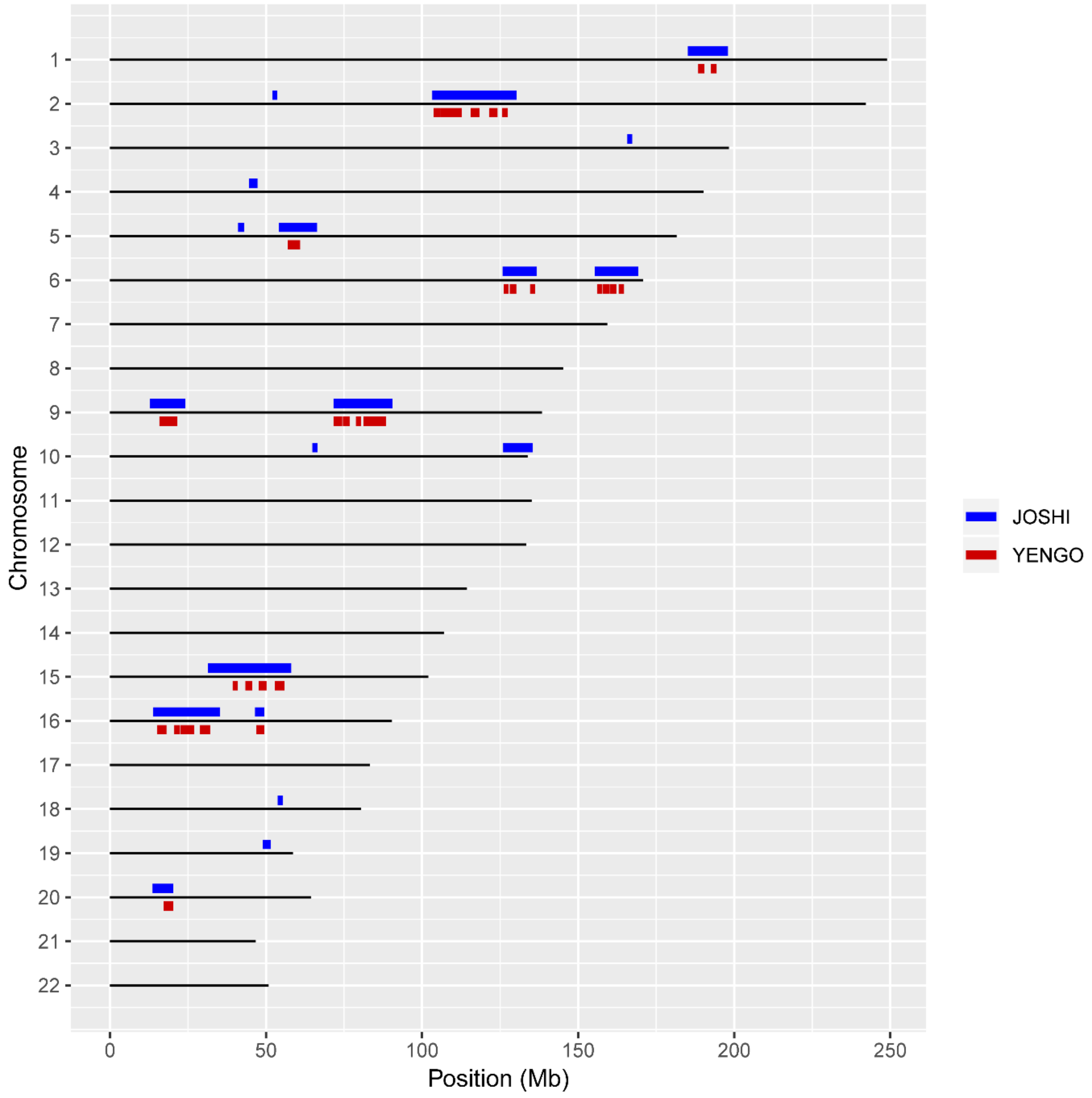

Supplementary Figure 14: Comparing ROH calling from SNP array genotypes and imputed dosages. For two high F_{ROH} individuals, the locations of called ROH are compared for two methods. The method shown in blue calls ROH from SNP array genotypes using the parameters used in Joshi et al. (2015). The method shown in red calls ROH from hard called imputed dosages following the method described in Yengo et al (2017). In both individuals the long ROH detected in SNP array genotypes, and which are thought to be autozygous segments, are broken up in the imputed data method by the presence of miscalled heterozygotes. **(a) Individual with $F_{\mathrm{ROH}} = 0.261$ thought to be offspring of first-degree relatives. (b) Individual with $F_{\mathrm{ROH}} = 0.0626$ thought to be offspring of third-degree relatives.**

15a

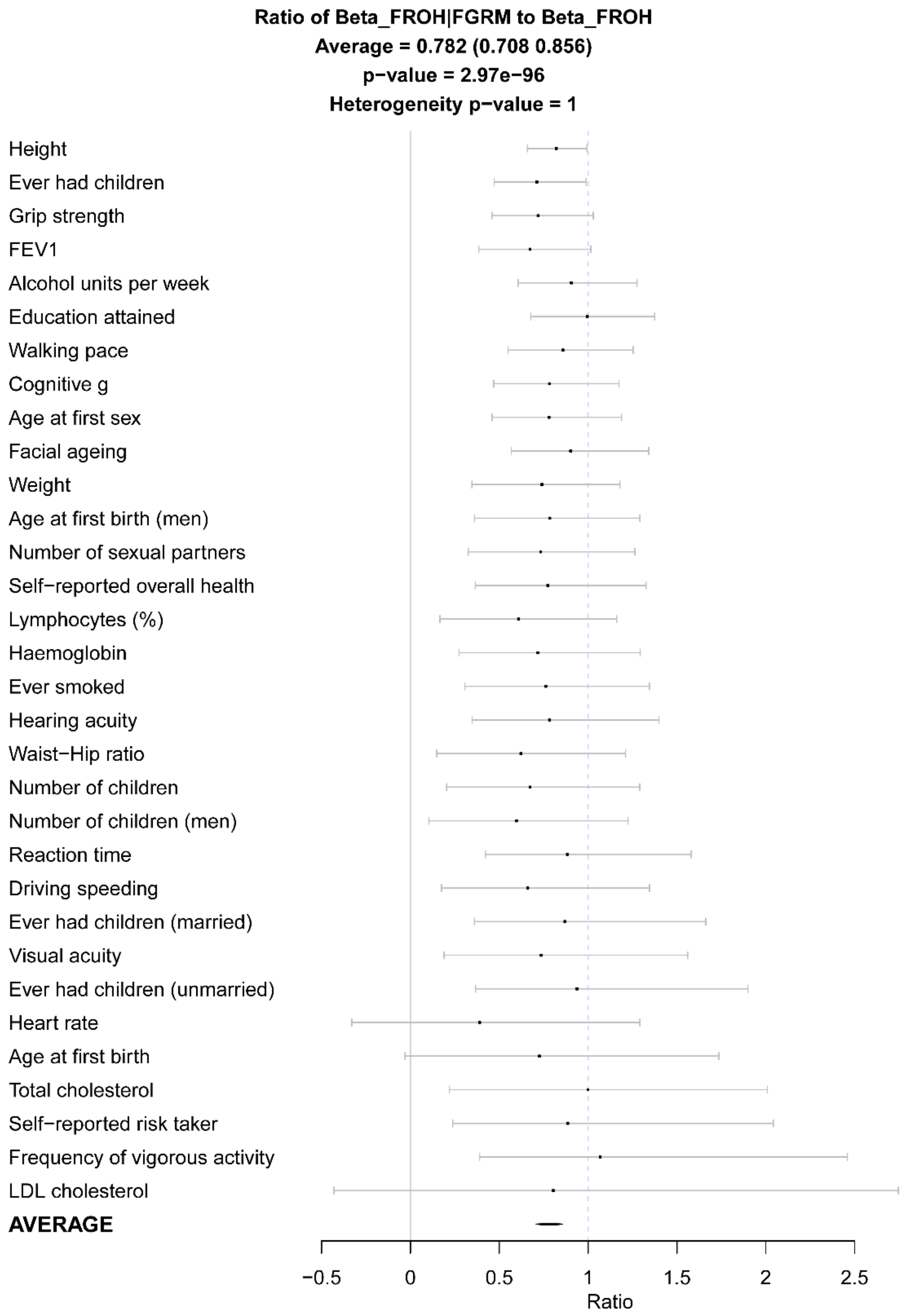
Ratio of Beta_FROH|FGRM to Beta_FROH
Average = 0.782 (0.708 0.856)
p−value = 2.97e−96
Heterogeneity p−value = 1
Height
Ever had children
Grip strength
FEV1
Alcohol units per week
Education attained
Walking pace
Cognitive g
Age at first sex
Facial ageing
Weight
Age at first birth (men)
Number of sexual partners
Self−reported overall health
Lymphocytes (%)
Haemoglobin
Ever smoked
Hearing acuity
Waist−Hip ratio
Number of children
Number of children (men)
Reaction time
Driving speeding
Ever had children (married)
Visual acuity
Ever had children (unmarried)
Heart rate
Age at first birth
Total cholesterol
Self−reported risk taker
Frequency of vigorous activity
LDL cholesterol
AVERAGE
−0.5
0
0.5
1
1.5
2
2.5
Ratio

15b

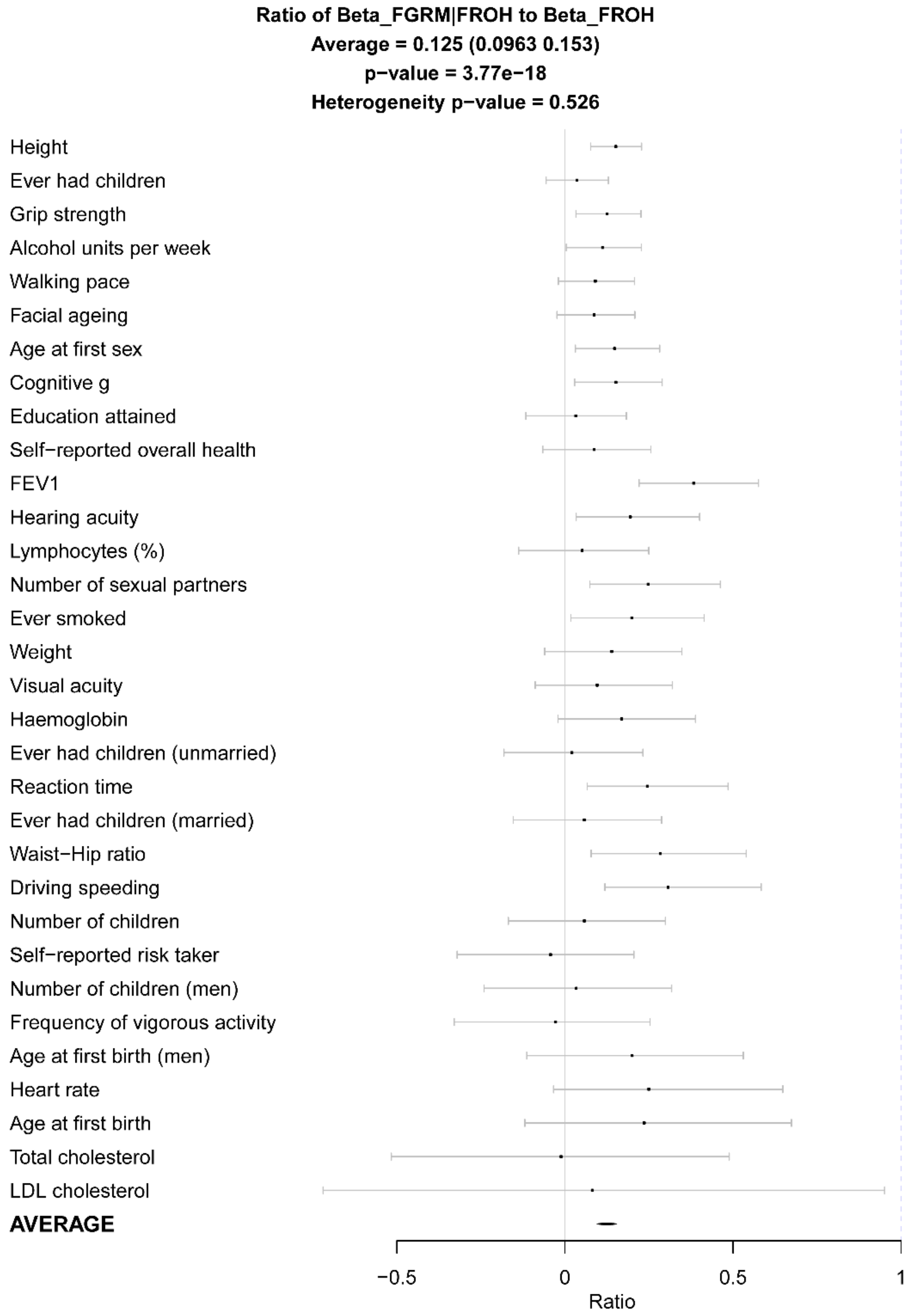

Supplementary Figure 15: Comparing effect estimates from bivariate models to the equivalent univariate estimates. For all significant traits, effect estimates were obtained from bivariate models of $Trait \sim F_{\mathrm{ROH}} + F_{\mathrm{GRM}}$ and compared to univariate estimates from the model $Trait \sim F_{\mathrm{ROH}}$. **(a) Ratio of $\boldsymbol{\beta}_{F_{\mathrm{ROH}}|F_{\mathrm{GRM}}}$ to $\boldsymbol{\beta}_{F_{\mathrm{ROH}}}$.** For all significant traits the ratio $\frac{\beta_{F_{\mathrm{ROH}}|F_{\mathrm{GRM}}}}{\beta_{F_{\mathrm{ROH}}}}$ is plotted. A meta-analysis across all traits gives an average ratio of 0.78 [95% CI 0.71-0.86]. **(b) Ratio of $\boldsymbol{\beta}_{F_{\mathrm{GRM}}|F_{\mathrm{ROH}}}$ to $\boldsymbol{\beta}_{F_{\mathrm{ROH}}}$.** For all significant traits the ratio $\frac{\beta_{F_{\mathrm{GRM}}|F_{\mathrm{ROH}}}}{\beta_{F_{\mathrm{ROH}}}}$ is plotted. A meta-analysis across all traits gives an average ratio of 0.12 [95% CI 0.10-0.15]. All errors bars represent 95% confidence intervals.

16a

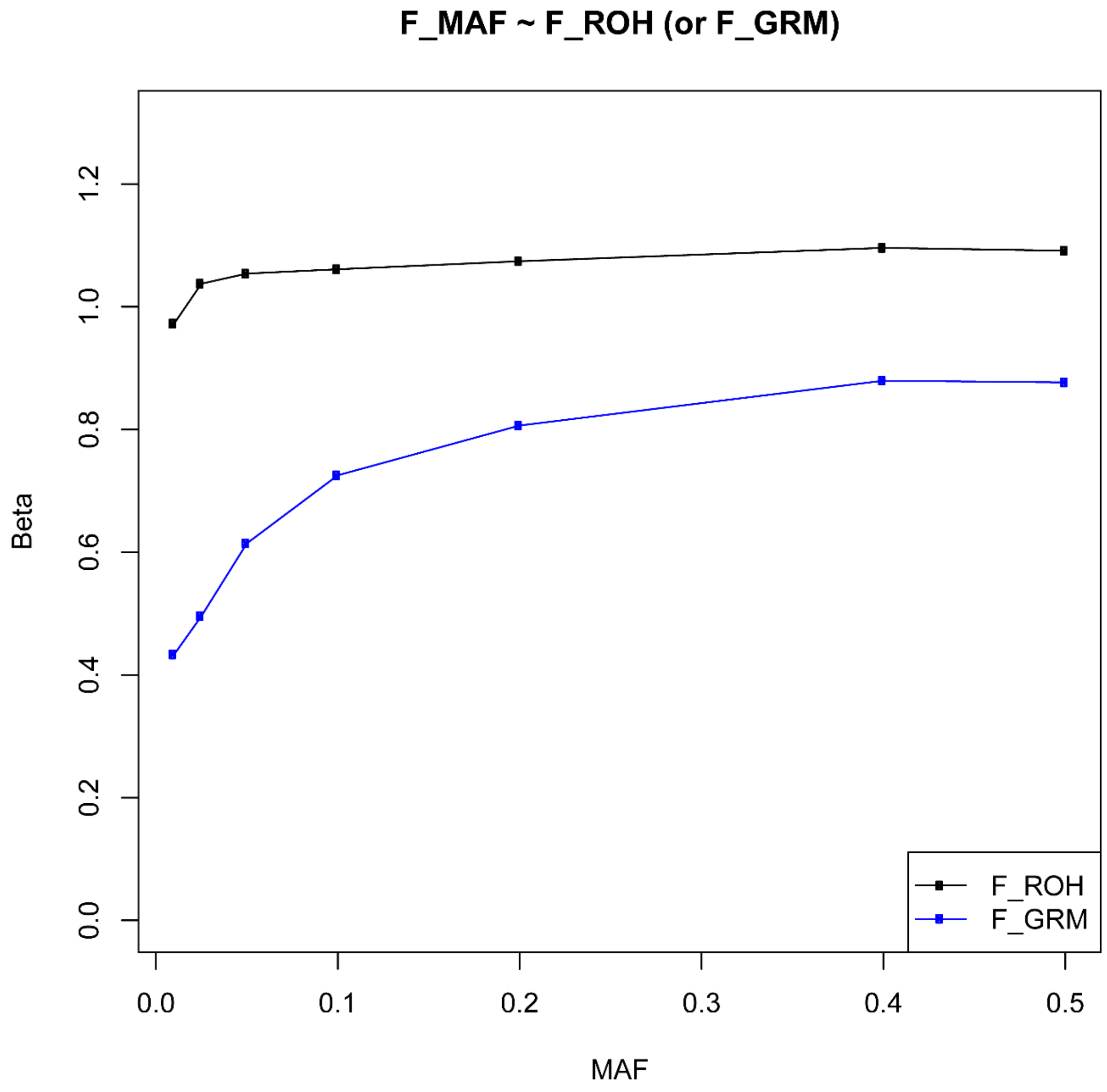

F_MAF ~ F_ROH (or F_GRM)
Beta
0.0
0.2
0.4
0.6
0.8
1.0
1.2
0.0
0.1
0.2
0.3
0.4
0.5
MAF
F_ROH
F_GRM

16b

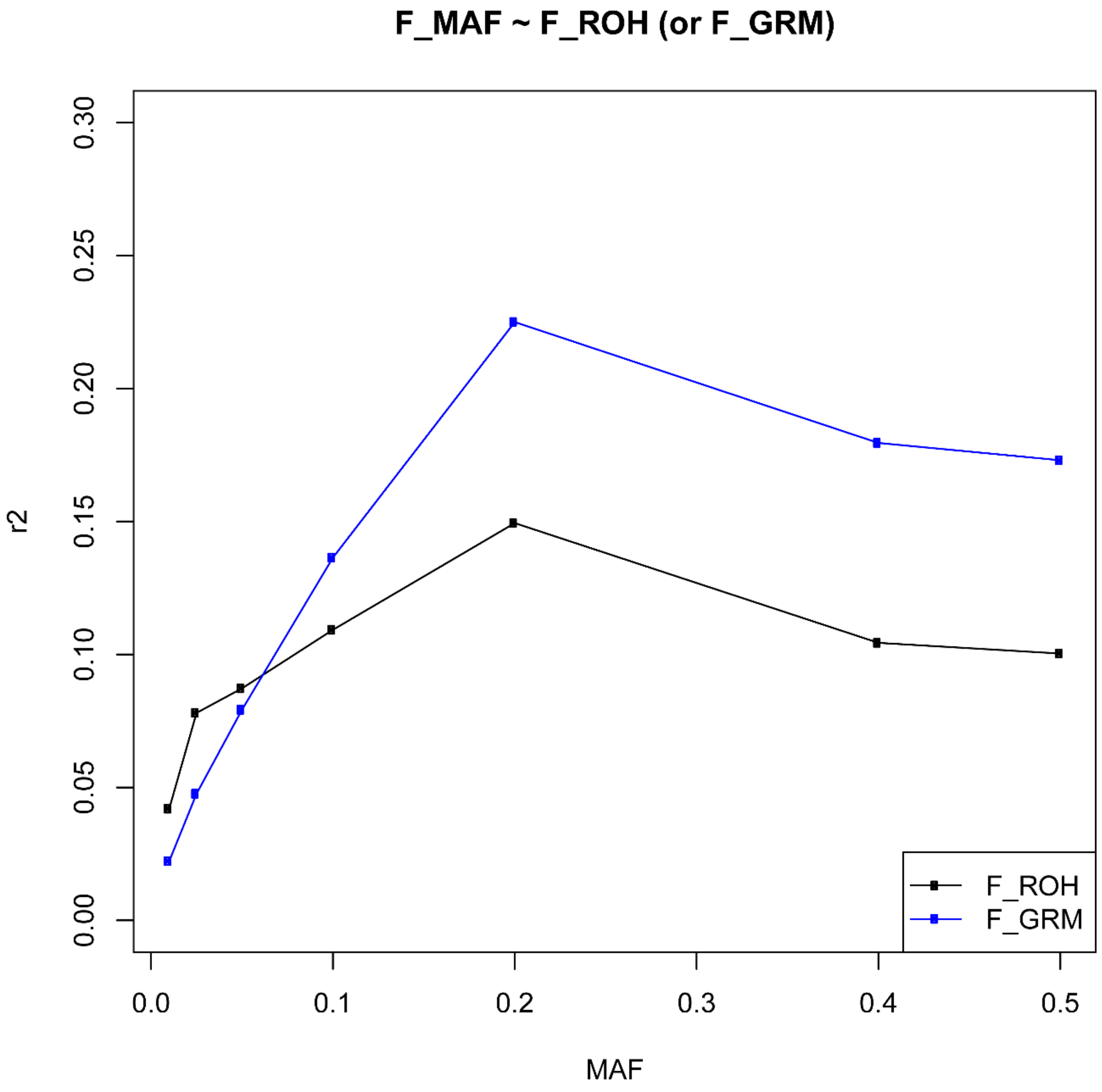

Supplementary Figure 16: Univariate relationships between estimates of inbreeding coefficient ($\boldsymbol{F_{\mathrm{ROH}}}$, $\boldsymbol{F_{\mathrm{GRM}}}$) and the excess homozygosity at specific allele frequencies. The excess homozygosity of SNPs at seven allele frequencies (F_{MAF}) was calculated for 402,559 genetically British samples in the phase 2 UKB imputation. **(a) Effect estimates of $\boldsymbol{F_{\mathrm{ROH}}}$ and $\boldsymbol{F_{\mathrm{GRM}}}$ on $\boldsymbol{F_{\mathrm{MAF}}}$.** Univariate models of $F_{\mathrm{MAF}} \sim F_{\mathrm{ROH}}$ and $F_{\mathrm{MAF}} \sim F_{\mathrm{GRM}}$ were fitted at each allele frequency. A one unit increases in F_{ROH} is associated with a one unit increase in F_{MAF} across all allele frequencies. In contrast, the slope of $\beta_{F_{\mathrm{MAF}}, F_{\mathrm{GRM}}}$ is downwardly biased at all allele frequencies. **(b) Correlations between of $\boldsymbol{F_{\mathrm{ROH}}}$, $\boldsymbol{F_{\mathrm{GRM}}}$ and $\boldsymbol{F_{\mathrm{MAF}}}$.** Univariate models of $F_{\mathrm{MAF}} \sim F_{\mathrm{ROH}}$ and $F_{\mathrm{MAF}} \sim F_{\mathrm{GRM}}$ were fitted at each allele frequency. Despite the downward bias of its effect estimate, F_{GRM} is more strongly correlated with F_{MAF} at most allele frequencies.

SUPPLEMENTARY TABLES

Supplementary Table 1: Genetic correlations between risk and reproductive traits. Genetic correlations estimated in UKB by LD score regression and their corresponding *p*-values. 5 reproductive traits and 4 risk traits are shown. The sign of age at first sex has been reversed so that larger trait values are associated with higher reproductive output. Positive correlations are shown in blue, with darker shades signifying Bonferroni-corrected significance. Unsurprisingly, positive genetic correlations are found within the groups of risk and reproductive success but, perhaps more unexpectedly, the genetic correlations between risk and reproductive traits are also most often positive. This is particularly true for smoking and self-declared risk taking.

		Ever had children	Number of children	***Earlier*** age at first sex	Number of sexual partners	Alcohol units	Ever smoked	Driving speed
Number of children	Rg	0.93						
	P	0						
Earlier age at first sex	Rg	0.57	0.53					
	P	3E-175	2E-200					
Number of sexual partners	Rg	0.08	0.10	0.52				
	P	1E-02	1E-03	2E-168				
Alcohol units	Rg	0.15	0.11	0.14	0.37			
	P	5E-02	1E-01	1E-02	7E-07			
Ever smoked	Rg	0.27	0.28	0.60	0.49	0.37		
	P	4E-26	2E-30	0	1E-119	3E-08		
Driving speed	Rg	0.00	0.03	0.01	0.29	0.2192	0.14	
	P	1	0.40	0.80	8E-24	2E-03	3E-07	
Self-declared risk taking	Rg	0.23	0.27	0.42	0.57	0.28	0.33	0.39
	P	5E-11	2E-16	9E-77	2E-137	3E-04	5E-46	3E-41

Supplementary Table 2: Number of SNPs extracted from UKB imputation by allele frequency. The excess homozygosity of SNPs at seven allele frequencies (F_{MAF}) was calculated for 402,559 genetically British samples in the phase 2 UKB imputation. The number of SNPs used at each frequency is shown.

MAF	Number of SNPs used in calculation of F_MAF
0.01	84835
0.025	122498
0.05	198310
0.1	261504
0.2	369777
0.4	253826
0.5	301191

SUPPLEMENTARY NOTE 1: Trait descriptions

45 quantitative traits were initially chosen for analysis in a potentially wide range of cohorts from the ROHgen consortium. During the initial meta-analysis of these traits, the full release of >500,000 samples from UK Biobank (UKB) became available and, it was decided to include a further 55 less-commonly measured traits available in UKB. Of these new traits, 21 were binary, requiring an extension to the existing analysis plan. 7 of the UKB traits were also measured in some ROHgen cohorts and were thus analysed in a subset of ROHgen cohorts willing to rerun the new analysis plan. In summary, a total of 100 complex traits were analysed; 45 in a potentially wide range of ROHgen cohorts, 7 in a subset of ROHgen cohorts and 48 in UKB only. All are defined below, under headings in the format **short name – full name – units**.

afb - Age at first birth – years. Age of subject (either male or female) when their first child was born. Nulliparous samples and reported ages less than 12 or greater than 80 were excluded.

afb_men - Age at first birth (men) – years. Men only. Unlike most other traits, age at first birth was treated as a separate trait for men and women, and the full set of analyses was therefore performed on both sexes separately. Age at first birth (men) is the age of a male subject when their first child was born. Nulliparous samples and reported ages less than 12 or greater than 80 were excluded.

afb_women - Age at first birth (women) – years. Women only. Unlike most other traits, age at first birth was treated as a separate trait for men and women, and the full set of analyses was therefore performed on both sexes separately. Age at first birth (women) is the age of a female subject when their first child was born. Nulliparous samples and reported ages less than 12 or greater than 60 were excluded.

age_menarche - Age at menarche – years. Women only. Reported age at menarche. Women with age at menarche less than 5 or greater than 25 were excluded.

age_menopause - age at menopause (years). Women only. Age at natural menopause. Women whose menopause was due to surgical operations (hysterectomy/ovariectomy), cancer treatment

(radiation, chemotherapy) or on HRT before menopause were excluded. Responses below 35 or greater than 70 were also excluded.

birth_weight – Birth weight – kg. Individual's own weight at birth. Participants who were known to be part of a multiple birth (twins, triplets, etc.) were set to NA. Values less than 0.5 kg or greater than 7 kg were excluded.

bmi – Body mass index – kgm^{-2}. Weight in kilograms divided by height in metres squared. Values less than 10 or greater than 150 were excluded.

dp_dia – Diastolic blood pressure – mmHg. Averaged readings taken during a single session. Guidance was to take the unweighted mean of second and third readings although cohorts were given discretion to use best judgement where appropriate. Participants known to be on hypertension medication had 10mmHg added to their readings. Values less than 20 or greater than 200 were excluded.

bp_sys – Systolic blood pressure – mmHg. Averaged readings taken during a single session. Guidance was to take the unweighted mean of second and third readings although cohorts were given discretion to use best judgement where appropriate. Participants known to be on hypertension medication had 15mmHg added to their readings. Values less than 50 or greater than 300 were excluded.

edu – Education Attained – years. Based on SSGAC, Education Attained was defined in accordance with the ISCED 1997 classification(UNESCO), relating to seven categories of educational attainment that are internationally comparable. Subjects age <30 were excluded as were values $\notin$ {1,7,10,13,15,19,22}.

Definition	US years of schooling
Pre-primary education	1
Primary education or first stage of basic education	7
Lower secondary or second stage of basic education	10
(Upper) secondary education	13
Post-secondary non-tertiary education	15
First stage of tertiary education (not leading directly to an advanced research qualification)	19
Second stage of tertiary education (leading directly to an advanced research qualification e.g. PhD)	22

fev1 – Forced expiratory volume in 1 second – Litres. Where multiple blows were available the maximum valid reading was used. Values less than 0 or greater than 10 were excluded.

fev1perfvc – Forced expiratory volume in 1 second / forced vital capacity – no units. Values less than 0 or greater than 15 were excluded.

fpg – Fasting plasma glucose – mmolL^{-1}. Known diabetic subjects were excluded, as were subjects with fpg > 7 or hba1c > 6.5. Measurements made in whole blood (not plasma) were multiplied by 1.13 to estimate fpg.

g – Cognitive g – z-score. The first unrotated principal component of three or more tests of different domains of cognition. Care was taken to ensure this was in the direction of larger values being associated with greater cognition. Specifically, the sign of the correlation between Cognitive g and

Education attained was ensured to be positive in all cohorts. This trait was rank-normalised and values less than -10 or greater than 8 were excluded.

hb – Haemoglobin – gL^{-1}. Concentration of haemoglobin. Values less than 0 or greater than 500 were set to NA.

hba1c – Glycosylated haemoglobin – % of hb (DCCT)[2]. Set to NA for known diabetics and all subjects for whom HbA1c > 6.5 or fpg > 7. Also, set to NA for subjects with known major blood abnormalities (thalassaemia, sickle cell anaemia, etc.), subjects who have had a blood transfusion in the previous 3 months.

hdl – High-density lipoprotein cholesterol – $mmolL^{-1}$. Taken only from fasted or semi-fasted subjects. If semi-fasted a covariate specifying fasting time was required. Values greater than 5.17 were set to NA.

height – Height – meters. Standing height in meters. Values less than 1.2 or greater than 2.5 were set to NA.

hr – Heart rate – beats per minute. Participants on cardiac medications (Beta blockers, antiarrhythmics) were exclude as were those with previous myocardial infarction or heart failure. Values less than 20 or greater than 150 were set to NA.

ldl – Low-density lipoprotein cholesterol – $mmolL^{-1}$. Taken only from fasted or semi-fasted subjects. If semi-fasted a covariate specifying fasting time was required. If HDL cholesterol, total cholesterol and Triglycerides were all provided, LDL cholesterol was calculated using Friedewald's equation. Alternatively, LDL cholesterol could be supplied if directly measured. Samples known to be on lipid lowering medication were adjusted by dividing by a factor of 0.7. Values less than 0 or greater than 10.34 were set to NA.

log.egfr – Estimated glomerular filtration rate – $mLmin^{-1}1.73m^{-2}$. Glomerular filtration rate was estimated from measured creatinine (in $mgdL^{-1}$) using the formula $186*creatinine^{-1.154}*age^{-0.203}$. In cohorts with African or African-American ancestry these values were multiplied by a correction factor of 1.21. Values of creatinine greater than 20 or eGFR greater than 200 were set to NA.

log.fast_ins – Fasting insulin – $pmolL^{-1}$. Known diabetic samples, as well as samples with fpg > 7 or HbA1c < 6.5 were excluded. Values of fasting insulin greater than 1000 were set to NA.

log.fibrinogen – Fibrinogen – gL^{-1}. Plasma fibrinogen levels. Values greater than 20 were set to NA.

log.hscrp – high sensitivity C-reactive protein – $nmolL^{-1}$. Serum levels of C-reactive protein (CRP) detected with high sensitivity systems (lower detection limit around 1 $nmolL^{-1}$). Samples known to be on anti-inflammatory drugs (ATC codes L01, L03, L04, L02A, L02B) were set to NA, as were values greater than 952 $nmolL^{-1}$.

log.il6 – Interleukin-6 – $pgmL^{-1}$. Serum levels of Interleukin-6. Samples known to be on anti-inflammatory drugs (ATC codes L01, L03, L04, L02A, L02B) were set to NA, as were values greater than 100 $pgmL^{-1}$.

log.lymphoc – Lymphocytes – %. Percentage of lymphocytes per white blood cell count. Values greater than 100 were set to NA.

log.mpv – Mean platelet volume – fL. Mean platelet volume in femtolitres. Values greater than 30 were set to NA.

log.tnfa – Tumour necrosis factor alpha – pgmL^{-1}. Samples known to be on anti-inflammatory drugs (ATC codes L01, L03, L04, L02A, L02B) were set to NA, as were values greater than 100 pgmL^{-1}.

log.triglyc – Triglycerides – mmolL^{-1}. Taken only from fasted or semi-fasted subjects. If semi-fasted a covariate specifying fasting time was required. Values of Triglycerides greater than 33.9 mmolL^{-1} were set to NA.

log.wbc – White blood cell count – 10^9 per Litre. Values greater than 30 x 10^9 per Litre were excluded.

log10.alt – Alanine transaminase – IUper Litre. Plasma concentrations of Alanine transaminase (also called Glutamic-pyruvate transaminase). Values greater than 500 IU per Litre were set to NA.

log10.ggt – Gamma-Glutamyl Transferase – IU per Litre. Plasma concentrations of Gamma-Glutamyl Transferase. Values greater than 1000 IU per Litre were set to NA.

monoc – Monocytes - %. Percentage of monocytes in white blood cell count. Values greater than 40 were set to NA.

neb – Number ever born – count. Number of children the subject has brought into being. Subjects aged less than 45 were excluded from this analysis.

neb_men – Number ever born (men) – count. Men only. Unlike most other traits, number ever born was treated as a separate trait for men and women, and the full set of analyses was therefore performed on both sexes separately. Number ever born (men) is the number of children fathered by a male subject. Subjects aged less than 45 were excluded from this analysis.

neb_women – Number ever born (women) – count. Women only. Unlike most other traits, number ever born was treated as a separate trait for men and women, and the full set of analyses was therefore performed on both sexes separately. Number ever born (women) is the number of children given birth to by a female subject. Subjects aged less than 45 were excluded from this analysis.

plt – Platelet count - 10^9 per Litre. Platelet count in whole blood. Values less than 20 or greater than 1000 were set to NA.

pr – PR interval – ms. Electrocardiographic PR interval. Participants on cardiac medications (Beta blockers, antiarrhythmics) were exclude as were those with previous myocardial infarction or heart failure. Values less than 80 or greater than 320 were set to NA.

qrs – QRS duration – ms. Electrocardiographic QRS duration. Participants on cardiac medications (Beta blockers, antiarrhythmics) were exclude as were those with previous myocardial infarction or heart failure. Values less than 30 or greater than 120 were set to NA.

qt – QT interval – ms. Electrocardiographic QT interval. Participants on cardiac medications (Beta blockers, antiarrhythmics) were exclude as were those with previous myocardial infarction or heart failure. Values less than 200 or greater than 700 were set to NA.

ser – Spherical equivalent refraction – no units. The mean of left and right eyes calculated from spherical and cylindrical power of each eye by the standard formula ser = sphere + 0.5*cylinder. Samples known to have had eye surgery were set to NA, as were values less than -15 or greater than +15.

tot_chol – Total cholesterol – mmolL^{-1}. Taken only from fasted or semi-fasted subjects. If semi-fasted a covariate specifying fasting time was required. Samples known to be on lipid lowering medication were adjusted by dividing by a factor of 0.8. Values greater than 16.8 molL^{-1} were set to NA.

uric – Uric acid – umolL^{-1}. Serum urate concentration. Values greater than 1190 umolL^{-1} were set to NA.

weight – Weight – kg. Weight in kilograms. Values less than 20 kg or greater than 250 kg were set to NA.

whr – Waist : Hip ratio – no units. Calculated when both waist and hip circumference were available in centimetres. Values of waist or hip circumference less than 20 or greater than 300 were set to NA, as were values of waist : hip ratio less than 0.3 or greater than 2.

alcohol_units - Alcohol units per week – UK units per week. Self-declared alcohol consumption in UK units (10 ml of ethanol) per week. Where necessary this was estimated from alcohol intake frequency and average drink sizes. Values were capped to 100 units per week.

ever_married_glm – Ever married – TRUE/FALSE. Subjects who were known to be (or have been) married or in a long-term cohabiting relationship were encoded as 1 while all others were encoded as 0.

ever_smoked_glm – Ever smoked – TRUE/FALSE. Subjects who reported either being current smokers or having previously smoked on all or most days were encoded as 1, while those who had never or only occasionally smoked were encoded as 0.

neb_parous – Number ever born (parous) – count. In samples with one or more children, number ever born (parous) is equal to number of children ever born (neb). All other samples are set to NA.

parous_glm – Ever had children – TRUE/FALSE. For all samples with a non-missing value of number ever born (neb) a trait was defined with value 1 for samples with neb>0 and value 0 for samples with neb=0.

parous_married_glm – Ever had children (married) – TRUE/FALSE. Ever had children defined only for samples for whom ever married = 1. Set to NA for all samples where ever married is 0 or NA.

parous_unmarried_glm – Ever had children (unmarried) – TRUE/FALSE. Ever had children defined only for samples for whom ever married = 0. Set to NA for all samples where ever married is 1 or NA.

age_at_first_sex – Age at first sex – years. Response to the question *What was your age when you first had sexual intercourse?* Participants who declined to answer, or who gave an answer less than 3 were excluded. Participants who answered *Never had sex* were set to their current age. For more details see http://biobank.ctsu.ox.ac.uk/crystal/field.cgi?id=2139.

age_facial_hair – Relative age of facial hair – index. Men only. Response to the question *When did you start to grow facial hair?*. Participants were given five options: *Younger than average*, *About average age*, *Older than average*, *Do not know* and *Prefer not to answer* which were encoded -1, 0, 1, NA and NA respectively. For more details see http://biobank.ctsu.ox.ac.uk/crystal/field.cgi?id=2375.

age_voice_broke – Relative age voice broke – index. Men only. Response to the question *When did your voice break?*. Participants were given five options: *Younger than average*, *About average age*,

Older than average, *Do not know* and *Prefer not to answer* which were encoded -1, 0, 1, NA and NA respectively. For more details see http://biobank.ctsu.ox.ac.uk/crystal/field.cgi?id=2385.

alb – Age at last birth – years. Women only. Response to the question *How old were you when you had your LAST child?*. Responses less than 8 or greater than 65 were excluded. For more details see http://biobank.ctsu.ox.ac.uk/crystal/field.cgi?id=2764.

ankle_width – Mean ankle width – mm. Average of left and right ankle width as measured by the spacing between measurement transducer pads on each heel. For more details see http://biobank.ctsu.ox.ac.uk/crystal/field.cgi?id=4100.

any_pain_glm – Any reported pain – TRUE/FALSE. Participants were asked the question *In the last month have you experienced any of the following that interfered with your usual activities? (You can select more than one answer)* and given ten options: *Headache*, *Facial pain*, *Neck or shoulder pain*, *Back pain*, *Stomach or abdominal pain*, *Hip pain*, *Knee pain*, *Pain all over the body*, *None of the above* and *Prefer not to answer*. Participants who selected any of the first eight options were coded as 1, those who selected only *None of the above* were coded as 0 and the remainder were treated as NA. For more details see http://biobank.ctsu.ox.ac.uk/crystal/field.cgi?id=6159.

any_same_sex_glm – Any same-sex partners – TRUE/FALSE. Participants were asked the question *Have you ever had sexual intercourse with someone of the same sex?* and given the options *Yes*, *No* and *Prefer not to answer* which were encoded 1, 0 and NA respectively. For more details see http://biobank.ctsu.ox.ac.uk/crystal/coding.cgi?id=100352.

back_pain_glm – Backpain – TRUE/FALSE. Participants were asked the question *In the last month have you experienced any of the following that interfered with your usual activities? (You can select more than one answer)* and given ten options: *Headache*, *Facial pain*, *Neck or shoulder pain*, *Back pain*, *Stomach or abdominal pain*, *Hip pain*, *Knee pain*, *Pain all over the body*, *None of the above* and *Prefer not to answer*. Participants who selected *Back pain* were coded as 1, those who selected only *Prefer not to answer* were coded as NA and the remainder set to 0. For more details see http://biobank.ctsu.ox.ac.uk/crystal/field.cgi?id=6159.

baldness – Baldness pattern – index. Men only. Male participants were asked the question *Which of the following best describes your hair/balding pattern?* and shown four images of increasing hair loss (patterns 1 to 4). Responses were coded 1 to 4, where 4 represents most hair loss. For more details see http://biobank.ctsu.ox.ac.uk/crystal/field.cgi?id=2395.

body_pain_glm – Whole body pain – TRUE/FALSE. Participants were asked the question *In the last month have you experienced any of the following that interfered with your usual activities? (You can select more than one answer)* and given ten options: *Headache*, *Facial pain*, *Neck or shoulder pain*, *Back pain*, *Stomach or abdominal pain*, *Hip pain*, *Knee pain*, *Pain all over the body*, *None of the above* and *Prefer not to answer*. Participants who selected *Pain all over the body* were coded as 1, those who selected only *Prefer not to answer* were coded as NA and the remainder set to 0. For more details see http://biobank.ctsu.ox.ac.uk/crystal/field.cgi?id=6159.

broken_bones_glm – Broken bones – TRUE/FALSE. Participants were asked the question *Have you fractured/broken any bones in the last 5 years?* and given the options *Yes*, *No*, *Do not know* and *Prefer not to answer* which were encoded 1, 0, NA and NA respectively. For more details see http://biobank.ctsu.ox.ac.uk/crystal/field.cgi?id=2463.

cancer_diagnosis_glm – Cancer diagnosis – TRUE/FALSE. Participants were asked the question *Has a doctor ever told you that you have had cancer?* and given the options *Yes*, *No*, *Do not know* and

Prefer not to answer which were encoded 1, 0, NA and NA respectively. For more details see http://biobank.ctsu.ox.ac.uk/crystal/field.cgi?id=2453.

dead_glm – Dead – TRUE/FALSE. Death records in UKB are periodically updated by linkage to national death registries. At data download on 13/12/2017, 13739 participants had record dates of death and were thus encoded at 1. Those without a death register entry were encoded as 0. For more details see http://biobank.ctsu.ox.ac.uk/crystal/field.cgi?id=40000.

depression_glm – Self-reported mood disorder – TRUE/FALSE. Participants were asked the question *Have you ever seen a general practitioner (GP) for nerves, anxiety, tension or depression?* and given the options *Yes*, *No*, *Do not know* and *Prefer not to answer* which were encoded 1, 0, NA and NA respectively. For more details see http://biobank.ctsu.ox.ac.uk/crystal/field.cgi?id=2090.

diabetes_diagnosis_glm – Diabetes diagnosis – TRUE/FALSE. Participants were asked the question *Has a doctor ever told you that you have diabetes?* and given the options *Yes*, *No*, *Do not know* and *Prefer not to answer* which were encoded 1, 0, NA and NA respectively. For more details see http://biobank.ctsu.ox.ac.uk/crystal/field.cgi?id=2443.

facial_ageing – Facial ageing – index. Participants were asked the question *Do people say that you look:* and given the options *Younger than you are*, *Older than you are*, *About your age*, *Do not know* and *Prefer not to answer* which were encoded -1, 1, 0, NA and NA respectively. For more details see http://biobank.ctsu.ox.ac.uk/crystal/field.cgi?id=1757.

family_satisfaction – Family satisfaction – index. Participants were asked the question *In general how satisfied are you with your family relationships?* and given the options *Extremely happy*, *Very happy*, *Moderately happy*, *Moderately unhappy*, *Very unhappy*, *Extremely unhappy*, *Do not know* and P*refer not to answer* which were encoded 6, 5, 4, 3, 2, 1, NA and NA respectively. For more details see http://biobank.ctsu.ox.ac.uk/crystal/field.cgi?id=4559.

fat_pc – Body fat percentage – %. Body composition estimated by impedance measurement. Values less than 1% or greater than 75% were excluded. For more details see http://biobank.ctsu.ox.ac.uk/crystal/field.cgi?id=23099.

financial_satisfaction – Financial satisfaction – index. Participants were asked the question *In general how satisfied are you with your financial situation?* and given the options *Extremely happy*, *Very happy*, *Moderately happy*, *Moderately unhappy*, *Very unhappy*, *Extremely unhappy*, *Do not know* and *Prefer not to answer* which were encoded 6, 5, 4, 3, 2, 1, NA and NA respectively. For more details see http://biobank.ctsu.ox.ac.uk/crystal/field.cgi?id=4581.

grip_strength – Grip strength – kg. Average of left and right hand grip strength as measured by a hydraulic hand dynamometer. For more details see http://biobank.ctsu.ox.ac.uk/crystal/field.cgi?id=46.

handedness – Left-handed – index. Participants were asked the question *Are you right or left handed?* and given the options *Right-handed*, *Left-handed*, *Use both right and left hands equally* and *Prefer not to answer* which were encoded -1, 1, 0 and NA respectively. For more details see http://biobank.ctsu.ox.ac.uk/crystal/field.cgi?id=1707.

happiness – Self-reported happiness – index. Participants were asked the question *In general how happy are you?* and given the options *Extremely happy*, *Very happy*, *Moderately happy*, *Moderately unhappy*, *Very unhappy*, *Extremely unhappy*, *Do not know* and *Prefer not to answer* which were

encoded 6, 5, 4, 3, 2, 1, NA and NA respectively. For more details see http://biobank.ctsu.ox.ac.uk/crystal/field.cgi?id=20458.

headache – Headaches – TRUE/FALSE. Participants were asked the question *In the last month have you experienced any of the following that interfered with your usual activities? (You can select more than one answer)* and given ten options: *Headache*, *Facial pain*, *Neck or shoulder pain*, *Back pain*, Stom*ach or abdominal pain*, *Hip pain*, *Knee pain*, *Pain all over the body*, *None of the above* and *Prefer not to answer*. Participants who selected *Headache* were coded as 1, those who selected only *Prefer not to answer* were coded as NA and the remainder set to 0. For more details see http://biobank.ctsu.ox.ac.uk/crystal/field.cgi?id=6159.

health_satisfaction – Health satisfaction – index. Participants were asked the question *In general how satisfied are you with your health?* and given the options *Extremely happy*, *Very happy*, *Moderately happy*, *Moderately unhappy*, *Very unhappy*, *Extremely unhappy*, *Do not know* and *Prefer not to answer* which were encoded 6, 5, 4, 3, 2, 1, NA and NA respectively. For more details see http://biobank.ctsu.ox.ac.uk/crystal/field.cgi?id=4548.

hearing_acuity – Hearing acuity – no units. Mean of left and right ear Speech Reception Threshold (SRT), defined here as the signal-to-noise ratio at which half of the presented speech can be understood correctly. This value was multiplied by -1 so that larger values correspond to better hearing. For more details see http://biobank.ctsu.ox.ac.uk/crystal/field.cgi?id=20019.

heelbone_density – Heelbone density – Z-score. Mean of left and right heelbone density T-score calculated from an ultrasound heel Bone Mineral Density measurement and normalised within each sex. For more details see http://biobank.ctsu.ox.ac.uk/crystal/field.cgi?id=4106.

infertility_self_declared_glm – Self-reported infertility – TRUE/FALSE. UKB participants were asked in a verbal interview with a trained nurse to describe any serious illness or disabilities. Responses were classified in a tree-structured list used by clinic nurses to code non-cancer illnesses. The values 1403 and 1404 correspond to female and male infertility respectively and participants with these either of these responses were encoded 1. All other participants who completed the verbal interview were encoded 0. For more details see http://biobank.ctsu.ox.ac.uk/crystal/field.cgi?id=20002.

irritability_glm – Self-reported irritability – TRUE/FALSE. Participants were asked the question *Are you an irritable person?* and given the options *Yes*, *No*, *Do not know* and *Prefer not to answer* which were encoded 1, 0, NA and NA respectively. For more details see http://biobank.ctsu.ox.ac.uk/crystal/field.cgi?id=1940.

job_satisfaction – Job satisfaction – index. Participants were asked the question *In general how satisfied are you with the work that you do?* and given the options *Extremely happy*, *Very happy*, *Moderately happy*, Mod*erately unhappy*, *Very unhappy*, *Extremely unhappy*, *Do not know* and *Prefer not to answer* which were encoded 6, 5, 4, 3, 2, 1, NA and NA respectively. For more details see http://biobank.ctsu.ox.ac.uk/crystal/field.cgi?id=4537.

match_time – Reaction time – ms. Participants were shown two cards at a time on a touchscreen and instructed to press a button as quickly as possible when the symbols on the cards match. This field is the mean duration to first press of snap-button summed over rounds in which both cards matched. It gives a crude measure of the raw processing + reaction speed of a participant. For more details see http://biobank.ctsu.ox.ac.uk/crystal/field.cgi?id=20023.

memory – Memory – count. The participant was shown a 2-digit number to remember. The number then disappeared and after a short while they were asked to enter the number onto the screen. The number became one digit longer each time they remembered correctly (up to a maximum of 12 digits). This trait is the longest number correctly recalled during the numeric memory test. For more details see http://biobank.ctsu.ox.ac.uk/crystal/field.cgi?id=4282.

miscarriage – Miscarriage – TRUE/FALSE. Women only. Female participants were asked the question *Have you ever had any stillbirths, spontaneous miscarriages or terminations?* and given the options *Yes*, *No*, *Do not know* and *Prefer not to answer* which were encoded 1, 0, NA and NA respectively. For more details see http://biobank.ctsu.ox.ac.uk/crystal/field.cgi?id=2774.

moderate_activity – Frequency of moderate activity – count. Participants were asked the question *In a typical week, on how many days did you do 10 minutes or more of moderate physical activities like carrying light loads, cycling at normal pace? (Do not include walking)*. Values less than 0 or greater than 7 were rejected. For more details see http://biobank.ctsu.ox.ac.uk/crystal/field.cgi?id=884.

moody_glm – Moody – TRUE/FALSE. Participants were asked the question *Does your mood often go up and down?* and given the options *Yes*, *No*, *Do not know* and *Prefer not to answer* which were encoded 1, 0, NA and NA respectively. For more details see http://biobank.ctsu.ox.ac.uk/crystal/field.cgi?id=1920.

motorway_speeding – Driving speed – index. Participants were asked the question *How often do you drive faster than the speed limit on the motorway?* and given the options *Never/rarely*, *Sometimes*, *Often*, *Most of the time* ,*Do not drive on the motorway*, *Do not know* and *Prefer not to answer* which were encoded 0, 1, 2, 3, NA, NA and NA respectively. For more details see http://biobank.ctsu.ox.ac.uk/crystal/field.cgi?id=1100.

neuroticism – Neuroticism – index. An externally derived summary score of neuroticism, based on 12 neurotic behaviour domains reported in UKB. Values range from 0 to 12 with higher scores corresponding to increased neurotic behaviour. For more details see http://biobank.ctsu.ox.ac.uk/crystal/field.cgi?id=20127.

number_sexual_partners – Number sexual partners – count. Participants were asked the question *About how many sexual partners have you had in your lifetime*?. Subjects who answered *Do not know* or *Prefer not to answer* were set to NA, otherwise values were capped at 100. For more details see http://biobank.ctsu.ox.ac.uk/crystal/field.cgi?id=2149.

overall_health – Self-reported overall health – index. Participants were asked the question *In general how would you rate your overall health?* and given the options *Excellent*, *Good*, *Fair*, *Poor*, *Do not know* and *Prefer not to answer* which were encoded 3, 2, 1, 0, NA and NA respectively. For more details see http://biobank.ctsu.ox.ac.uk/crystal/field.cgi?id=2178.

pacemaker_glm – Pacemaker – TRUE/FALSE. Participants were asked by an interviewer if they have a pace-maker before the body impedance measures. Those that answered *Yes* were encode 1, otherwise 0. http://biobank.ctsu.ox.ac.uk/crystal/field.cgi?id=3079.

pgrs_edu – Polygenic score for Education Attained – years. A polygenic score for Education Attained (EA) calculated from 159 genome-wide significant SNPs reported in a GWAS of Education Attained [Okbay et al. 2016] and imputed in UKB using the UK10K + 1000 Genomes panel. This polygenic score explains 0.9% of the residual variance of EA in the UKB British cohort after conditioning on sex and age.

pgrs_height – Polygenic score for Height – metres. A polygenic score for height calculated from 697 genome-wide significant SNPs reported in a GWAS of height [Wood et al. 2014] and imputed in UKB using the UK10K + 1000 Genomes panel. This polygenic score explains 18.7% of the residual variance of height in the UKB British cohort after conditioning on sex and age.

potassium – Urinary Potassium – mML^{-1}. Potassium in urine measured by ISE (ion selective electrode) analysis on a Beckman Coulter AU5400. For more details see http://biobank.ctsu.ox.ac.uk/crystal/field.cgi?id=30520.

risk_glm – Self-reported risk taker – TRUE/FALSE. Participants were asked the question *Would you describe yourself as someone who takes risks?* and given the options *Yes*, *No*, *Do not know* and *Prefer not to answer* which were encoded 1, 0, NA and NA respectively. For more details see http://biobank.ctsu.ox.ac.uk/crystal/field.cgi?id=2040.

sleep_duration – Sleep duration – hours. Participants were asked the question *About how many hours sleep do you get in every 24 hours? (please include naps)*. Subjects who answered *Do not know* or *Prefer not to answer* were set to NA, as were values less than 1 or greater than 23. For more details see http://biobank.ctsu.ox.ac.uk/crystal/field.cgi?id=1160.

sodium – Urinary Sodium – mML^{-1}. Sodium in urine measured by ISE (ion selective electrode) analysis on a Beckman Coulter AU5400. For more details see http://biobank.ctsu.ox.ac.uk/crystal/field.cgi?id=30530.

vigorous_activity – Frequency of vigorous activity – count. Participants were asked the question *In a typical week, how many days did you do 10 minutes or more of vigorous physical activity? (These are activities that make you sweat or breathe hard such as fast cycling, aerobics, heavy lifting)*. Values less than 0 or greater than 7 were rejected. For more details see http://biobank.ctsu.ox.ac.uk/crystal/field.cgi?id=904.

visual_acuity – Visual acuity – negative log(MAR). Mean of left and right visual acuity as defined by the smallest size letters that can be reliably identified at a 4 metres. The UK Biobank system is based on a traditional LogMar chart. This log(MAR) value was multiplied by -1 so that larger values correspond to better vision. For more details see http://biobank.ctsu.ox.ac.uk/crystal/field.cgi?id=5201.

walking_pace – Walking pace – index. Participants were asked the question *How would you describe your usual walking pace?* and given the options *Slow pace*, *Steady average pace*, *Brisk pace*, *None of the above* and *Prefer not to answer* which were encoded 0, 1, 2, NA and NA respectively. For more details see http://biobank.ctsu.ox.ac.uk/crystal/field.cgi?id=924.

SUPPLEMENTARY NOTE 2: Personal acknowledgments

Yukinori Okada Y.O. BioBank Japan was supported by the Tailor-Made Medical Treatment program (the BioBank Japan Project) of the Ministry of Education, Culture, Sports, Science, and Technology (MEXT), the Japan Agency for Medical Research and Development (AMED).
Xiuqing Guo X.G. MESA (Multi-Ethnic Study of Atherosclerosis): MESA and the MESA SHARe project are supported by the National Heart, Lung, and Blood Institute (NHLBI) in collaboration with MESA investigators. Support for MESA is provided by contracts HHSN268201500003I, N01-HC-95159, N01-HC-95160, N01-HC-95161, N01-HC-95162, N01-HC-95163, N01-HC-95164, N01-HC-95165, N01-HC-95166, N01-HC-95167, N01-HC-95168, N01-HC-95169, UL1-TR-000040, UL1-TR-001079, UL1-TR-001420, UL1-TR-001881, and DK063491. Funding for SHARe genotyping was

provided by NHLBI Contract N02-HL-64278. The provision of genotyping data was supported in part by the National Center for Advancing Translational Sciences, CTSI grant UL1TR001881, and the National Institute of Diabetes and Digestive and Kidney Disease Diabetes Research Center (DRC) grant DK063491 to the Southern California Diabetes Endocrinology Research Center.
Carolina Medina-Gomez C.M.-G. Supported by the Netherlands Organization for Health Research and Development (ZonMw VIDI 016.136.367).
Annelot M Dekker A.M.D. ALS Foundation the Netherlands
He Gao H.G. The Airwave Health Monitoring Study is funded by the Home Office (grant number 780-TETRA) with additional support from the National Institute for Health Research (NIHR) Imperial Biomedical Research Centre (BRC). The study uses the computing resources of the UK MEDical BIOinformatics partnership - aggregation, integration, visualization and analysis of large, complex data (UK MED-BIO), which is supported by the Medical Research Council (MR/L01632X/1).
May E Montasser M.E.M. The Amish studies are supported by grants and contracts from the NIH, including R01 AG18728, R01 HL088119, U01 GM074518, U01 HL072515-06, U01 HL84756, R01 DK54261, U01 HL137181, , the University of Maryland General Clinical Research Center, grant M01 RR 16500, the Mid-Atlantic Nutrition Obesity Research Center grant P30 DK72488, and by the Baltimore Diabetes Research and Training Center grant P60DK79637, the American Heart Association: 12SDG9280031 and 17GRNT33661168. In addition, this project was supported by National Research Initiative Competitive Grant no. 2007-35205-17883 from the USDA National Institute of Food and Agriculture. We gratefully thank our Amish community and research volunteers for their long-standing partnership in research, and acknowledge the dedication of our Amish liaisons, field workers and the Amish Research Clinic staff, without which these studies would not have been possible.
Daniel Shriner D.S. The authors acknowledge with thanks the participants in the AADM project, their families and their physicians. The study was supported in part by the Intramural Research Program of the National Institutes of Health in the Center for Research on Genomics and Global Health (CRGGH). The CRGGH is supported by the National Human Genome Research Institute (NHGRI), the National Institute of Diabetes and Digestive and Kidney Diseases (NIDDK), the Center for Information Technology, and the Office of the Director at the National Institutes of Health (1ZIAHG200362). Support for participant recruitment and initial genetic studies of the AADM study was provided by NIH grant No. 3T37TW00041-03S2 from the Office of Research on Minority Health.
Cassandra N Spracklen C.N.S. American Heart Association 17POST3650016
Rona J Strawbridge R.J.S. RJS is supported by a UKRI Innovation- HDR-UK Fellowship (MR/S003061/1)
Niek Verweij N.V. NWO-VENI (016.186.125)
Helen R Warren H.R.W. This work was funded by the Medical Research Council of Great Britain (grant number: G9521010D). The BRIGHT study is extremely grateful to all the patients who participated in the study and the BRIGHT nursing team. We wish to acknowledge the support of the NIHR Cardiovascular Biomedical Research Unit at Barts and Queen Mary University of London, UK
Robyn E Wootton R.E.W. Funded by the MRC [MC_UU_12013/6] and the NIHR Biomedical Research Centre at the University Hospitals Bristol NHS Foundation Trust and the University of Bristol.
Lisa R Yanek L.R.Y. GeneSTAR was supported by grants from the National Institutes of Health/National Heart, Lung, and Blood Institute (U01 HL72518,HL087698, HL49762, HL59684, HL58625, HL071025, HL092165, HL099747, K23HL105897),
Lisa R Yanek L.R.Y. National Institutes of Health/National Institute of Nursing Research (NR0224103, NR008153),
Lisa R Yanek L.R.Y. National Institutes of Health/National Institute of Neurological Disorders and Stroke (NS062059)
Lisa R Yanek L.R.Y. National Institutes of Health/National Center for Research Resources (M01-RR000052) to the Johns Hopkins General Clinical Research Center,

Lisa R Yanek L.R.Y. and National Institutes of Health/National Center for Research Resources/ National Center for Advancing Translational Sciences (UL1 RR 025005) to the Johns Hopkins Institute for Clinical & Translational Research

Jie Yao J.Yao MESA (Multi-Ethnic Study of Atherosclerosis): MESA and the MESA SHARe project are supported by the National Heart, Lung, and Blood Institute (NHLBI) in collaboration with MESA investigators. Support for MESA is provided by contracts HHSN268201500003I, N01-HC-95159, N01-HC-95160, N01-HC-95161, N01-HC-95162, N01-HC-95163, N01-HC-95164, N01-HC-95165, N01-HC-95166, N01-HC-95167, N01-HC-95168, N01-HC-95169, UL1-TR-000040, UL1-TR-001079, UL1-TR-001420, UL1-TR-001881, and DK063491. Funding for SHARe genotyping was provided by NHLBI Contract N02-HL-64278. The provision of genotyping data was supported in part by the National Center for Advancing Translational Sciences, CTSI grant UL1TR001881, and the National Institute of Diabetes and Digestive and Kidney Disease Diabetes Research Center (DRC) grant DK063491 to the Southern California Diabetes Endocrinology Research Center.

Adebowale A Adeyemo A.A.A. The authors acknowledge with thanks the participants in the AADM project, their families and their physicians. The study was supported in part by the Intramural Research Program of the National Institutes of Health in the Center for Research on Genomics and Global Health (CRGGH). The CRGGH is supported by the National Human Genome Research Institute (NHGRI), the National Institute of Diabetes and Digestive and Kidney Diseases (NIDDK), the Center for Information Technology, and the Office of the Director at the National Institutes of Health (1ZIAHG200362). Support for participant recruitment and initial genetic studies of the AADM study was provided by NIH grant No. 3T37TW00041-03S2 from the Office of Research on Minority Health.

Matthew L Albert M.L.A. The Milieu Intérieur cohort was supported by the French government's program Investissement d'Avenir, managed by the Agence Nationale de la Recherche (reference 10-LABX-69-01).

Matthew A Allison M.A.A. MESA (Multi-Ethnic Study of Atherosclerosis): MESA and the MESA SHARe project are supported by the National Heart, Lung, and Blood Institute (NHLBI) in collaboration with MESA investigators. Support for MESA is provided by contracts HHSN268201500003I, N01-HC-95159, N01-HC-95160, N01-HC-95161, N01-HC-95162, N01-HC-95163, N01-HC-95164, N01-HC-95165, N01-HC-95166, N01-HC-95167, N01-HC-95168, N01-HC-95169, UL1-TR-000040, UL1-TR-001079, UL1-TR-001420, UL1-TR-001881, and DK063491. Funding for SHARe genotyping was provided by NHLBI Contract N02-HL-64278. The provision of genotyping data was supported in part by the National Center for Advancing Translational Sciences, CTSI grant UL1TR001881, and the National Institute of Diabetes and Digestive and Kidney Disease Diabetes Research Center (DRC) grant DK063491 to the Southern California Diabetes Endocrinology Research Center.

Maris Alver M.AlverEGCUT was supported by Estonian Research Council [IUT20-60, IUT24-6, PUT1660 to T.E and PUT1665 to K.F.; European Union Horizon 2020 [692145]; European Union through the European Regional Development Fund [2014-2020.4.01.15-0012 GENTRANSMED];

Amy R Bentley A.R.B. The AADM and HUFS studies were supported in part by the Intramural Research Program of the National Institutes of Health in the Center for Research on Genomics and Global Health (CRGGH). The CRGGH is supported by the National Human Genome Research Institute (NHGRI), the National Institute of Diabetes and Digestive and Kidney Diseases (NIDDK), the Center for Information Technology, and the Office of the Director at the National Institutes of Health (1ZIAHG200362).

Guanjie Chen G.Chen The AADM and HUFS studies were supported in part by the Intramural Research Program of the National Institutes of Health in the Center for Research on Genomics and Global Health (CRGGH). The CRGGH is supported by the National Human Genome Research Institute (NHGRI), the National Institute of Diabetes and Digestive and Kidney Diseases (NIDDK), the Center for Information Technology, and the Office of the Director at the National Institutes of Health (1ZIAHG200362).

Yii-Der I Chen Y.-D.I.C. TUDR (Taiwan-US Diabetic Retinopathy): This study was supported by the National Eye Institute of the National Institutes of Health (EY014684 to J.I.R. and Y.-D.I.C.) and ARRA Supplement (EY014684-03S1, -04S1), the Eye Birth Defects Foundation Inc., the National Science Council, Taiwan (NSC 98-2314-B-075A-002-MY3 to W.H.S.) and the Taichung Veterans General Hospital, Taichung, Taiwan (TCVGH-1003001C to W.H.S .). DNA handling and genotyping were supported in part by the National Center for Advancing Translational Sciences, CTSI grant UL1TR001880 and the National Institute of Diabetes and Digestive and Kidney Disease Diabetes Research Center (DRC) grant DK063491 to the Southern California Diabetes Endocrinology Research Center.
Yen-Feng Chiu Y.-F.C. The TAICHI study was supported by grants from the National Health Research Institutes, Taiwan (PH-099-PP-03, PH-100-PP-03, and PH-101-PP-03); the National Science Council, Taiwan (NSC 101-2314-B-075A-006-MY3, MOST 104-2314-B-075A-006-MY3, MOST 104-2314-B-075A-007, and MOST 105-2314-B-075A-003); and the Taichung Veterans General Hospital, Taiwan (TCVGH-1020101C, TCVGH-020102D, TCVGH-1023102B, TCVGH-1023107D, TCVGH-1030101C, TCVGH-1030105D, TCVGH-1033503C, TCVGH-1033102B, TCVGH-1033108D, TCVGH-1040101C, TCVGH-1040102D, TCVGH-1043504C, and TCVGH-1043104B); it was also supported in part by the National Center for Advancing Translational Sciences (CTSI grant UL1TR001881).
Ayo P Doumatey A.P.D. The authors acknowledge with thanks the participants in the AADM project, their families and their physicians. The study was supported in part by the Intramural Research Program of the National Institutes of Health in the Center for Research on Genomics and Global Health (CRGGH). The CRGGH is supported by the National Human Genome Research Institute (NHGRI), the National Institute of Diabetes and Digestive and Kidney Diseases (NIDDK), the Center for Information Technology, and the Office of the Director at the National Institutes of Health (1ZIAHG200362). Support for participant recruitment and initial genetic studies of the AADM study was provided by NIH grant No. 3T37TW00041-03S2 from the Office of Research on Minority Health.
Nduna Dzimiri N.D. The authors would like to thank Editha Andres, Nejat Mazher and Dr. Maie Alshaid for their assistance in patient sample and clinical data collection.
Paul Elliott P.E. P.E. acknowledges support from the National Institute for Health Research (NIHR) Imperial Biomedical Research Centre, the NIHR Health Protection Research Unit in Health Impact of Environmental Hazards (HPRU-2012-10141), and the Medical Research Council (MRC) and Public Health England (PHE) Centre for Environment and Health (MR/L01341X/1). This work used the computing resources of the UK MEDical BIOinformatics partnership UK MED-BIO)supported by the Medical Research Council (MR/L01632X/1). P.E. is a UK Dementia Research Institute (DRI) professor, UK DRI at Imperial College London, funded by the MRC, Alzheimer's Society and Alzheimer's Research UK; and associate director, Health Data Research UK - London funded by a consortium led by the MRC.
Janine F Felix J.F.F. Received funding from the European Union's Horizon 2020 research and innovation programme under grant agreements No 633595 (DynaHEALTH) and No 733206 (LifeCycle).
Krista Fischer K.F. EGCUT was supported by Estonian Research Council [IUT20-60, IUT24-6, PUT1660 to T.E and PUT1665 to K.F.; European Union Horizon 2020 [692145]; European Union through the European Regional Development Fund [2014-2020.4.01.15-0012 GENTRANSMED];
Anuj Goel A.G. Anuj Goel acknowledges support from BHF, Wellcome Trust, European Community Sixth Framework Program (LSHM-CT- 2007-037273), European Union Seventh Framework Programme FP7/2007-2013 under grant agreement no. HEALTH-F2-2013-601456 (CVGenes@Target), TriPartite Immunometabolism Consortium [TrIC]- Novo Nordisk Foundation's Grant number NNF15CC0018486
Mark O Goodarzi M.O.G. MESA (Multi-Ethnic Study of Atherosclerosis): MESA and the MESA SHARe project are supported by the National Heart, Lung, and Blood Institute (NHLBI) in collaboration with MESA investigators. Support for MESA is provided by contracts HHSN268201500003I, N01-HC-95159, N01-HC-95160, N01-HC-95161, N01-HC-95162, N01-HC-

95163, N01-HC-95164, N01-HC-95165, N01-HC-95166, N01-HC-95167, N01-HC-95168, N01-HC-95169, UL1-TR-000040, UL1-TR-001079, UL1-TR-001420, UL1-TR-001881, and DK063491. Funding for SHARe genotyping was provided by NHLBI Contract N02-HL-64278. The provision of genotyping data was supported in part by the National Center for Advancing Translational Sciences, CTSI grant UL1TR001881, and the National Institute of Diabetes and Digestive and Kidney Disease Diabetes Research Center (DRC) grant DK063491 to the Southern California Diabetes Endocrinology Research Center.
Chao Agnes Hsiung C.A.Hsiung The THRV study is supported by National Heart, Lung, and Blood Institute grant R01HL111249.
Jinyan Huang J.H. the National Natural Science Foundation of China (Grants 81570122, 81770205);
Yi-Jen Hung Y.-J.H. The TAICHI study was supported by grants from the National Health Research Institutes, Taiwan (PH-099-PP-03, PH-100-PP-03, and PH-101-PP-03); the National Science Council, Taiwan (NSC 101-2314-B-075A-006-MY3, MOST 104-2314-B-075A-006-MY3, MOST 104-2314-B-075A-007, and MOST 105-2314-B-075A-003); and the Taichung Veterans General Hospital, Taiwan (TCVGH-1020101C, TCVGH-020102D, TCVGH-1023102B, TCVGH-1023107D, TCVGH-1030101C, TCVGH-1030105D, TCVGH-1033503C, TCVGH-1033102B, TCVGH-1033108D, TCVGH-1040101C, TCVGH-1040102D, TCVGH-1043504C, and TCVGH-1043104B); it was also supported in part by the National Center for Advancing Translational Sciences (CTSI grant UL1TR001881).
Zoltán Kutalik Z.K. Swiss National Science Foundation (31003A_169929)
Zoltán Kutalik Z.K. SystemsX.ch (51RTP0_151019)
Deborah A Lawlor D.A.L. Born in Bradford (BiB) receives core infrastructure funding from the Wellcome Trust (WT101597MA) a joint grant from the UK Medical Research Council (MRC) and UK Economic and Social Science Research Council (ESRC) (MR/N024397/1) and the National Institute for Health Research (NIHR) under its Collaboration for Applied Health Research and Care (CLAHRC) for Yorkshire and Humber. The research presented in this paper is supported by the British Heart Foundation (CS/16/4/32482), US National Institute of Health (R01 DK10324), the European Research Council under the European Union's Seventh Framework Programme (FP7/2007-2013) / ERC grant agreement no 669545), and the NIHR Biomedical Centre at the University Hospitals Bristol NHS Foundation Trust and the University of Bristol. D.A.L. works in a unit that receives UK MRC funding (MC_UU_12013/5) and D.A.L. is an NIHR senior investigator (NF-SI-0611-10196). The funders had no role in the design of the study, the collection, analysis, or interpretation of the data; the writing of the manuscript, or the decision to submit the manuscript for publication
Wen-Jane Lee W.-J.L. The THRV study is supported by National Heart, Lung, and Blood Institute grant R01HL111249.
Stephanie J London S.J.L. Supported by the Intramural Research Program of the NIH, National Institute of Environmental Health Sciences, NIH ZO1 ES043012
Reedik Mägi R.M. EGCUT was supported by Estonian Research Council [IUT20-60, IUT24-6, PUT1660 to T.E and PUT1665 to K.F.; European Union Horizon 2020 [692145]; European Union through the European Regional Development Fund [2014-2020.4.01.15-0012 GENTRANSMED];
Ani W Manichaikul A.W.M. MESA (Multi-Ethnic Study of Atherosclerosis): MESA and the MESA SHARe project are supported by the National Heart, Lung, and Blood Institute (NHLBI) in collaboration with MESA investigators. Support for MESA is provided by contracts HHSN268201500003I, N01-HC-95159, N01-HC-95160, N01-HC-95161, N01-HC-95162, N01-HC-95163, N01-HC-95164, N01-HC-95165, N01-HC-95166, N01-HC-95167, N01-HC-95168, N01-HC-95169, UL1-TR-000040, UL1-TR-001079, UL1-TR-001420, UL1-TR-001881, and DK063491. Funding for SHARe genotyping was provided by NHLBI Contract N02-HL-64278. The provision of genotyping data was supported in part by the National Center for Advancing Translational Sciences, CTSI grant UL1TR001881, and the National Institute of Diabetes and Digestive and Kidney Disease Diabetes Research Center (DRC) grant DK063491 to the Southern California Diabetes Endocrinology Research Center.

Lili Milani L.M. EGCUT was supported by Estonian Research Council [IUT20-60, IUT24-6, PUT1660 to T.E and PUT1665 to K.F.; European Union Horizon 2020 [692145]; European Union through the European Regional Development Fund [2014-2020.4.01.15-0012 GENTRANSMED];
Alison D Murray A.D.M. Generation Scotland received core support from the Chief Scientist Office of the Scottish Government Health Directorates [CZD/16/6] and the Scottish Funding Council [HR03006]. Genotyping of the GS:SFHS samples was carried out by the Genetics Core Laboratory at the Wellcome Trust Clinical Research Facility, Edinburgh, Scotland and was funded by the Medical Research Council UK and the Wellcome Trust (Wellcome Trust Strategic Award STratifying Resilience and Depression Longitudinally (STRADL) Reference 104036/Z/14/Z).
Josyf C Mychaleckyj J.C.M. MESA (Multi-Ethnic Study of Atherosclerosis): MESA and the MESA SHARe project are supported by the National Heart, Lung, and Blood Institute (NHLBI) in collaboration with MESA investigators. Support for MESA is provided by contracts HHSN268201500003I, N01-HC-95159, N01-HC-95160, N01-HC-95161, N01-HC-95162, N01-HC-95163, N01-HC-95164, N01-HC-95165, N01-HC-95166, N01-HC-95167, N01-HC-95168, N01-HC-95169, UL1-TR-000040, UL1-TR-001079, UL1-TR-001420, UL1-TR-001881, and DK063491. Funding for SHARe genotyping was provided by NHLBI Contract N02-HL-64278. The provision of genotyping data was supported in part by the National Center for Advancing Translational Sciences, CTSI grant UL1TR001881, and the National Institute of Diabetes and Digestive and Kidney Disease Diabetes Research Center (DRC) grant DK063491 to the Southern California Diabetes Endocrinology Research Center.
Sandosh Padmanabhan S.P. Generation Scotland received core support from the Chief Scientist Office of the Scottish Government Health Directorates [CZD/16/6] and the Scottish Funding Council [HR03006]. Genotyping of the GS:SFHS samples was carried out by the Genetics Core Laboratory at the Wellcome Trust Clinical Research Facility, Edinburgh, Scotland and was funded by the Medical Research Council UK and the Wellcome Trust (Wellcome Trust Strategic Award STratifying Resilience and Depression Longitudinally (STRADL) Reference 104036/Z/14/Z).
Ozren Polasek O.P. CROATIA cohorts were funded by the Medical Research Council UK, The Croatian Ministry of Science, Education and Sports (grant 216-1080315-0302), the European Union framework program 6 EUROSPAN project (contract no. LSHG-CT-2006-018947), the Croatian Science Foundation (grant 8875) and the Centre of Excellence in Personalized Health Care.
Neil Poulter N.Poulter This work was funded by the National Institutes for Health Research (NIHR) as part of the portfolio of translational research of the NIHR Barts Biomedical Research Centre and the NIHR Biomedical Research Centre at Imperial College, the International Centre for Circulatory Health Charity and the Medical Research Council through G952010. We thank all ASCOT trial participants, physicians, nurses, and practices in the participating countries for their important contribution to the study.
Lluis Quintana-Murci L.Q.-M. The Milieu Intérieur cohort was supported by the French government's program Investissement d'Avenir, managed by the Agence Nationale de la Recherche (reference 10-LABX-69-01).
Dabeeru C Rao D.C.R. The THRV study is supported by National Heart, Lung, and Blood Institute grant R01HL111249.
Stephen S Rich S.S.R. MESA (Multi-Ethnic Study of Atherosclerosis): MESA and the MESA SHARe project are supported by the National Heart, Lung, and Blood Institute (NHLBI) in collaboration with MESA investigators. Support for MESA is provided by contracts HHSN268201500003I, N01-HC-95159, N01-HC-95160, N01-HC-95161, N01-HC-95162, N01-HC-95163, N01-HC-95164, N01-HC-95165, N01-HC-95166, N01-HC-95167, N01-HC-95168, N01-HC-95169, UL1-TR-000040, UL1-TR-001079, UL1-TR-001420, UL1-TR-001881, and DK063491. Funding for SHARe genotyping was provided by NHLBI Contract N02-HL-64278. The provision of genotyping data was supported in part by the National Center for Advancing Translational Sciences, CTSI grant UL1TR001881, and the National Institute of Diabetes and Digestive and Kidney Disease Diabetes Research Center (DRC) grant DK063491 to the Southern California Diabetes Endocrinology Research Center.

Cornelius A Rietveld C.A.R. Netherlands Organisation for Scientific Research Veni grant 016.165.004
Maria Sabater-Lleal M.S.-L. MS-L is supported by Miguel Servet contract (ISCIII CP17/00142) from the Spanish Ministry of Health, and acknowledges funding from the Swedish Heart and Lung Foundation (#20160290).
Veikko Salomaa V.S. VS was supported By the Finnish Foundation for Cardiovascular Research.
Kevin Sandow K.Sandow The TAICHI study was supported by grants from the National Health Research Institutes, Taiwan (PH-099-PP-03, PH-100-PP-03, and PH-101-PP-03); the National Science Council, Taiwan (NSC 101-2314-B-075A-006-MY3, MOST 104-2314-B-075A-006-MY3, MOST 104-2314-B-075A-007, and MOST 105-2314-B-075A-003); and the Taichung Veterans General Hospital, Taiwan (TCVGH-1020101C, TCVGH-020102D, TCVGH-1023102B, TCVGH-1023107D, TCVGH-1030101C, TCVGH-1030105D, TCVGH-1033503C, TCVGH-1033102B, TCVGH-1033108D, TCVGH-1040101C, TCVGH-1040102D, TCVGH-1043504C, and TCVGH-1043104B); it was also supported in part by the National Center for Advancing Translational Sciences (CTSI grant UL1TR001881).
Bengt Sennblad B.S. Bengt Sennblad is financially supported by the Knut and Alice Wallenberg Foundation as part of the National Bioinformatics Infrastructure Sweden at SciLifeLab.
Peter J Sever P.J.S. This work was funded by the National Institutes for Health Research (NIHR) as part of the portfolio of translational research of the NIHR Barts Biomedical Research Centre and the NIHR Biomedical Research Centre at Imperial College, the International Centre for Circulatory Health Charity and the Medical Research Council through G952010. We thank all ASCOT trial participants, physicians, nurses, and practices in the participating countries for their important contribution to the study.
Wayne H-H Sheu W.H.-H.S. TUDR (Taiwan-US Diabetic Retinopathy): This study was supported by the National Eye Institute of the National Institutes of Health (EY014684 to J.I.R. and Y.-D.I.C.) and ARRA Supplement (EY014684-03S1, -04S1), the Eye Birth Defects Foundation Inc., the National Science Council, Taiwan (NSC 98-2314-B-075A-002-MY3 to W.H.S.) and the Taichung Veterans General Hospital, Taichung, Taiwan (TCVGH-1003001C to W.H.S .). DNA handling and genotyping were supported in part by the National Center for Advancing Translational Sciences, CTSI grant UL1TR001880 and the National Institute of Diabetes and Digestive and Kidney Disease Diabetes Research Center (DRC) grant DK063491 to the Southern California Diabetes Endocrinology Research Center.
Timo Tonis Sikka T.T.S. EGCUT was supported by Estonian Research Council [IUT20-60, IUT24-6, PUT1660 to T.E and PUT1665 to K.F.; European Union Horizon 2020 [692145]; European Union through the European Regional Development Fund [2014-2020.4.01.15-0012 GENTRANSMED];
Blair H Smith B.H.S. Generation Scotland received core support from the Chief Scientist Office of the Scottish Government Health Directorates [CZD/16/6] and the Scottish Funding Council [HR03006]. Genotyping of the GS:SFHS samples was carried out by the Genetics Core Laboratory at the Wellcome Trust Clinical Research Facility, Edinburgh, Scotland and was funded by the Medical Research Council UK and the Wellcome Trust (Wellcome Trust Strategic Award STratifying Resilience and Depression Longitudinally (STRADL) Reference 104036/Z/14/Z).
Alice Stanton A.Stanton This work was funded by the National Institutes for Health Research (NIHR) as part of the portfolio of translational research of the NIHR Barts Biomedical Research Unit and the NIHR Biomedical Research Centre at Imperial College, the International Centre for Circulatory Health Charity and the Medical Research Council through G952010. We thank all ASCOT trial participants, physicians, nurses, and practices in the participating countries for their important contribution to the study.
Kent D Taylor K.D.T. MESA (Multi-Ethnic Study of Atherosclerosis): MESA and the MESA SHARe project are supported by the National Heart, Lung, and Blood Institute (NHLBI) in collaboration with MESA investigators. Support for MESA is provided by contracts HHSN268201500003I, N01-HC-95159, N01-HC-95160, N01-HC-95161, N01-HC-95162, N01-HC-95163, N01-HC-95164, N01-HC-

95165, N01-HC-95166, N01-HC-95167, N01-HC-95168, N01-HC-95169, UL1-TR-000040, UL1-TR-001079, UL1-TR-001420, UL1-TR-001881, and DK063491. Funding for SHARe genotyping was provided by NHLBI Contract N02-HL-64278. The provision of genotyping data was supported in part by the National Center for Advancing Translational Sciences, CTSI grant UL1TR001881, and the National Institute of Diabetes and Digestive and Kidney Disease Diabetes Research Center (DRC) grant DK063491 to the Southern California Diabetes Endocrinology Research Center.
Russell P Tracy R.P.T. MESA (Multi-Ethnic Study of Atherosclerosis): MESA and the MESA SHARe project are supported by the National Heart, Lung, and Blood Institute (NHLBI) in collaboration with MESA investigators. Support for MESA is provided by contracts HHSN268201500003I, N01-HC-95159, N01-HC-95160, N01-HC-95161, N01-HC-95162, N01-HC-95163, N01-HC-95164, N01-HC-95165, N01-HC-95166, N01-HC-95167, N01-HC-95168, N01-HC-95169, UL1-TR-000040, UL1-TR-001079, UL1-TR-001420, UL1-TR-001881, and DK063491. Funding for SHARe genotyping was provided by NHLBI Contract N02-HL-64278. The provision of genotyping data was supported in part by the National Center for Advancing Translational Sciences, CTSI grant UL1TR001881, and the National Institute of Diabetes and Digestive and Kidney Disease Diabetes Research Center (DRC) grant DK063491 to the Southern California Diabetes Endocrinology Research Center.
Jan H Veldink J.H.V. ALS foundation the Netherlands
Ya Xing Wang Y.X.W. no
Nicholas J Wareham N.J.W. MRC Epidemiology Unit, Fenland study, EPIC-Norfolk case-cohort study funding: this study was funded by the United Kingdom's Medical Research Council through grants MC_UU_12015/1, MC_PC_13046, MC_PC_13048 and MR/L00002/1
Chittaranjan S Yajnik C.S.Y. The Wellcome Trust, London, UK; MRC, UK, Department of Biotechnology, Government of India, New Delhi, India
Jie Zhou J.Z. he AADM and HUFS studies were supported in part by the Intramural Research Program of the National Institutes of Health in the Center for Research on Genomics and Global Health (CRGGH). The CRGGH is supported by the National Human Genome Research Institute (NHGRI), the National Institute of Diabetes and Digestive and Kidney Diseases (NIDDK), the Center for Information Technology, and the Office of the Director at the National Institutes of Health (1ZIAHG200362).
Folkert W Asselbergs F.W.A. Folkert W. Asselbergs is supported by UCL Hospitals NIHR Biomedical Research Centre.
Stephan J L Bakker S.J.L.B. The establishment of the PREVEND study was primarily funded by the Dutch Kidney Foundation.
Leonard H van den Berg L.H.v.d.B. Supported by The Netherlands ALS Foundation
Sonja I Berndt S.I.B. Pegasus was supported by the Intramural Research Program of the Division of Cancer Epidemiology and Genetics, National Cancer Institute, NIH.
Dwaipayan Bharadwaj D.B. Funding source:Council of Scientific and Industrial Research [CSIR], Government of India through Centre for Cardiovascular and Metabolic Disease Research [CARDIOMED] project [Grant No: BSC0122).
Archie Campbell A.C. Generation Scotland received core support from the Chief Scientist Office of the Scottish Government Health Directorates [CZD/16/6] and the Scottish Funding Council [HR03006]. Genotyping of the GS:SFHS samples was carried out by the Genetics Core Laboratory at the Wellcome Trust Clinical Research Facility, Edinburgh, Scotland and was funded by the Medical Research Council UK and the Wellcome Trust (Wellcome Trust Strategic Award STratifying Resilience and Depression Longitudinally (STRADL) Reference 104036/Z/14/Z).
Mark J Caulfield M.J.C. This work was funded by the Medical Research Council of Great Britain (grant number: G9521010D). The BRIGHT study is extremely grateful to all the patients who participated in the study and the BRIGHT nursing team. This work forms part of the research themes contributing to the translational research portfolio for the NIHR Barts Cardiovascular Biomedical Research Centre.

Giriraj Ratan Chandak G.R.C. This research was supported by Council of Scientific and Industrial Research (CSIR), Ministry of Science and Technology, Government of India, India, under their XII-Five Year Plan titled CARDIOMED. The cohorts were supported by funds from Department of Biotechnology, Ministry of Science and Technology, Government of India, India and Wellcome Trust, London, UK
Marina Ciullo M.Ciullo This work was supported by the Italian Ministry of Universities and CNR (Interomics Flagship Project, PON03PE_00060_7), the Assessorato Ricerca Regione Campania, the Fondazione con il SUD (2011-PDR-13), and the Istituto Banco di Napoli - Fondazione to MC.
Daniele Cusi D.Cusi HYPERGENES grant from EU: FP7-HEALTH-F4-2007-201550
Daniele Cusi D.Cusi InterOmics flagship grant from CNR (Italian National Institute of Research): PB05_SP3
Daniele Cusi D.Cusi ATHENA grant from EU: FP7, EU Grant Agreement 245121
George Davey-Smith G.D.-S. Funded by the MRC: http://www.mrc.ac.uk [MC_UU_12013/1]
Bjarke Feenstra B.F. Oak Foundation fellowship and Novo Nordisk Foundation grant (12955)
Mary Feitosa M.F. The FamHS was supported by RO1HL117078 from the National Heart, Lung, and Blood Institute, and R01DK08925607 from the National Institute of Diabetes and Digestive and Kidney Diseases.
Andre Franke A.F. FoCus received infrastructure support from the DFG Cluster of Excellence Inflammation at Interfaces.
Paolo Gasparini P.Gasparini Acknowledgements: All the populations recruited that took part in the study. Funds: RC-5x1000 Ministry of Health (to PG).
Christian GiegerC.Gieger The KORA study was initiated and financed by the Helmholtz Zentrum München – German Research Center for Environmental Health, which is funded by the German Federal Ministry of Education and Research (BMBF) and by the State of Bavaria. Furthermore, KORA research was supported within the Munich Center of Health Sciences (MC-Health), Ludwig-Maximilians-Universität, as part of LMUinnovativ. This study was supported by the German Center for Diabetes Research (DZD e.V.). For this publication, biosamples from the KORA Biobank as part of the Joint Biobank Munich have been used.
Struan F A Grant S.F.A.G. CHOP: We thank the network of primary care clinicians, their patients and families for their contribution to this project and clinical research facilitated through the Pediatric Research Consortium (PeRC) at The Children's Hospital of Philadelphia. Rosetta Chiavacci, Elvira Dabaghyan, Hope Thomas, Kisha Harden, Andrew Hill, Kenya Fain, Crystal Johnson-Honesty, Cynthia Drummond, Shanell Harrison and Sarah Wildrick, Cecilia Kim, Edward Frackelton, George Otieno, Kelly Thomas, Cuiping Hou, Kelly Thomas and Maria L. Garris provided expert assistance with genotyping or data collection and management. We would also like to thank Smari Kristinsson, Larus Arni Hermannsson and Asbjörn Krisbjörnssonof Raförninn ehf for their extensive software design and contribution. This research was financially supported by the Daniel B. Burke Endowed Chair for Diabetes Research, an Institute Development Award from the Children's Hospital of Philadelphia, a Research Development Award from the Cotswold Foundation and NIH grant R01-HD056465.
Struan F A Grant S.F.A.G. BMDCS: The study was funded by R01 HD58886; the Eunice Kennedy Shriver National Institute of Child Health and Human Development (NICHD) contracts (N01-HD-1-3228, -3329, -3330, -3331,-3332, -3333); and the CTSA program Grant 8 UL1 TR000077. The funders had no role in the design and conduct of the study; collection, management,analysis, and interpretation of the data; preparation, review, or approval of the manuscript; and decision to submit the manuscript for publication. We appreciate the dedication of the study participants and their families, and the support of Dr Karen Winer, Scientific Director of the Bone Mineral Density in Childhood Study.
Lyn R Griffiths L.R.G. The Norfolk Island Health Study was supported by funding from National Health and Medical Research Council of Australia (NHMRC) Project Grants. Our appreciation goes to the Norfolk Islanders who volunteered for this study.

Leif Groop L.G. The Botnia studies (L.G) have been financially supported by grants from the Sigrid Juselius Foundation, The Academy of Finland (grants no. 263401, 267882, 312063 to LG), Nordic Center of Excellence in Disease Genetics. The research leading to these results has also received funding from the European Research Council under the European Union's Seventh Framework Programme (FP7/2007-2013) / ERC grant agreement n° 269045.
Leif Groop L.G. The study has also been supported by the Ministry of Education in Finland, Municipal Heath Care Center and Hospital in Jakobstad and Health Care Centers in Vasa, Närpes and Korsholm. The skillful assistance of the Botnia Study Group is gratefully acknowledged.
Vilmundur Gudnason V.G. The AGES Reykjavik study has been funded by NIH contracts N01-AG-1-2100 and 271201200022C, the NIA Intramural Research Program, Hjartavernd (the Icelandic Heart Association), and the Althingi (the Icelandic Parliament)
Ulf Gyllensten U.G. Swedish Research Council, European Union Framework Grant,
Anders Hamsten A.Hamsten PROCARDIS was funded by the European Commission Framework 6 (FP6) program (LSHM-CT-2007-037273), the British Heart Foundation, AstaZeneca, the Swedish Research Council (8691), the Knut and Alice Wallenberg Foundation, the Swedish Heart-Lung Foundation and the Torsten and Ragnar Söderberg Foundation.
Andrew A Hicks A.A.H. In South Tyrol, the study was supported by the Ministry of Health and Department of Educational Assistance, University and Research of the Autonomous Province of Bolzano.
Hagit Hochner H.Hochner This work was supported by the National Institutes of Health [grants R01HL088884, K01HL103174, 2T32HD052462-06, T32HL007902]; the Israeli Science Foundation [grant numbers 1252/07, 552/12]; the Israel Ministry of Health [IMH49267]; and the NUS-HUJ CREATE Programme of the National Research Foundation, Singapore [Project number 370062002].
Steven C Hunt S.C.H. The hypertension genetic epidemiology network was funded by cooperative agreements (U10) with NHLBI: HL54471, HL54472, HL54473, HL54495, HL54496, HL54497, HL54509, HL54515, and 2 R01 HL55673-12. Funding also came from the Biomedical Research Program, Qatar Foundation, Qatar.
Vincent W V Jaddoe V.W.V.J. Supported by the Netherlands Organization for Health Research and Development (ZonMw VIDI 016.136.361), a Consolidator Grant from the European Research Council (ERC-2014-CoG-648916) and the European Union’s Horizon 2020 research and innovation programme under grant agreements No 633595 (DynaHEALTH) and No 733206 (LifeCycle).
Magnus Johannesson M.J. The Jan Wallander and Tom Hedelius Foundation (P2015-0001:1), The Swedish Research Council (421-2013-1061)
Jost B Jonas J.B.J. Supported by National Natural Science Foundation of China (grant # 81570835)
Meena Kumari M.Kumari The UK Household Longitudinal Study is led by the Institute for Social and Economic Research at the University of Essex. The UK Household Longitudinal Study is funded by the Economic and Social Research Council (ES/H029745/1). The survey was conducted by NatCen and the genome-wide scan data were analysed and deposited by the Wellcome Trust Sanger Institute (WT098051). Information on how to access the data can be found on the Understanding Society website https://www.understandingsociety.ac.uk/.
Markku Laakso M.Laakso The study was supported by grants from the Academy of Finland and the Sigrid Juselius Foundation.
Jari Lahti J.Lahti Academy of Finland and University of Helsinki
Wolfgang Lieb W.L. The popgen 2.0 network has been financed by the German Ministry for Education and Research; 01EY1103
Giuseppe Matullo G.Matullo The work was supported by the Compagnia di San Paolo for the EPIC-Italy and EPICOR projects, the Italian Institute for Genomic Medicine (IIGM, formerly Human Genetics Foundation-Torino, HuGeF, Turin, Italy) and the MIUR ex60% grant. EPIC-Italy is further supported by a grant from the Associazione Italiana per la Ricerca sul Cancro (AIRC, Milan).

Mark Ian McCarthy M.I.M. MMcC is a Wellcome Senior Investigator and an NIHR Senior Investigator.
Sarah E Medland S.E.M. SEM was funded by an NHMRC Senior Reseach Fellowship (APP1103623)
Andres Metspalu A.Metspalu EGCUT was supported by Estonian Research Council [IUT20-60, IUT24-6, PUT1660 to T.E and PUT1665 to K.F.; European Union Horizon 2020 [692145]; European Union through the European Regional Development Fund [2014-2020.4.01.15-0012 GENTRANSMED];
Grant W Montgomery G.W.M. G.W.M. was supported by the NHMRC Fellowships Scheme (339446, 619667)
Dennis Mook-Kanamori D.M.-K. The authors of the NEO study thank all individuals who participated in the Netherlands Epidemiology in Obesity study, all participating general practitioners for inviting eligible participants and all research nurses for collection of the data. We thank the NEO study group, Pat van Beelen, Petra Noordijk and Ingeborg de Jonge for the coordination, lab and data management of the NEO study. The genotyping in the NEO study was supported by the Centre National de Génotypage (Paris, France), headed by Jean-Francois Deleuze. The NEO study is supported by the participating Departments, the Division and the Board of Directors of the Leiden University Medical Center, and by the Leiden University, Research Profile Area Vascular and Regenerative Medicine. Dennis Mook-Kanamori is supported by Dutch Science Organization (ZonMW-VENI Grant 916.14.023).
Patricia B Munroe P.B.M. This work was funded by the Medical Research Council of Great Britain (grant number: G9521010D). The BRIGHT study is extremely grateful to all the patients who participated in the study and the BRIGHT nursing team. We wish to acknowledge the support of the NIHR Cardiovascular Biomedical Research Centre at Barts and Queen Mary University of London, UK
Kari E North K.E.N. see ARIC disclosure
Jeffery R O'connell J.R.O'c. The Amish studies are supported by grants and contracts from the NIH, including R01 AG18728, R01 HL088119, U01 GM074518, U01 HL072515-06, U01 HL84756, R01 DK54261, U01 HL137181, , the University of Maryland General Clinical Research Center, grant M01 RR 16500, the Mid-Atlantic Nutrition Obesity Research Center grant P30 DK72488, and by the Baltimore Diabetes Research and Training Center grant P60DK79637, the American Heart Association: 12SDG9280031 and 17GRNT33661168. In addition, this project was supported by National Research Initiative Competitive Grant no. 2007-35205-17883 from the USDA National Institute of Food and Agriculture. We gratefully thank our Amish community and research volunteers for their long-standing partnership in research, and acknowledge the dedication of our Amish liaisons, field workers and the Amish Research Clinic staff, without which these studies would not have been possible.
Carole Ober C.O. NIH Grants R01 HL085197, T32 GM007197, and F31 HL123289.
Walter Palmas W.P. MESA (Multi-Ethnic Study of Atherosclerosis): MESA and the MESA SHARe project are supported by the National Heart, Lung, and Blood Institute (NHLBI) in collaboration with MESA investigators. Support for MESA is provided by contracts HHSN268201500003I, N01-HC-95159, N01-HC-95160, N01-HC-95161, N01-HC-95162, N01-HC-95163, N01-HC-95164, N01-HC-95165, N01-HC-95166, N01-HC-95167, N01-HC-95168, N01-HC-95169, UL1-TR-000040, UL1-TR-001079, UL1-TR-001420, UL1-TR-001881, and DK063491. Funding for SHARe genotyping was provided by NHLBI Contract N02-HL-64278. The provision of genotyping data was supported in part by the National Center for Advancing Translational Sciences, CTSI grant UL1TR001881, and the National Institute of Diabetes and Digestive and Kidney Disease Diabetes Research Center (DRC) grant DK063491 to the Southern California Diabetes Endocrinology Research Center.
Colin Palmer C.Palmer We are grateful to all the participants in this study, the general practitioners, the Scottish School of Primary Care for their help in recruiting the participants, and to the whole team, which includes interviewers, computer and laboratory technicians, clerical workers, research scientists, volunteers, managers, receptionists, and nurses. The study complies with the

Declaration of Helsinki. We acknowledge the support of the Health Informatics Centre, University of Dundee for managing and supplying the anonymised data and NHS Tayside, the original data owner. The Wellcome Trust United Kingdom Type 2 Diabetes Case Control Collection (GoDARTS) was funded by The Wellcome Trust (072960/Z/03/Z, 084726/Z/08/Z, 084727/Z/08/Z, 085475/Z/08/Z, 085475/B/08/Z) and as part of the EU IMI-SUMMIT program.
Etienne Patin E.P. The Milieu Intérieur cohort was supported by the French government's program Investissement d'Avenir, managed by the Agence Nationale de la Recherche (reference 10-LABX-69-01).
Louis Perusse L.P. The Quebec Family Study was funded by multiple grants from the Medical Research Council of Canada and the Canadian Institutes for Health Research. This work was supported by a team grant from the Canadian Institutes for Health Research (FRN-CCT-83028).
Mario Pirastu M.Pirastu We thank the Ogliastra population and all the individuals who participated in this study. We are very grateful to the municipal administrators for their collaboration to the project and for economic and logistic support. This research was supported by grants from the Italian Ministry of Education, University and Research (MIUR) no.5571/DSPAR/2002 and (FIRB) D. M. no. 718/Ric/2005.
David J Porteous D.J.P. Generation Scotland received core support from the Chief Scientist Office of the Scottish Government Health Directorates [CZD/16/6] and the Scottish Funding Council [HR03006]. Genotyping of the GS:SFHS samples was carried out by the Genetics Core Laboratory at the Wellcome Trust Clinical Research Facility, Edinburgh, Scotland and was funded by the Medical Research Council UK and the Wellcome Trust (Wellcome Trust Strategic Award STratifying Resilience and Depression Longitudinally (STRADL) Reference 104036/Z/14/Z).
Danielle Posthuma D.P. The Netherlands Organization for Scientific Research (NWO VICI 435-14-005. Analyses were carried out on the Genetic Cluster Computer, which is financed by the Netherlands Scientific Organization (NWO: 480-05-003 awarded to DP), by the VU University, Amsterdam, the Netherlands, and by the Dutch Brain Foundation, and is hosted by the Dutch National Computing and Networking Services SurfSARA.
Bruce M Psaty B.M.P. Cardiovascular Health Study: This CHS research was supported by NHLBI contracts HHSN268201200036C, HHSN268200800007C, HHSN268201800001C, N01HC55222, N01HC85079, N01HC85080, N01HC85081, N01HC85082, N01HC85083, N01HC85086; and NHLBI grants U01HL080295, R01HL087652, R01HL105756, R01HL103612, R01HL120393, and R01HL130114 with additional contribution from the National Institute of Neurological Disorders and Stroke (NINDS). Additional support was provided through R01AG023629 from the National Institute on Aging (NIA). A full list of principal CHS investigators and institutions can be found at CHS-NHLBI.org. The provision of genotyping data was supported in part by UL1TR000124, and DK063491.
Bruce M Psaty B.M.P. The content is solely the responsibility of the authors and does not necessarily represent the official views of the National Institutes of Health.
John D Rioux J.D.R. JDR acknowledges financial support from the Montreal Heart Institute Foundation
Fernando Rivadeneira F.Rivadeneira The Netherlands Organization for Health Research and Development supported FR (ZonMw VIDI 016.136.367)
Charles Rotimi C.R. The AADM and HUFS studies were supported in part by the Intramural Research Program of the National Institutes of Health in the Center for Research on Genomics and Global Health (CRGGH). The CRGGH is supported by the National Human Genome Research Institute (NHGRI), the National Institute of Diabetes and Digestive and Kidney Diseases (NIDDK), the Center for Information Technology, and the Office of the Director at the National Institutes of Health (1ZIAHG200362).
Jerome I Rotter J.I.R. MESA (Multi-Ethnic Study of Atherosclerosis): MESA and the MESA SHARe project are supported by the National Heart, Lung, and Blood Institute (NHLBI) in collaboration with MESA investigators. Support for MESA is provided by contracts HHSN268201500003I, N01-HC-95159, N01-HC-95160, N01-HC-95161, N01-HC-95162, N01-HC-95163, N01-HC-95164, N01-HC-

95165, N01-HC-95166, N01-HC-95167, N01-HC-95168, N01-HC-95169, UL1-TR-000040, UL1-TR-001079, UL1-TR-001420, UL1-TR-001881, and DK063491. Funding for SHARe genotyping was provided by NHLBI Contract N02-HL-64278. The provision of genotyping data was supported in part by the National Center for Advancing Translational Sciences, CTSI grant UL1TR001881, and the National Institute of Diabetes and Digestive and Kidney Disease Diabetes Research Center (DRC) grant DK063491 to the Southern California Diabetes Endocrinology Research Center.

Igor Rudan I.R. None

Dharambir K Sanghera D.K.S. This work was supported by NIH grants -R01DK082766 funded by the National Institute of Health (NIDDK) and NOT-HG-11-009 funded by NHGRI, VPR Bridge Grant and Enrichment Grant from Harold Hamm Diabetes Center from University of Oklahoma Health Sciences Center. Authors thank all the participants of AIDHS/SDS and are grateful for their contribution in this study.

Matthias B Schulze M.B.S. The study was supported in part by a grant from the German Federal Ministry of Education and Research (BMBF) to the German Center for Diabetes Research (DZD e.V.) and the State of Brandenburg. The recruitment phase of the EPIC-Potsdam study was supported by the Federal Ministry of Science, Germany (01 EA 9401) and the European Union (SOC 95201408 05 F02). The follow-up of the EPIC-Potsdam study was supported by German Cancer Aid (70-2488-Ha I) and the European Community (SOC 98200769 05 F02).

Matthias B Schulze M.B.S. Exome chip genotyping of EPIC-Potsdam samples was carried out under supervision of Per Hoffmann and Stefan Herms at Life & Brain GmbH, Bonn. We are grateful to the Human Study Centre (HSC) of the German Institute of Human Nutrition Potsdam-Rehbrücke, namely the trustee and the data hub for the processing, and the participants for the provision of the data, the biobank for the processing of the biological samples and the head of the HSC, Manuela Bergmann, for the contribution to the study design and leading the underlying processes of data generation.

Alan R Shuldiner A.R.S. The Amish studies are supported by grants and contracts from the NIH, including R01 AG18728, R01 HL088119, U01 GM074518, U01 HL072515-06, U01 HL84756, R01 DK54261, U01 HL137181, , the University of Maryland General Clinical Research Center, grant M01 RR 16500, the Mid-Atlantic Nutrition Obesity Research Center grant P30 DK72488, and by the Baltimore Diabetes Research and Training Center grant P60DK79637, the American Heart Association: 12SDG9280031 and 17GRNT33661168. In addition, this project was supported by National Research Initiative Competitive Grant no. 2007-35205-17883 from the USDA National Institute of Food and Agriculture. We gratefully thank our Amish community and research volunteers for their long-standing partnership in research, and acknowledge the dedication of our Amish liaisons, field workers and the Amish Research Clinic staff, without which these studies would not have been possible.

Nicholas J Timpson N.J.T. NJT is a Wellcome Trust Investigator (202802/Z/16/Z), is the PI of the Avon Longitudinal Study of Parents and Children (MRC & WT 102215/2/13/2), is supported by the University of Bristol NIHR Biomedical Research Centre (BRC) and works within the CRUK Integrative Cancer Epidemiology Programme (C18281/A19169).

Daniela Toniolo D.T. We thank all the participants to the Val Borbera project, prof. Clara Camaschella who coordinated the data collection, Corrado Masciullo and Massimiliano Cocca for the database informatics. The research was supported by funds from Compagnia di San Paolo, Torino, Italy; Fondazione Cariplo, Italy; Ministry of Health, Ricerca Finalizzata 2011-2012 and Public Health Genomics Project 2010.

David-Alexandre Tregouet D.-A.T. The MARTHA genetics project is supported by the GENMED Laboratory of Excellence on Medical Genomics (ANR-10-LABX-0013), the French Clinical Research Infrastructure Network on Venous Thrombo-Embolism (F-CRIN INNOVTE) and the ICAN Institute for Cardiometabolism and Nutrition (ANR-10-IAHU-05), three research programs managed by the National Research Agency (ANR) as part of the French Investment for the Future initiative.

Tiinamaija Tuomi T.T. The Botnia studies (T.T) Folkhälsan Research Foundation, The Academy of Finland (grant no. 312072), Ollqvist Foundation, Swedish Cultural Foundation in Finland, Finnish Diabetes Research Foundation, Foundation for Life and Health in Finland, Signe and Ane Gyllenberg Foundation, Finnish Medical Society, Paavo Nurmi Foundation, Helsinki University Central Hospital Research Foundation, Perklén Foundation, Närpes Health Care Foundation and Ahokas Foundation. The study has also been supported by the Ministry of Education in Finland, Municipal Heath Care Center and Hospital in Jakobstad and Health Care Centers in Vasa, Närpes and Korsholm. The skillful assistance of the Botnia Study Group is gratefully acknowledged.
Peter Vollenweider P.V. CoLaus (Cohorte Lausannoise): The CoLaus study was and is supported by research grants from GlaxoSmithKline, the Faculty of Biology and Medicine of Lausanne, and the Swiss National Science Foundation (grants 33CSCO-122661, 33CS30-139468 and 33CS30-148401).
Carol A Wang C.A.W. This study was supported by the National Health and Medical Research Council of Australia [grant numbers 572613, 403981 and 003209] and the Canadian Institutes of Health Research [grant number MOP-82893]. The authors are grateful to the Raine Study participants and their families, and to the Raine Study research staff for cohort coordination and data collection. The authors gratefully acknowledge the NH&MRC for their long term contribution to funding the study over the last 29 years and also the following Institutions for providing funding for Core Management of the Raine Study: The University of Western Australia (UWA), Curtin University, Raine Medical Research Foundation, UWA Faculty of Medicine, Dentistry and Health Sciences, The Telethon Institute for Child Health ResearchKids Institute, and Women and Infants Research Foundation (King Edward Memorial Hospital), Murdoch University, The University of Notre Dame (Australia), and Edith Cowan University.. The authors gratefully acknowledge the assistance of the Western Australian DNA Bank (National Health and Medical Research Council of Australia National Enabling Facility). This work was supported by resources provided by the Pawsey Supercomputing Centre with funding from the Australian Government and the Government of Western Australia
David R Weir D.R.W. NIA U01AG009740
John Wright J.W. BiB receives core infrastructure funding from Wellcome (WT101597MA), a joint grant from the Medical Research Council (MR/N024397/1)and the National Institute for Health Research Collaboration for Applied Health Research and Care for Yorkshire and Humber.
Markus Perola M.Perola The EU FP7 under grant agreement HZ2020 633589 (Ageing with Elegans)
Markus Perola M.Perola The Finnish Academy grant no. 269517
Markus Perola M.Perola The Yrjö Jahnsson Foundation
Markus Perola M.Perola The Finnish Foundation for Cardiovascular Research
Patrik KE Magnusson P.K.M. The Swedish Twin Registry is managed by Karolinska Institutet and receives funding through the Swedish Research Council under the grant no 2017-00641.
Daniel I Chasman D.I.C. The WGHS is supported by the National Heart, Lung, and Blood Institute (HL043851 and HL080467) and the National Cancer Institute (CA047988 and UM1CA182913) with funding for genotyping provided by Amgen.
Ruth J F Loos R.J.F.L. Ruth Loos is supported by the National Institutes of Health [R01 DK107786, R01 DK110113, U01HG007417].
Ruth J F Loos R.J.F.L. The Mount Sinai BioMe Biobank is supported by The Andrea and Charles Bronfman Philanthropies.
Nora Franceschini N.F. The ARIC study is carried out as a collaborative study supported by National Heart, Lung, and Blood Institute contracts (HHSN268201100005C, HHSN268201100006C, HHSN268201100007C, HHSN268201100008C, HHSN268201100009C, HHSN268201100010C, HHSN268201100011C, and HHSN268201100012C), R01HL087641, R01HL59367 and R01HL086694; National Human Genome Research Institute contract U01HG004402; and National Institutes of Health contract HHSN268200625226C. The authors thank the staff and participants of the ARIC study for their important contributions. Infrastructure was partly supported by Grant Number

UL1RR025005, a component of the National Institutes of Health and NIH Roadmap for Medical Research. NF is supported by the National Institute of Health R01-MD012765,R21-HL140385,R21-HL123677 and R56-DK104806.
Caroline Hayward C.H. We would like to acknowledge the contributions of Professor Pavao Rudan and staff of the Institute for Anthropological Research in Zagreb, the Croatian Centre for Global Health, University of Split, the recruitment teams in Vis and Korcula, the administrative teams in Croatia and Edinburgh and the people of Vis and Korcula. The SNP genotyping for the CROATIA_Korcula cohort was performed either by Helmholtz Zentrum München, Neuherberg, Germany or the Clinical Research Facility, University of Edinburgh. The SNP genotyping for the CROATIA_Vis cohort was performed by the Clinical Research Facility, University of Edinburgh. The work was funded by the Medical Research Council (UK) core funding to the QTL in Health and Disease programme, the European Union framework program 6 EUROSPAN project (contract no. LSHG-CT-2006-018947) and the Ministry of Science, Education and Sport in the Republic of Croatia (number 108-1080315-0302).
Tõnu Esko T.E. EGCUT was supported by Estonian Research Council [IUT20-60, IUT24-6, PUT1660 to T.E and PUT1665 to K.F.; European Union Horizon 2020 [692145]; European Union through the European Regional Development Fund [2014-2020.4.01.15-0012 GENTRANSMED];

SUPPLEMENTARY NOTE 3: The SIGMA Type 2 Diabetes Genetics Consortium members

Genetic analyses: Josep M. Mercader[1, 2, 3], Alicia Huerta-Chagoya[4], Humberto García-Ortiz[5], Hortensia Moreno-Macías[4, 6], Alisa Manning[3, 7, 8], Lizz Caulkins[3], Noël P. Burtt[3], Jason Flannick[3, 9], Nick Patterson[10], Carlos A. Aguilar-Salinas[4], Teresa Tusié-Luna[4, 11], David Altshuler[3, 9, 12], Jose C. Florez[1, 3, 8, 13]

Study cohorts: *Diabetes in Mexico Study:* Humberto García-Ortiz[5], Angélica Martínez-Hernández[5], Federico Centeno-Cruz[5], Francisco Martin Barajas-Olmos[5], Carlos Zerrweck[14], Cecilia Contreras-Cubas[5], Elvia Mendoza-Caamal[5], Cristina Revilla-Monsalve[15], Sergio Islas-Andrade[15], Emilio Córdova[5], Xavier Soberón[5], Lorena Orozco[5]. *Mexico City Diabetes Study:* Clicerio González-Villalpando[16], María Elena González-Villalpando[16]. *Multiethnic Cohort Study:* Christopher A. Haiman[17], Lynne Wilkens[18], Loic Le Marchand[18], Kristine Monroe[17], Laurence Kolonel[18]. *UNAM/INCMNSZ Diabetes Study:* Olimpia Arellano-Campos[4], Alicia Huerta-Chagoya[4], Maria L. Ordóñez-Sánchez[4], Maribel Rodríguez-Torres[4], Yayoi Segura-Kato[4], Rosario Rodríguez-Guillén[4], Ivette Cruz-Bautista[4], Linda Liliana Muñoz-Hernandez[4], Tamara Sáenz[4], Donají Gómez[4], Ulices Alvirde[4], Paloma Almeda-Valdés[4], Hortensia Moreno-Macías[4, 6], Teresa Tusié-Luna[4, 11], Carlos A. Aguilar-Salinas[4]

Scientific and project management: Noël P. Burtt[3], Lizz Caulkins[3], Maria L. Cortes[10]

Steering committee: David Altshuler[3, 9, 12], Jose C. Florez[1, 3, 8, 13], Christopher A. Haiman[17], Carlos A. Aguilar-Salinas[4], Clicerio González-Villalpando[16], Lorena Orozco[5], Teresa Tusié-Luna[4, 11]

[1] Diabetes Unit and Center for Human Genetic Research, Massachusetts General Hospital, Boston, Massachusetts, 02114, USA.

[2] Joint BSC-CRG-IRB Research Program in Computational Biology. Barcelona Supercomputing Center, 08034 Barcelona.

[3] Program in Medical and Population Genetics, Broad Institute of Harvard and MIT, Cambridge, Massachusetts, 02142, USA.

[4] Instituto Nacional de Ciencias Médicas y Nutrición Salvador Zubirán, Sección XVI, Tlalpan, 14000 Mexico City, Mexico.

[5] Instituto Nacional de Medicina Genómica, Tlalpan, 14610, Mexico City, Mexico.

[6] Universidad Autónoma Metropolitana, Tlalpan 14387, Mexico City, Mexico.

[7] Center for Human Genetic Research, Massachusetts General Hospital, Boston, Massachusetts, 02114, USA.

[8] Department of Medicine, Harvard Medical School, Boston, Massachusetts, USA.

[9] Department of Molecular Biology, Massachusetts General Hospital, Boston, Massachusetts, 02114, USA.

[10] Broad Institute of Harvard and MIT, Cambridge, Massachusetts, 02142, USA.

[11] Instituto de Investigaciones Biomédicas, UNAM Unidad de Biología Molecular y Medicina Genómica, UNAM/INCMNSZ, Coyoacán, 04510 Mexico City, Mexico.

[12] Department of Genetics, Harvard Medical School, Boston, Massachusetts, 02115, USA.

[13] Metabolism Program, Broad Institute of Harvard and MIT, Cambridge, Massachusetts, 02142, USA.

[14] Clínica de Integral de Cirugía para la Obesidad y Enfermedades Metabólicas, Hospital General Tláhuac, Secretaría de Salud del GDF. México City.

[15] Instituto Mexicano del Seguro Social SXXI, Mexico City, Mexico.

[16] Centro de Estudios en Diabetes, Unidad de Investigacion en Diabetes y Riesgo Cardiovascular, Centro de Investigacion en Salud Poblacional, Instituto Nacional de Salud Publica, Mexico City, Mexico.

[17] Department of Preventive Medicine, Keck School of Medicine, University of Southern California, Los Angeles, California, 90033, USA.

[18] Epidemiology Program, University of Hawaii Cancer Center, Honolulu, Hawaii, USA.

SUPPLEMENTARY NOTE 4: Comparison of inbreeding coefficient estimates

Introduction. Accurate estimation of the Inbreeding Depression (β_F) requires precise and unbiased estimates of individual inbreeding coefficients (F). Numerous methods have been proposed for estimating inbreeding coefficients from dense SNP marker data. Broadly, these can be grouped into two categories: methods that consider the excess homozygosity at a large number of, preferably independent, markers (e.g. F_{HOM}, F_{UNI}) and methods that identify autozygous genomic segments from unbroken tracts of homozygous genotypes, which are unlikely to occur by chance (e.g. F_{ROH}).

To maximize statistical power and minimize bias in $\hat{\beta}_F$, an estimator of F should be both unbiased, ($E[\hat{F}] = F$) and precise, $MSE(\hat{F} - F) \ll var(F)$. Extensive comparisons of different estimators have been made by previous studies. Many of these conclude that F_{ROH} calculated from appropriately parameterized ROH calling gives estimates of F with minimal bias and lower variance than independent SNP methods[1–4]. In contrast, Yengo et al. (PNAS 2017) claim that $\hat{\beta}_{F_{\text{ROH}}}$ may be upwardly biased by as much as 162%, and provide apparent evidence of this in both simulated and real data[5]. Furthermore, Yengo et al. show, in both theory and simulation, that two independent SNP methods (F_{UNI} and F_{HOM}) give unbiased estimates of β_F when causal SNPs are a random subset of all genotyped SNPs. This would cast doubt on the validity of using F_{ROH} to estimate β_F, and suggests F_{UNI} might be a more appropriate measure. To understand the apparent contradictions between different studies, we have repeated and extended the investigations described in Yengo et al.

Yengo et al. base their study on genotype data derived from the first phase of the UK Biobank (UKB) imputation. 9,493,148 SNPs with a minor allele frequency (MAF) of >1%, INFO score of > 0.3 and

HWE p-value > 1e-06 were selected from the imputation of 140,720 British individuals. SNP dosages were rounded to the nearest whole genotype and LD pruning r^2> 0.9 was performed to reduce the number of SNPs to 3,857,369. F_{UNI} was calculated by the formula presented by Yang et al. (2011)[6] as $\hat{F}^{III}$ (implemented in PLINK[7] by parameters --ibc Fhat3) and ROH were called by two PLINK parameterizations, including the values proposed in Joshi et al. (2015)[8]. Joshi et al. validated the PLINK parameters for moderately dense SNP chips, but not for the different characteristics (SNP density, error rate) of genotypes called from imputed dosages. In particular, the PLINK method allows only one heterozygote or 5 missing genotypes within each ROH.

F_{ROH} calculated from SNP chip genotypes agrees well with F_{UNI} indicating minimal bias. We followed the method described in Yengo et al. to calculate both F_{UNI} and F_{ROH} from the UKB imputation, but initially compared Yengo's F_{UNI} to our F_{ROH} calculated from SNP chip data available for the same phase one individuals. From now on, we use lowercase roh to refer to measures based on imputed data and uppercase ROH to refer to those from SNP-chip genotypes. We find good correspondence between F_{UNI} and F_{ROH} (Supplementary Fig. 12). Since F_{UNI} is believed to be an unbiased estimator of F, the correspondence between F_{UNI} and F_{ROH} places limits on the possible bias of F_{ROH}. Specifically, if we model F_{ROH} as $F_i^{\text{ROH}} = \Theta F_i + \epsilon_i$, then

$$\frac{r^2}{\beta_{F_{\text{UNI}},F_{\text{ROH}}}} \leq \Theta \leq \frac{1}{\beta_{F_{\text{UNI}},F_{\text{ROH}}}} \tag{19}$$

(see Supplementary note 6). Substituting the regression values from Supplementary Fig. 12 gives $0.94 \leq \Theta \leq 0.95$. I.e. the good correspondence between F_{UNI} and F_{ROH} limits the potential error in F_{ROH} to a small downward bias. This very small underestimate of F may be caused by autozygosity not captured in F_{ROH}, namely ROH of less than 1.5 Mb length or ROH in sparsely genotyped regions.

Calculating F_{roh} from imputed genotypes can introduce large downward bias. To call ROH from the UKB imputation we followed the method described by Yengo et al., but identified only 9,067,605 SNPs (out of a total of 72,355,667) matching their inclusion criteria (MAF > 1%, INFO > 0.3, HWE exact p-value > 1e-6). We then used PLINK 2.0 to convert the imputed genotype probabilities to the hard-called genotypes required for LD pruning and ROH calling. PLINK first converts genotype probabilities to an estimated dosage which is then rounded to the nearest genotype ϵ [0 1 2]. By default, a genotype is recorded if the estimated dosage is within 0.15 of any of [0 1 2], otherwise the genotype is recorded as missing. Using these default parameters introduces 3.9% missingness into our dataset. We nevertheless proceeded to LD-prune this dataset, both with these missing data (method 2), and having removed all SNPs with a missing fraction > 0.03 (method 1). However, Yengo et al. state that *Imputed SNPs were called to the genotypes having the largest posterior probability* which is achieved by changing the PLINK hardcall parameter from 0.15 to 0.499999 (method Yengo). After LD pruning 3,061,484 SNPs remain for 141,774 British individuals.

For all three methods of data preparation, we called ROH and calculated F_{roh} using the Joshi et al. PLINK parameters also used by Yengo et al. We find that calling ROH from hard-called imputed dosages (method Yengo) gives F_{roh} with an expected value of just 0.39 of the F_{ROH} obtained from SNP chip genotypes (Supplementary Figure 13). More stringent treatments of uncertain genotype probabilities (methods 2 and 1) give progressively less downwards bias. To understand the cause of this downward bias in F_{roh} we plotted the genome wide distribution of ROH for two high F individuals, highlighted in orange in Supplementary Figure 13.

For these two individuals all ROH called from SNP chip genotypes (in blue) and the Yengo et al. imputed data method (in red) are shown in Supplementary Figs 14a,b. Individual 1 has an $F_{\text{ROH}} =$

0.261, amongst the highest observed in UKB, and is most likely the progeny of 1st degree relatives where $E[F] = 0.25$. Individual 2 has $F_{\mathrm{ROH}} = 0.0626$ and is most likely the offspring of first cousins (3rd degree relatives) where $E[F] = 0.0625$. Calling ROH from hard-called imputed dosages fragments, and consequently fails to identify, many of the ROH found in SNP chip data. The resultant downward bias in F_{roh} is sufficient to explain an upward bias of up to 156% (1/0.39) in $\hat{\beta}_{F_{\mathrm{roh}}}$.

In summary, calculating F_{ROH} from dense SNP chip genotypes, with the parameters used in this study, gives valid estimates of inbreeding coefficients. In contrast, calculating F_{roh} from unfiltered imputed genotypes, as done in Yengo et al., introduces a large bias which appears to be responsible for the poor performance of F_{roh} in that study.

$\hat{\boldsymbol{\beta}}_{F_{\mathrm{GRM}}}$ is downwardly biased in real data. Yengo et al. also show, in both theory and simulation, that $\hat{\beta}_{F_{\mathrm{UNI}}}$ is an unbiased estimate of β_F in certain conditions, for example, when causal SNPs are a random subset of all observed SNPs. In real UKB data they find that $\hat{\beta}_{F_{\mathrm{roh}}}$ is systematically of greater magnitude than $\hat{\beta}_{F_{\mathrm{UNI}}}$, which they therefore interpret as empirical evidence that $\hat{\beta}_{F_{\mathrm{ROH}}}$ is upwardly biased. We have already shown that calling ROH from imputed data may cause an upward bias of $\hat{\beta}_{F_{\mathrm{roh}}}$, however, interestingly, we also observe that estimates obtained from unbiased SNP-chip genotypes ($\hat{\beta}_{F_{\mathrm{ROH}}}$) are systematically larger than estimates obtained from frequency-based measures ($\hat{\beta}_{F_{\mathrm{SNP}}}$ and $\hat{\beta}_{F_{\mathrm{GRM}}}$) (Supplementary Data Table 13). Note, we use the nomenclature F_{GRM} to refer the $\hat{F}^{III}$ calculation used in the ROHgen consortium. Although F_{GRM} and F_{UNI} are identical calculations (PLINK –ibc Fhat3), F_{GRM} is calculated from SNP-chip genotypes with a minimum MAF of 5%, while Yengo et al. calculated F_{UNI} from hard called imputed dosages with a minimum MAF of 1%. We explain the differences between $\hat{\beta}_{F_{\mathrm{ROH}}}$ and $\hat{\beta}_{F_{\mathrm{GRM}}}$ below.

Causal variants for Inbreeding Depression are not in strong LD with common SNPs. For all traits, we fit bivariate models with F_{ROH} and F_{GRM} as explanatory variables. For all 32 traits that were significant in the univariate analysis, we find that $\hat{\beta}_{F_{\mathrm{ROH}}|F_{\mathrm{GRM}}}$ is of greater magnitude than $\hat{\beta}_{F_{\mathrm{GRM}}|F_{\mathrm{ROH}}}$ in the conditional analysis (Supplementary Data Table 22). Furthermore, for 30 of these traits $\hat{\beta}_{F_{\mathrm{GRM}}|F_{\mathrm{ROH}}}$ does not differ significantly from zero. I.e., for many traits, the variation of F_{GRM} which is independent of F_{ROH} is not associated with any change in trait values. In Supplementary Note 5 we show that these results are consistent with inbreeding depression caused by rare, but not common, variants. Furthermore, we observe that the downward bias of $\hat{\beta}_{F_{\mathrm{GRM}}}$ is proportional to the ratio $\frac{var(F_{\mathrm{ROH}})}{var(F_{\mathrm{GRM}})}$ (Fig 4c), as expected when the difference between F_{GRM} and F_{ROH} can be considered as estimation error (See Supplementary Note 7).

In summary, Yengo et al showed that $\hat{\beta}_{F_{\mathrm{UNI}}}$ is unbiased when causal variants are a random subset of the observed SNPs. Although we agree with this statement, we find the evidence does not support the assumption of a random sample, but reveals the importance of rare variants, whose excess homozygosity is well predicted by F_{ROH} (Supplementary Fig 16a).

Comparison of genomic measures of inbreeding with genealogy. As a further assessment of the relative abilities of F_{ROH}, F_{SNP} and F_{GRM} to capture inbreeding, we analysed Pearson's product-moment correlations between the genomic inbreeding measures and pedigree inbreeding (F_{PED}) for 47,927 Icelanders with mostly-complete (info score > 0.6)[9] 10 generation pedigrees. To decrease the confounding effects of pedigree mis-specification, a small number of individuals (n=20) with extreme discrepancies between genetics and genealogy ($F_{\mathrm{ROH}} > 0.05$ & $F_{\mathrm{SNP}} < 0.001$) were removed. The

correlation was highest for F_{ROH} (r = 0.779), lowest for F_{SNP} (0.632) and intermediate for F_{GRM} (0.682), further validating the utility of F_{ROH} as the most accurate genomic measure of inbreeding.

SUPPLEMENTARY NOTE 5: Interpretation of $Trait \sim F_{\text{ROH}} + F_{\text{GRM}}$ models.

Are inbreeding effects caused by rare or common variants? F_{ROH} is an estimate of autozygosity, which increases the homozygosity of all variants, both common and rare. In contrast, F_{GRM} is calculated from common SNPs (>5% MAF) and correlates well with the homozygosity of common SNPs, but less well with rare SNPs which may be in weak Linkage Disequilibrium (LD). We therefore performed bivariate models of all traits in real data ($Trait \sim F_{\text{ROH}} + F_{\text{GRM}}$) to establish whether the observed inbreeding effects associate more strongly with F_{ROH} or F_{GRM}. For all significant traits, we find the observed associations more attributable to F_{ROH} (Supplementary Data Table 22; Supplementary Figs 15a,b) suggesting inbreeding effects are caused by rare genetic variants. A recent study[10] found evidence for a similar conclusion, but to further support this interpretation we investigate below how both F_{ROH} and F_{GRM} predict the excess homozygosity of SNPs at a range of allele frequencies.

Relationships between F_{ROH}, F_{GRM} and excess homozygosity at different allele frequencies. For any trait exhibiting inbreeding depression, the degree of depression will be related to the excess homozygosity (above Hardy-Weinberg expectation) of the causal variants. In Supplementary note 8, we show that inbreeding depression, which is equal to the sum of the dominance deviations at the causal loci, is proportional to the inbreeding coefficient (F_{QTL}) defined in equation (39) below.

$$ID_i = \sum_{i=1}^{m} \delta_i = \beta_F * F_{\text{QTL}} \tag{38}$$

Where

$$F_{\text{QTL}} = \frac{1}{m}\sum_{i=1}^{m} \frac{w_i\left(x_i^2 - (1+2p_i)x_i + 2p_i^2\right)}{2p_i q_i} \tag{39}$$

and

$$w_i = \frac{2p_i q_i d_i}{\frac{1}{m}\sum_{i=1}^{m} 2p_i q_i d_i} \tag{40}$$

We note that the unweighted form of equation (39) is identical to $\hat{F}^{III}$ introduced by Yang et al (2011)[6], and implemented in PLINK by the parameters –ibc Fhat3. This is the same formula used to calculate F_{GRM} and F_{UNI} from different sets of marker SNPs. In summary, if the causal loci and effect sizes are known, a weighted calculation of $\hat{F}^{III}$ at the causal loci is directly proportional to the degree of inbreeding depression. We have used this, below, to simplify the simulation of inbreeding depression caused by variants at specific allele frequencies.

If we imagine inbreeding depression caused exclusively by variants at one allele frequency then, in the absence of strong selection or assortative mating on the causal loci in the current generation, the expectation of F_{QTL} will be equal to $\hat{F}^{III}$ calculated at marker variants of the same allele frequency (henceforth called F_{MAF}).

To calculate F_{MAF} across a range of allele frequencies we extracted SNPs at seven frequencies (MAF=0.01, 0.025, 0.05, 0.1, 0.2, 0.4 & 0.5) from 402,559 genetically British samples in the phase 2 UKB imputation. Selected SNPs were required to have a minor allele frequency (AF) within 10% of the specified MAF (0.9*MAF < AF < 1.1*MAF) and HWE p-value > 1e-6. The numbers of SNPs retained at each MAF are reported in Supplementary Table 2. F_{ROH} and F_{GRM} had previously been calculated, from SNP-chip genotypes, as part of the ROHgen meta-analysis.

To investigate the relationships between F_{ROH}, F_{GRM} and F_{MAF} we fit univariate ($F_{\text{MAF}} \sim F_{\text{ROH}}$ and $F_{\text{MAF}} \sim F_{\text{GRM}}$) and bivariate models ($F_{\text{MAF}} \sim F_{\text{ROH}} + F_{\text{GRM}}$) at each allele frequency. In the univariate models we find F_{ROH} to be an unbiased predictor of F_{MAF} across the entire frequency spectrum, while F_{GRM} is downwardly biased, particularly at low MAF (Supplementary Fig. 16a). Despite this downward bias, F_{GRM} is more strongly correlated than F_{ROH} at all MAF > 5% (Supplementary Figure 16b). In the bivariate model F_{GRM} is a stronger predictor of the homozygosity of common SNPs (>10%), but F_{ROH} is a stronger predictor for rare SNPs (Fig. 4d).

Observed associations consistent with the homozygosity of rare, not common, SNPs. In real data models of $Trait \sim F_{\text{ROH}} + F_{\text{GRM}}$, we consistently find the observed associations are preferentially attributed to F_{ROH} rather than F_{GRM} (Supplementary Data Table 22, Figure 4c, Supplementary Figs 15a,b). In light of Figure 4d, these results are compatible with the action of rare, not common, causal variants.

SUPPLEMENTARY NOTE 6: Limits of bias in F_{ROH}

If F_{UNI} is an unbiased estimate of F then it can be expressed as

$$F_{\text{UNI}} = F + \varepsilon \tag{20}$$

If F_{ROH} is a potentially biased estimate of F then it can be expressed as

$$F_{\text{ROH}} = \Theta F + \Theta \varepsilon' \tag{21}$$

The regression slope (β) of F_{UNI} on F_{ROH} is known, and

$$\beta = \frac{cov(F_{UNI}, F_{ROH})}{var(F_{ROH})}$$

Substituting (20) and (21) and assuming independent errors gives

$$\beta = \frac{\Theta var(F)}{\Theta^2 var(F) + \Theta^2 var(\varepsilon')} \tag{22}$$

Rearranging (22) gives

$$\Theta = \left(\frac{1}{\beta}\right)\left(1 + \frac{var(\varepsilon')}{var(F)}\right)^{-1} \tag{23}$$

The range of $\frac{var(\varepsilon')}{var(F)}$ is limited by the correlation between F_{UNI} and F_{ROH}, and we can put $\left(1 + \frac{var(\varepsilon')}{var(F)}\right)^{-1}$ in terms $\frac{var(\varepsilon)}{var(\varepsilon')}$ of by considering that

$$\frac{var(F_{\mathrm{ROH}})}{var(F_{\mathrm{GRM}})} = \frac{r^2}{\beta^2} \tag{24}$$

Again substituting (20) and (21) in equation (24) gives

$$\frac{\Theta^2 var(F) + \Theta^2 var(\varepsilon')}{var(F) + var(\varepsilon)} = \frac{r^2}{\beta^2} \tag{25}$$

Rearranging (25) gives

$$\frac{var(\varepsilon')}{var(F)} = \frac{\Theta^2\beta^2 - r^2}{r^2 \dfrac{var(\varepsilon)}{var(\varepsilon')} - \Theta^2\beta^2} \tag{26}$$

Substituting equation (26) into equation (23)

$$\Theta^2 = \left(\frac{1}{\beta}\right)\left(\frac{r^2 \dfrac{var(\varepsilon)}{var(\varepsilon')} - \Theta^2\beta^2}{r^2\left(\dfrac{var(\varepsilon)}{var(\varepsilon')} - 1\right)}\right) \tag{27}$$

As $\frac{var(\varepsilon)}{var(\varepsilon')} \to 0$, i.e. if F_{UNI} is precise and estimation errors entirely on F_{ROH} then equation (27) →

$$\Theta = \left(\frac{1}{\beta}\right)\left(\frac{\Theta^2\beta^2}{r^2}\right) \tag{28}$$

$$\Theta = \frac{r^2}{\beta} \tag{29}$$

As $\frac{var(\varepsilon)}{var(\varepsilon')} \to \infty$, i.e. if F_{ROH} is precise and estimation errors entirely on F_{UNI} then equation (27) →

$$\Theta = \frac{1}{\beta} \tag{30}$$

Therefore, from the bounds of $\frac{var(\varepsilon)}{var(\varepsilon')}$ and equations (29) and (30)

$$\frac{r^2}{\beta_{F_{\mathrm{UNI}},F_{\mathrm{ROH}}}} \leq \Theta \leq \frac{1}{\beta_{F_{\mathrm{UNI}},F_{\mathrm{ROH}}}} \tag{31}$$

SUPPLEMENTARY NOTE 7: Expected attenuation bias in $\hat{\boldsymbol{\beta}}_{F_{\mathrm{GRM}}}$

If F_{GRM} varies around F_{ROH} and the difference (ε) has no effect on the trait (y) then

$$F_{\mathrm{GRM}} = F_{\mathrm{ROH}} + \varepsilon \tag{32}$$

And

$$\beta_{F_{\mathrm{GRM}}} = \frac{cov(F_{\mathrm{GRM}}, y)}{var(F_{\mathrm{GRM}})} \tag{33}$$

$$\beta_{F_{\mathrm{GRM}}} = \frac{cov(F_{\mathrm{ROH}} + \varepsilon, y)}{var(F_{\mathrm{GRM}})} \tag{34}$$

Because ε has no effect on the trait (y)

$$\beta_{F_{\mathrm{GRM}}} = \frac{cov(F_{\mathrm{ROH}}, y)}{var(F_{\mathrm{GRM}})} \tag{35}$$

$$\beta_{F_{\mathrm{GRM}}} = \beta_{F_{\mathrm{ROH}}} * \frac{var(F_{\mathrm{ROH}})}{var(F_{\mathrm{GRM}})} \tag{36}$$

$$\frac{\beta_{F_{\mathrm{GRM}}}}{\beta_{F_{\mathrm{ROH}}}} = \frac{var(F_{\mathrm{ROH}})}{var(F_{\mathrm{GRM}})} \tag{37}$$

SUPPLEMENTARY NOTE 8: Calculation of F_{QTL} at known causal loci.

If $x_i \epsilon [0,1,2]$ is the number of copies of the reference allele at locus i of m causal loci, then the number of reference homozygotes at the locus is $\frac{x_i(x_i-1)}{2}$, the number of heterozygotes is $-x_i(x_i - 2)$, and the number of alternate homozygotes is $\frac{(x_i-2)(x_i-1)}{2}$.

If p_i is the frequency of the reference allele, and q_i is the frequency of the alternate allele, then the inbreeding depression with complete inbreeding (β) is

$$\beta = -\sum_{i=1}^{m} 2p_i q_i d_i \tag{41}$$

Where d_i is the difference between the heterozygote and mean homozygote value. We wish to define an inbreeding coefficient (F_{QTL}) which is directly proportional to realised inbreeding depression (the sum of the dominance deviations). I.e.

$$\beta F_{\mathrm{QTL}} = \sum_{i=1}^{m} \delta_i \tag{42}$$

The dominance deviations (δ_i) for the three genotypes at a locus can be written in terms of d_i: $\delta_i \epsilon \left[-2q_i^2 d_i, 2p_i q_i d_i, -2p_i^2 d_i\right]$. Substituting these dominance deviations and the genotype counts into equation (42) gives

$$\beta F_{\mathrm{QTL}} = \sum_{i=1}^{m} -\frac{x_i(x_i-1)}{2} 2q_i^2 d_i - x_i(x_i-2) 2p_i q_i d_i - \frac{(x_i-2)(x_i-1)}{2} 2p_i^2 d_i \tag{43}$$

Rearranging equation (43) gives

$$\beta F_{\mathrm{QTL}} = \sum_{i=1}^{m} - d_i\left(x_i^2 - (1+2)p_i x_i + 2p_i^2\right) \tag{44}$$

Substituting for β from equation (41) gives

$$F_{\mathrm{QTL}} = \frac{1}{m} \sum_{i=1}^{m} w_i \frac{x_i^2 - (1+2p_i)x_i + 2p_i^2}{2p_i q_i} \tag{45}$$

Where

$$w_i = \frac{2p_iq_id_i}{\frac{1}{m}\sum_{i=1}^{m} 2p_iq_id_i} \quad (46)$$

SUPPLEMENTARY REFERENCES

1. McQuillan, R. *et al.* Runs of Homozygosity in European Populations. *American Journal of Human Genetics* **83**, 359–372 (2008).

2. Keller, M. C., Visscher, P. M. & Goddard, M. E. Quantification of inbreeding due to distant ancestors and its detection using dense single nucleotide polymorphism data. *Genetics* **189**, 237–249 (2011).

3. Kardos, M., Nietlisbach, P. & Hedrick, P. W. How should we compare different genomic estimates of the strength of inbreeding depression? *Proceedings of the National Academy of Sciences* **115**, E2492–E2493 (2018).

4. Gazal, S. *et al.* Inbreeding coefficient estimation with dense SNP data: Comparison of strategies and application to HapMap III. *Human Heredity* **77**, 49–62 (2014).

5. Yengo, L. *et al.* Detection and quantification of inbreeding depression for complex traits from SNP data. *Proceedings of the National Academy of Sciences* **114**, 8602–8607 (2017).

6. Yang, J., Lee, S. H., Goddard, M. E. & Visscher, P. M. GCTA: A tool for genome-wide complex trait analysis. *American Journal of Human Genetics* **88**, 76–82 (2011).

7. Purcell, S. & Chang, C. PLINK 1.9. Available at: www.cog-genomics.org/plink/1.9/.

8. Joshi, P. K. *et al.* Directional dominance on stature and cognition in diverse human populations. *Nature* **523**, 459–462 (2015).

9. Helgason, A., Pálsson, S., Guobjartsson, D. F., Kristjánsson, P. & Stefánsson, K. An association between the kinship and fertility of human couples. *Science* **319**, 813–816 (2008).

10. Johnson, E. C., Evans, L. M. & Keller, M. C. Relationships between estimated autozygosity and complex traits in the UK Biobank. *PLoS Genetics* **14**, (2018).
