# Supplementary material for: Associations of autozygosity with a broad range of human phenotypes
Source: Nat Commun. 2019 Oct 31;10:4957. doi: 10.1038/s41467-019-12283-6 (PMC6823371; doi:10.1038/s41467-019-12283-6)
Supplement: Supplementary file 24 — Description of Additional Supplementary Files [file 41467_2019_12283_MOESM24_ESM.pdf]

Supplementary Data 1: Summary of 234 sub-cohorts contributing to the meta-analysis. *Ancestral group* is given for each sub-cohort, from one of seven broad groups (European, African, South & West Asian, East Asian, Hispanic, Japanese & Mixed). *Number genotyped* is the number of samples in each sub-cohort which pass genotypic Quality Control (QC). *Number of SNPs* is the number of SNPs which pass QC. *Number of samples phenotyped* is the maximum number of samples passing QC for any trait. *Number of phenotypes* is the number of traits (out of a possible 100) contributed by each sub-cohort.

Supplementary Data 2: Details of 234 sub-cohorts contributing to the meta-analysis. Information on cohort recruitment, genotyping, quality control, acknowledgements and funding are presented. PMID, PubMed ID; MAF, minor allele frequency; HWE, Hardy-Weinberg equilibrium *p*-value threshold; SNP, single nucleotide polymorphism.

Supplementary Data 3: Covariates applied in each sub-cohort. For each sub-cohort information is presented about the covariates applied to all traits. *Mixed model*; Although F_{ROH} is not narrow-sense heritable, a mixed model was fitted where significant family structure was known to exist (See Methods). *Number of PCs*; the number of principal components of the Genomic Relationship Matrix (GRM) fitted as covariates. *Other covariates*; any other covariates specific to each cohort, such as assessment centre or genotyping batch.

Supplementary Data 4: Phenotype counts. Number of samples passing QC contributed by each cohort for each trait.

Supplementary Data 5: Homozygosity summary statistics by sub-cohort. Measures of homozygosity are presented for each sub-cohort. *Relative per sample power*; is the approximate power of a sample relative to a single sample from the UK Biobank British cohort (UKBB). This is estimated by $\frac{\sigma^2_{F_{\text{ROH}}}}{\sigma^2_{F_{\text{ROH\_UKBB}}}}$. *Percent total power*; is an estimate of the percentage of the total study power attributable to each cohort. This was calculated from the relative per sample power multiplied by the number of samples phenotyped.

Supplementary Data 6: Correlations between different measures of homozygosity. Correlations are presented between F_{ROH} and three alternative measures of homozygosity.

Supplementary Data 7: Summary of all traits analysed. Full name and units are presented for all traits 100 analysed, along with the total number of samples measured and the number of sub-cohorts contributing each trait.

Supplementary Data 8: Trait modelling. Details of the linear models used are presented for all traits. A trait was only analysed where all *required covariates* were available, however if additional *optional covariates* were also available they were included. A small number of traits were *rank-normalised* before analysis, but then multiplied by the original trait standard deviation to return to trait units.

Supplementary Data 9: Trait summary statistics. Intra-sex means and standard deviations are presented for both sexes, for all quantitative traits. The corresponding values for both sexes combined are weighted by sample size. For binary traits the number of cases (1) and controls (0) are presented for men and women separately, as well as combined.

Supplementary Data 10: $\boldsymbol{\beta_{F_{\text{ROH}}}}$ for all traits. Effect size estimates, standard errors and associated *p*-values are presented for all traits meta-analysed. Traits are ordered by ascending p-value. Effect

estimates are presented in trait units for quantitative traits and ln(Odds-Ratio) from a full logistic model for binary traits.

Supplementary Data 11: $\boldsymbol{\beta_{F_{\mathrm{ROH}}}}$ for all traits normalised by intra-sex standard deviation. To facilitate comparison (see Fig 3a) effect size estimate have been normalised by intra-sex standard deviation. Normalised effect size estimates, normalised standard errors and associated *p*-values are presented for all quantitative traits meta-analysed. Traits are ordered by descending absolute magnitude of normalised effect size. Binary traits are not shown.

Supplementary Data 12: $\boldsymbol{\beta_{F_{\mathrm{ROH}}}}$ estimated for each sex separately. Estimates, standard errors and *p*-values of $\beta_{F_{\mathrm{ROH}}}$ are presented for each sex separately (see Methods). Quantitative trait effect size estimates and standard errors have been normalised by the corresponding intra-sex standard deviation. Binary trait effect estimates are shown as ln(Odds-Ratio) from a linear model approximation (see Methods). Also shown is the *p*-value for heterogeneity of effect between sexes, and traits are presented in ascending order of this *p*-value. Traits only measured in one sex are not shown. *The effect estimates and standard errors for self-declared infertility may be biased by the combination of an extreme case:control ratio and a linear model approximation. See Supplementary Figure 8 for more information.

Supplementary Data 13: Estimates of $\boldsymbol{\beta_{F_{\mathrm{ROH}}}}$, $\boldsymbol{\beta_{F_{\mathrm{SNP}}}}$ and $\boldsymbol{\beta_{F_{\mathrm{GRM}}}}$. Meta-analysed estimates, standard errors and *p*-values are shown for three different estimators of the inbreeding coefficient: F_{ROH}, F_{SNP} and F_{GRM}. All binary trait effect estimates are ln(Odds-Ratio) from the linear model approximation. For all significant traits, estimates of $\beta_{F_{\mathrm{ROH}}}$ are larger than the corresponding estimates of $\beta_{F_{\mathrm{SNP}}}$ and $\beta_{F_{\mathrm{GRM}}}$ and, for height, Fig 4c shows that this attenuation is a function of cohort homozygosity. A recent study by different authors interpreted the differences between $\beta_{F_{\mathrm{ROH}}}$ and $\beta_{F_{\mathrm{SNP}}}$ as evidence of upward bias in estimating inbreeding depression from F_{ROH}; we favour an alternative explanation (See Supplementary Note 2).

Supplementary Data 14: Estimates of $\boldsymbol{\beta_{F_{\mathrm{ROH>5Mb}}}}$, $\boldsymbol{\beta_{F_{\mathrm{ROH<5Mb}}}}$ and $\boldsymbol{\beta_{F_{\mathrm{SNP\_OutsideROH}}}}$. Meta-analysed estimates, standard errors and *p*-values are shown for three measures of homozygosity: $F_{\mathrm{SNP\_OusideROH}}$, $F_{\mathrm{ROH<5Mb}}$ and $F_{\mathrm{ROH>5Mb}}$ (See Methods). All binary trait effect estimates are ln(Odds-Ratio) from the linear model approximation. Comparisons of these estimates are shown in Supplementary Figures 11a, b.

Supplementary Data 15: Estimates of $\boldsymbol{\beta_{F_{\mathrm{ROH}}}}$ in the UKB adopted cohort. Effect size estimates, standard errors and *p*-values are shown from a cohort of 7153 self-declared adopted individuals from UK Biobank. A comparison of these effects to the overall population-wide effects is shown in Fig 5c.

Supplementary Data 16: Estimates of the effect of $\boldsymbol{F_{\mathrm{ROH}}}$ within full-sibling families ($\boldsymbol{\beta_{F_{ROH\_wSibs}}}$). Effect size estimates, standard errors, *p*-values and sample size are shown for all traits. The variation of F_{ROH} within full-sibling families is assumed to be independent of any reasonable model of confounding. Mendelian segregation is the only source of F_{ROH} variation between full siblings, and although siblings may experience different environments, environmental differences are not thought to affect Mendelian segregation. Here, sample size is the number of individuals for whom at least one full sibling with a corresponding phenotype was identified (see Methods). A comparison of $\beta_{F_{ROH\_wSibs}}$ with $\beta_{F_{\mathrm{ROH}}}$ is shown in Figure 5d.

Supplementary Data 17: Mean trait residuals in bins of increasing $\boldsymbol{F_{\mathrm{ROH}}}$. Samples were grouped in bins of similar F_{ROH} and mean trait values were calculated within each group (see Methods). The

mean, standard error, *p*-value and sample size of each group are shown for all traits. These data are also plotted in Figures 5a,b and Supplementary Figures 9a-f.

Supplementary Data 18: Effect estimates stratified by cohort ancestry. Effect size estimates, standard errors, *p*-values and sample sizes are shown for seven groupings of cohort ancestry. The cohort groupings are defined in Supplementary Data Table 1.

Supplementary Data 19: Effect estimates stratified by inferred demographic history. Figure 2 was used to infer the demographic history of each cohort (Supplementary Figure 4), and effect estimates were meta-analysed within each group. Effect size estimates, standard errors, *p*-values and sample sizes are shown for each of the four inferred demographic history groups.

Supplementary Data 20: Effect of additional covariates on $\boldsymbol{\beta}_{\boldsymbol{F}_{\mathbf{ROH}}}$. Effect size estimates, standard errors, *p*-values and sample sizes are shown for the base model, as well as models with Educational Attainment (EA) and Religious Participation fitted as covariates. Graphical comparisons of these effect estimates are provided in Supplementary Figs. 10a,b.

Supplementary Data 21: Independent replication of effects reported in Joshi *et al.* (2015). Approximately half of the cohorts contributing to this study also provided data to Joshi *et al.* To formally replicate the effects previously reported there, a meta-analysis was performed using exclusively the cohorts which did not contribute to Joshi et al. (See Supplementary Data Table 1). For the four significant traits previous reported (height, FEV1, cognitive g, and Educational Attainment) effect size estimates, standard errors, *p*-values and sample sizes are shown from this replication meta-analysis.

Supplementary Data 22: Effect estimates from bivariate models of $\boldsymbol{Trait} \sim \boldsymbol{F}_{\mathbf{ROH}} + \boldsymbol{F}_{\mathbf{GRM}}$. Meta-analysed estimates, standard errors and *p*-values are shown for two inbreeding coefficient estimates ($F_{\mathrm{ROH}} + F_{\mathrm{GRM}}$) in a bivariate model (Supplementary Note 5). All binary trait effect estimates are ln(Odds-Ratio) from the linear model approximation. Comparisons of these estimates are shown in Supplementary Figures 15a, b.
