# Supplementary material for: Associations of autozygosity with a broad range of human phenotypes
Source: Nat Commun. 2019 Oct 31;10:4957. doi: 10.1038/s41467-019-12283-6 (PMC6823371; doi:10.1038/s41467-019-12283-6)
Supplement: Supplementary file 25 — Reporting Summary [file 41467_2019_12283_MOESM25_ESM.pdf]

nature research

Corresponding author(s): Jim F Wilson jim.wilson@ed.ac.uk

Last updated by author(s): Jul 3, 2019

Reporting Summary

Nature Research wishes to improve the reproducibility of the work that we publish. This form provides structure for consistency and transparency in reporting. For further information on Nature Research policies, see Authors & Referees and the Editorial Policy Checklist.

Statistics

For all statistical analyses, confirm that the following items are present in the figure legend, table legend, main text, or Methods section.

n/a	Confirmed	
☐	☒	The exact sample size (n) for each experimental group/condition, given as a discrete number and unit of measurement
☐	☒	A statement on whether measurements were taken from distinct samples or whether the same sample was measured repeatedly
☐	☒	The statistical test(s) used AND whether they are one- or two-sided<br>*Only common tests should be described solely by name; describe more complex techniques in the Methods section.*
☐	☒	A description of all covariates tested
☐	☒	A description of any assumptions or corrections, such as tests of normality and adjustment for multiple comparisons
☐	☒	A full description of the statistical parameters including central tendency (e.g. means) or other basic estimates (e.g. regression coefficient) AND variation (e.g. standard deviation) or associated estimates of uncertainty (e.g. confidence intervals)
☐	☒	For null hypothesis testing, the test statistic (e.g. F, t, r) with confidence intervals, effect sizes, degrees of freedom and P value noted<br>*Give P values as exact values whenever suitable.*
☒	☐	For Bayesian analysis, information on the choice of priors and Markov chain Monte Carlo settings
☒	☐	For hierarchical and complex designs, identification of the appropriate level for tests and full reporting of outcomes
☐	☒	Estimates of effect sizes (e.g. Cohen's d, Pearson's r), indicating how they were calculated

Our web collection on statistics for biologists contains articles on many of the points above.

Software and code

Policy information about availability of computer code

Data collection	In each cohort, data was analysed by a suite of custom R scripts written and distributed by the central ROHgen team. A clear analysis plan and user manual document were also provided to each cohort to ensure entirely consistent analyses. As well as internal calculations performed in R (version 3.2.6 or later), the analysis scripts made external calls to commercial software programs PLINK (v1.9 or later) and KING (v2 or later) for specific requirements. The analysis scripts generated numerous summary statistics which were returned to the central ROHgen team for meta-analysis.
Data analysis	Having gathered summary statistics from each cohort, central meta-analysis was performed by a custom R package (wgRohgen2Meta) also written by the central ROHgen team.

For manuscripts utilizing custom algorithms or software that are central to the research but not yet described in published literature, software must be made available to editors/reviewers. We strongly encourage code deposition in a community repository (e.g. GitHub). See the Nature Research guidelines for submitting code & software for further information.

Data

Policy information about availability of data

All manuscripts must include a data availability statement. This statement should provide the following information, where applicable:

- Accession codes, unique identifiers, or web links for publicly available datasets
- A list of figures that have associated raw data
- A description of any restrictions on data availability

The meta-analysed data which support these findings are available in Supplementary Data Tables.
Summary statistics for each cohort are available from the corresponding author upon reasonable request. The cohort-specific data are not publicly available out of respect to the sensitivity surrounding consanguineous unions in some communities. In the majority of cases we do not have consent to share individual specific data, although for UK Biobank this is available on request from https://www.ukbiobank.ac.uk/.

Field-specific reporting

Please select the one below that is the best fit for your research. If you are not sure, read the appropriate sections before making your selection.

☒ Life sciences ☐ Behavioural & social sciences ☐ Ecological, evolutionary & environmental sciences

For a reference copy of the document with all sections, see nature.com/documents/nr-reporting-summary-flat.pdf

Life sciences study design

All studies must disclose on these points even when the disclosure is negative.

Sample size	Previous studies have established that large sample sizes are required to detect changes in human complex traits associated with inbreeding. For example, Joshi et al (2015) used a sample size of up to 240,000 individuals to detect changes associated with inbreeding in four complex traits. Larger sample sizes allow effects to be detected on traits which have previously not been associated with inbreeding. Since this study used genotype and phenotype data which had previously been collected for other purposes, the marginal cost of including additional samples was modest compared to the improved statistical power of a larger sample. To maximize the sample size we established a consortium (called ROHgen) and contacted a large number of cohort administrators known to have collected suitable genotype and phenotype data. A common analysis plan and software pipeline allowed all cohorts to perform consistent and repeatable analyses. In total, 1,392,446 genotyped samples were included in one or more trait analysis. Sample sizes for each trait are detailed in Supplementary Table 4.
Data exclusions	Samples where excluded from an analysis when the trait value exceed pre-defined limits of normal variation. This exclusion removed clearly erroneous values caused by data entry typos. For example, samples with recorded heights <1.0 m or >2.4 m were not included in the analysis of height.
Replication	Since this study is a meta-analysis of numerous independent cohorts, replication is inherent by consideration of the individual cohorts. Replication serves to reduce the likelihood of type one errors caused by: chance, bias or confounding. The extremely small p-values seen in this study make chance an unlikely source of error. The statistical methods of linear regression and inverse variance meta-analysis are well established to be unbiased, and the consistent effect estimates across numerous cohorts of different demographic backgrounds reduces the likelihood of confounding. Furthermore, effect estimates from entirely un-confounded sibling families replicate for two traits and are consistent with the main results for all others.
Randomization	The study design does not allocate samples to groups, but rather uses predefined algorithms to calculate an inbreeding coefficient (FROH) for each individual on a continuous scale from 0 to 1. Randomization is therefore not relevant to the study design, although care was taken to exclude the possibility of spurious associations between the traits of interest and possible confounders. Specifically, covariates were added in the linear regression models including: age, sex, genotyping batch, assessment center, principal components of autosomal genotypes, measures of socio-economic deprivation, etc. Full lists of fitted covariates for each trait and cohort are provided in Supplementary Data Files 3 and 8.
Blinding	The study design does not allocate samples to groups, but rather uses predefined algorithms to calculate an inbreeding coefficient (FROH) for each individual on a continuous scale from 0 to 1. Since the algorithm used to calculate FROH was predefined, investigators were of course completely blind to 'group' allocation.

Reporting for specific materials, systems and methods

We require information from authors about some types of materials, experimental systems and methods used in many studies. Here, indicate whether each material, system or method listed is relevant to your study. If you are not sure if a list item applies to your research, read the appropriate section before selecting a response.

Materials & experimental systems

n/a	Involved in the study	
☒	☐	Antibodies
☒	☐	Eukaryotic cell lines
☒	☐	Palaeontology
☒	☐	Animals and other organisms
☐	☒	Human research participants
☒	☐	Clinical data

Methods

n/a	Involved in the study	
☒	☐	ChIP-seq
☒	☐	Flow cytometry
☒	☐	MRI-based neuroimaging

Human research participants

Policy information about studies involving human research participants

Population characteristics	These are reported in Supplementary Data file 3 and differed for each of the 231 cohorts reported in this meta-analysis. Further details can be found in the cohort descriptive publications, also specified in Supplementary Data file 3.

Recruitment	See above.
Ethics oversight	Usher institute, University of Edinburgh.

Note that full information on the approval of the study protocol must also be provided in the manuscript.
